# Supplementary material for: Reactivity of complex communities can be more important than stability
Source: Nat Commun. 2023 Nov 8;14:7204. doi: 10.1038/s41467-023-42580-0 (PMC10632443; doi:10.1038/s41467-023-42580-0)
Supplement: Supplementary file 1 — Supplementary Information [file 41467_2023_42580_MOESM1_ESM.pdf]

Supplementary Information for

Reactivity of complex communities can be more important than stability

Yuguang Yang, Katharine Z. Coyte, Kevin R. Foster & Aming Li

Contents

1 Supplementary Note 1: Derivation of reactivity criteria 3

1.1 Random community . . . 3

1.2 Community with single interaction type . . . 4

1.2.1 Exploitative community . . . 4

1.2.2 Competitive community . . . 5

1.2.3 Mutualistic community . . . 7

1.3 Community with mixed types of interactions . . . 8

2 Supplementary Note 2: The influence of weak interactions on system reactivity 11

2.1 Comparison between normal distribution & uniform distribution . . . 11

2.2 Reactivity criteria under Gamma distribution . . . 12

3 Supplementary Note 3: Measuring the distance between reactivity and instability 14

3.1 Random community . . . 14

3.2 Exploitative community . . . 15

3.3 Competitive community . . . 16

3.4 Mutualistic community . . . 17

3.5 Community with mixed types of interactions . . . 17

4 Supplementary Note 4: Empirical microbial communities analysed in our work 20

4.1 Mouse microbial communities . . . 20

4.2 Soil microbial communities . . . 20

4.3 Randomly constructed microbial community . . . 20

5 Supplementary Note 5: Influences of empirical food web structures 21

5.1 Cascade food web . . . 21

5.2 Niche food web . . . 21

5.3 Interval cascade food web . . . 22

5.4 Cascade food web with broad degree distribution . . . 23

5.5 Interval cascade food web with broad degree distribution . . . 23

6 Supplementary Note 6: Self-regulation and reactivity of complex ecosystems 25

6.1 Quaternions and the method of quaternionic resolvents . . . 25

6.1.1 Quaternions . . . 25

6.1.2 The method of quaternionic resolvents . . . 26

6.2 Incorporating heterogeneous self-regulation strengths into reactivity analysis . . . 29

6.2.1 Random community . . . 29

6.2.2 Community with mixed types of interactions . . . 33

6.3 Incorporating non-self-regulating species into reactivity analysis . . . 38

6.3.1 Random community . . . 38

6.3.2 Community with mixed types of interactions . . . 40

7 Supplementary Note 7: Correlation between measures of variability, reactivity and asymptotic stability 44

8 Supplementary Note 8: Species abundances and reactivity of complex ecosystems 46

8.1 Rare species tend to control system reactivity . . . 47

8.2 Incorporating heterogeneous species abundances into reactivity analysis . . . 47

8.2.1 Modelling framework . . . 47

8.2.2 Random community . . . 48

8.2.3 Community with mixed types of interactions . . . 50

8.3 The relationship between the reactivity of **A** and the reactivity of **M** . . . 53

8.3.1 Random community . . . 53

8.3.2 Community with mixed types of interactions . . . 54

1 Supplementary Note 1: Derivation of reactivity criteria

1.1 Random community

According to the construction algorithm described in the main text, we can obtain statistics of the community matrix $\mathbf{M}$ for random communities: $\mathbb{E}\left(M_{ij}\right)_{i\neq j} = 0$, $\mathrm{Var}\left(M_{ij}\right)_{i\neq j} = C\sigma^2$, and $\mathbb{E}\left(M_{ij}M_{ji}\right)_{i\neq j} = 0$.

We first ignore the effect of diagonal elements by considering matrix $\mathbf{M}' = \mathbf{M} + d\mathbf{I}$. For sufficiently large communities, the mean, variance and correlation of $\mathbf{M}'$ become $\mathbb{E}\left(M'_{ij}\right) = 0$, $\mathrm{Var}\left(M'_{ij}\right) = C\sigma^2$ and $\mathbb{E}\left(M'_{ij}M'_{ji}\right)_{i\neq j} = 0$. Since $\mathbf{H} = \left(\mathbf{M} + \mathbf{M}^{\mathrm{T}}\right)/2$, we have $\mathbf{H}' = \mathbf{H} + d\mathbf{I} = \left(\mathbf{M}' + \mathbf{M}'^{\mathrm{T}}\right)/2$. The mean, variance and correlation of $\mathbf{H}'$ can be obtained as

$$\begin{cases} \mathbb{E}\left(H'_{ij}\right) = \mathbb{E}\left(\frac{M'_{ij}+M'_{ji}}{2}\right) = 0, \\ \mathrm{Var}\left(H'_{ij}\right) = \mathrm{Var}\left(\frac{M'_{ij}+M'_{ji}}{2}\right) = \frac{1}{2}\mathrm{Var}\left(M'_{ij}\right) = \frac{1}{2}C\sigma^2, \\ \mathbb{E}\left(H'_{ij}H'_{ji}\right)_{i\neq j} = \mathbb{E}\left(H'^2_{ij}\right) = \mathbb{E}\left(\left(\frac{M'_{ij}+M'_{ji}}{2}\right)^2\right) = \frac{1}{2}\mathbb{E}\left(M'^2_{ij}\right). \end{cases} \tag{1}$$

According to the definition of variance, we have

$$\mathrm{Var}\left(M'_{ij}\right) = \mathbb{E}\left(M'^2_{ij}\right) - \mathbb{E}^2\left(M'_{ij}\right), \tag{2}$$

which leads to

$$\mathbb{E}\left(H'_{ij}H'_{ji}\right) = \frac{1}{2}\mathbb{E}\left(M'^2_{ij}\right) = \frac{1}{2}\left(\mathrm{Var}\left(M'_{ij}\right) + \mathbb{E}\left(M'_{ij}\right)\right) = \frac{1}{2}C\sigma^2. \tag{3}$$

Then, consider matrix $\mathbf{N} = \mathbf{H}' \Big/ \sqrt{S\cdot\mathrm{Var}\left(H'_{ij}\right)}$, we can obtain

$$\begin{cases} \mathbb{E}(N_{ij}) = 0, \\ \mathrm{Var}(N_{ij}) = \frac{1}{S}, \\ \mathbb{E}(N_{ij}N_{ji})_{i\neq j} = \frac{1}{S}. \end{cases} \tag{4}$$

Clearly, matrix $\mathbf{N}$ satisfies the elliptic law [1–4]. Thus, the eigenvalues of $\mathbf{N}$ are distributed in an ellipse centered at $(0,0)$ with half horizontal axis $a = 1+\tau$ and half vertical axis $b = 1-\tau$ ($\tau = S\mathbb{E}(N_{ij}N_{ji})_{i\neq j}$). Since $\tau = 1$, the length of half vertical axis becomes 0 and this ellipse degenerates to a line segment on the real axis. The length of this line segment is 4 and the center of this line segment is $(0,0)$. This further brings the eigenvalue distribution of $\mathbf{H}'$: eigenvalues of matrix $\mathbf{H}'$ are contained in a line segment on the real axis. This line segment is centered at $(0,0)$ with length $2\sigma\sqrt{2SC}$.

We now consider the effects of diagonal elements and turn to matrix $\mathbf{H} = \mathbf{H}' - d\mathbf{I}$. Since $H_{ii} = M_{ii} = -d$, the eigenvalue distribution of $\mathbf{H}$ is shifted leftwards (compared to that of $\mathbf{H}'$) and is now centered at $(-d, 0)$. That is, eigenvalues of matrix $\mathbf{H}$ are contained in a line segment on the real axis with length $2\sigma\sqrt{2SC}$ and center $(-d, 0)$ (Supplemental Fig. S1). Therefore, the reactivity of random communities can be derived as

$$\mathcal{R} = -d + \sigma\sqrt{2SC}, \tag{5}$$

and the corresponding reactivity criterion is

$$\sigma\sqrt{2SC} > d. \tag{6}$$

1.2 Community with single interaction type

1.2.1 Exploitative community

From the construction algorithm described in the main text, the statistics of $\mathbf{M}$ of exploitative communities can be obtained as: $\mathbb{E}\left(M_{ij}\right)_{ij} = 0$, $\mathrm{Var}\left(M_{ij}\right)_{i\neq j} = C\sigma^2$ and $\mathbb{E}\left(M_{ij}M_{ji}\right)_{i\neq j} = -C\mathbb{E}^2\left(|Z|\right)$.

We first ignore the effect of diagonal elements by constructing matrix $\mathbf{M}' = \mathbf{M} + d\mathbf{I}$. For sufficiently large communities, the mean, variance and correlation of $\mathbf{M}'$ become $\mathbb{E}\left(M'_{ij}\right) = 0$, $\mathrm{Var}\left(M'_{ij}\right) = C\sigma^2$ and $\mathbb{E}\left(M'_{ij}M'_{ji}\right)_{i\neq j} = -C\mathbb{E}^2(|Z|)$. The fact that $\mathbf{H} = \left(\mathbf{M} + \mathbf{M}^{\mathrm{T}}\right)/2$ leads to $\mathbf{H}' = \left(\mathbf{M}' + \mathbf{M}'^{\mathrm{T}}\right)/2$, and we have

$$\begin{cases} \mathbb{E}\left(H'_{ij}\right) = \mathbb{E}\left(\frac{M'_{ij}+M'_{ji}}{2}\right) = 0, \\ \mathrm{Var}\left(H'_{ij}\right) = \mathbb{E}\left(H'^2_{ij}\right) - \mathbb{E}^2\left(H'_{ij}\right) = \mathbb{E}\left(H'^2_{ij}\right) = \frac{1}{2}\mathbb{E}\left(M'^2_{ij}\right) + \frac{1}{2}\mathbb{E}\left(M'_{ij}M'_{ji}\right)_{i\neq j}, \\ \mathbb{E}\left(H'_{ij}H'_{ji}\right)_{i\neq j} = \mathbb{E}\left(\left(\frac{M'_{ij}+M'_{ji}}{2}\right)^2\right) = \frac{1}{2}\mathbb{E}\left(M'^2_{ij}\right) + \frac{1}{2}\mathbb{E}\left(M'_{ij}M'_{ji}\right)_{i\neq j}. \end{cases} \tag{7}$$

Since $\mathrm{Var}\left(M_{ij}\right)_{i\neq j} = \mathbb{E}\left(M'^2_{ij}\right) = C\sigma^2$ and $\mathbb{E}\left(M'^2_{ij}\right) = C\mathbb{E}^2(|Z|)$, equations above become

$$\begin{cases} \mathbb{E}\left(H'_{ij}\right) = 0, \\ \mathrm{Var}\left(H'_{ij}\right) = \frac{1}{2}C\left(\sigma^2 - \mathbb{E}^2\left(|Z|\right)\right), \\ \mathbb{E}\left(H'_{ij}H'_{ji}\right)_{i\neq j} = \frac{1}{2}C\left(\sigma^2 - \mathbb{E}^2\left(|Z|\right)\right). \end{cases} \tag{8}$$

Then we consider matrix $\mathbf{N} = \mathbf{H}' \Big/ \sqrt{S \cdot \mathrm{Var}\left(H'_{ij}\right)}$, and have

$$\begin{cases} \mathbb{E}\left(N_{ij}\right) = 0, \\ \mathrm{Var}\left(N_{ij}\right) = \frac{1}{S}, \\ \mathbb{E}\left(N_{ij}N_{ji}\right)_{i \neq j} = \frac{1}{S}. \end{cases} \tag{9}$$

$\mathbf{N}$ now satisfies the elliptic law [1–4], and its eigenvalues are distributed in a line segment centered at $(0, 0)$ with length 4 on the real axis. This further brings the range of $\lambda_{\mathbf{H}'}$: eigenvalues of $\mathbf{H}'$ are contained in a line segment centered at $(0, 0)$ with length $2\sqrt{2SC\left(\sigma^2 - \mathbb{E}^2(|Z|)\right)}$ on the real axis.

We can now turn to matrix $\mathbf{H} = \mathbf{H}' - d\mathbf{I}$ to obtain the theoretical expression of reactivity. Since compared with $\mathbf{H}'$, eigenvalue distribution of $\mathbf{H}$ are shifted leftwards and are now centered at $(-d, 0)$, eigenvalues of $\mathbf{H}$ are now contained in a line segment centered at $(-d, 0)$ with length $2\sqrt{2SC\left(\sigma^2 - \mathbb{E}^2(|Z|)\right)}$ on the real axis (Supplemental Fig. S1), and the reactivity of exploitative communities can be derived as

$$\mathcal{R} = -d + \sqrt{2SC\left(\sigma^2 - \mathbb{E}^2(|Z|)\right)}. \tag{10}$$

The reactivity criterion for exploitative communities is then

$$\sqrt{2SC\left(\sigma^2 - \mathbb{E}^2(|Z|)\right)} > d. \tag{11}$$

1.2.2 Competitive community

From the construction algorithm, the statistics of $\mathbf{M}$ of competitive communities can be derived as: $\mathbb{E}\left(M_{ij}\right)_{i \neq j} = -C\mathbb{E}(|Z|)$, $\mathrm{Var}\left(M_{ij}\right)_{i \neq j} = C\sigma^2 - C^2\mathbb{E}^2(|Z|)$ and $\mathbb{E}\left(M_{ij}M_{ji}\right)_{i \neq j} = C\mathbb{E}^2(|Z|)$.

We still first ignore the effect of diagonal elements by considering matrix $\mathbf{M}' = \mathbf{M} + d\mathbf{I}$. When the community is sufficiently large, we have $\mathbb{E}\left(M'_{ij}\right) = -C\mathbb{E}(|Z|)$, $\mathrm{Var}\left(M'_{ij}\right) = C\sigma^2 - C^2\mathbb{E}^2(|Z|)$ and $\mathbb{E}\left(M'_{ij}M'_{ji}\right)_{i \neq j} = C\mathbb{E}^2(|Z|)$. Since $\mathbf{H} = \left(\mathbf{M} + \mathbf{M}^{\mathrm{T}}\right)/2$ and $\mathbf{H}' = \left(\mathbf{M}' + \mathbf{M}'^{\mathrm{T}}\right)/2$, we have

$$\begin{cases} \mathbb{E}\left(H'_{ij}\right) = \mathbb{E}\left(\frac{M'_{ij} + M'_{ji}}{2}\right) = -C\mathbb{E}(|Z|), \\ \mathrm{Var}\left(H'_{ij}\right) = \mathbb{E}\left(H'^2_{ij}\right) - \mathbb{E}^2\left(H'_{ij}\right) = \frac{1}{2}\mathbb{E}\left(M'^2_{ij}\right) + \frac{1}{2}\mathbb{E}\left(M'_{ij}M'_{ji}\right)_{i \neq j} - \mathbb{E}^2\left(M'_{ij}\right), \\ \mathbb{E}\left(H'_{ij}H'_{ji}\right)_{i \neq j} = \mathbb{E}\left(\left(\frac{M'_{ij} + M'_{ji}}{2}\right)^2\right) = \frac{1}{2}\mathbb{E}\left(M'^2_{ij}\right) + \frac{1}{2}\mathbb{E}\left(M'_{ij}M'_{ji}\right)_{i \neq j}. \end{cases} \tag{12}$$

Since $\mathbb{E}\left(M'^2_{ij}\right) = C\sigma^2$, equations above can be rewritten as

$$\begin{cases} \mathbb{E}\left(H'_{ij}\right) = -C\mathbb{E}(|Z|), \\ \mathrm{Var}\left(H'_{ij}\right) = \frac{1}{2}C\left(\sigma^2 + \mathbb{E}^2(|Z|) - 2C\mathbb{E}^2(|Z|)\right), \\ \mathbb{E}\left(H'_{ij}H'_{ji}\right)_{i\neq j} = \frac{1}{2}C\left(\sigma^2 + \mathbb{E}^2(|Z|)\right). \end{cases} \tag{13}$$

Since $\mathbb{E}\left(H'_{ij}\right) \neq 0$, we can not follow the procedures used in random case and exploitative case to rescale matrix $\mathbf{H'}$ and directly apply the elliptic law. To deal with this problem, we let $E = -C\mathbb{E}(|Z|)$ and rewrite $\mathbf{H'}$ as

$$\begin{aligned} \mathbf{H'} &= \left(\mathbf{H'} - E\cdot\mathbf{1}\cdot\mathbf{1}^{\mathrm{T}} + E\mathbf{I}\right) + E\cdot\mathbf{1}\cdot\mathbf{1}^{\mathrm{T}} - E\mathbf{I} \\ &= \mathbf{N} + E\cdot\mathbf{1}\cdot\mathbf{1}^{\mathrm{T}} - E\mathbf{I}, \end{aligned} \tag{14}$$

where $\mathbf{1}\cdot\mathbf{1}^{\mathrm{T}}$ is an $S\times S$ matrix with each entry equaling 1. We can then have $N_{ij,i\neq j} = H'_{ij,i\neq j} - E$, $N_{ii} = H'_{ii} = 0$. The corresponding statistics of $\mathbf{N}$ are now

$$\begin{cases} \mathbb{E}\left(N_{ij}\right) = 0, \\ \mathrm{Var}\left(N_{ij}\right) = \mathrm{Var}\left(H'_{ij}\right) = \frac{1}{2}C\left(\sigma^2 + \mathbb{E}^2(|Z|) - 2C\mathbb{E}^2(|Z|)\right), \\ \mathbb{E}\left(N_{ij}N_{ji}\right)_{i\neq j} = \frac{1}{2}C\left(\sigma^2 + \mathbb{E}^2(|Z|) - 2C\mathbb{E}^2(|Z|)\right). \end{cases} \tag{15}$$

Obviously, $\mathbf{N}$ is an elliptic matrix. We then construct matrix $\mathbf{N}^* = \mathbf{N}\big/\sqrt{S\cdot\mathrm{Var}\left(N_{ij}\right)}$, and have

$$\begin{cases} \mathbb{E}\left(N^*_{ij}\right) = 0, \\ \mathrm{Var}\left(N^*_{ij}\right) = \mathrm{Var}\left(H_{ij}\right) = \frac{1}{S}, \\ \mathbb{E}\left(N^*_{ij}N^*_{ji}\right)_{i\neq j} = \frac{1}{S}. \end{cases} \tag{16}$$

$\mathbf{N}^*$ is now a standard elliptic matrix, and its eigenvalues are distributed in a line segment centered at $(0,0)$ with length equals 4 on the real axis. Thus, eigenvalues of $\mathbf{N}$ are contained in a line segment centered at $(0,0)$ with length $2\sqrt{2\sigma^2SC + 2SC\mathbb{E}^2(|Z|)(1-2C)}$ on the real axis.

Then, we turn to matrix $\mathbf{H'} = \mathbf{N} + E\cdot\mathbf{1}\cdot\mathbf{1}^{\mathrm{T}} - E\mathbf{I}$. $E\cdot\mathbf{1}\cdot\mathbf{1}^{\mathrm{T}}$ term is a rank-one perturbation: $S-1$ of its eigenvalues are zero, and a single eigenvalue is equal to SE. According to the low-rank perturbations theorem [3, 5], when $|E| \leq \sqrt{\mathrm{Var}\left(N_{ij}\right)/S}$, the eigenvalues of $\mathbf{N} + E\cdot\mathbf{1}\cdot\mathbf{1}^{\mathrm{T}}$ are still distributed in a line segment centered at $(0,0)$ with length $2\sqrt{2\sigma^2SC + 2SC\mathbb{E}^2\left(|Z|\right)\left(1-2C\right)}$ on the real axis. However, when $|E| > \sqrt{\mathrm{Var}\left(N_{ij}\right)/S}$, $S-1$ eigenvalues of $\mathbf{N} + E\cdot\mathbf{1}\cdot\mathbf{1}^{\mathrm{T}}$ are still in the

line segment stated above, while a single eigenvalue will be modified and approximately be equal to $SE + \mathrm{Var}(N_{ij})/E$. When S is sufficiently large, this single eigenvalue can be further approximated as $SE = -SC\mathbb{E}(|Z|)$. Finally, the effect of $-E\mathbf{I}$ is to shift the whole distribution by subtracting E from each eigenvalue. Since $|E| > \sqrt{\mathrm{Var}(N_{ij})/S}$ can hold when S is sufficiently large, the eigenvalue distribution for sufficiently large $\mathbf{H}'$ can be obtained as: a single eigenvalue is approximated as $-(S-1)C\mathbb{E}(|Z|)$, and $S-1$ eigenvalues are contained in a line segment centered at $(C\mathbb{E}(|Z|), 0)$ with length $2\sqrt{2\sigma^2 SC + 2SC\mathbb{E}^2(|Z|)(1-2C)}$ on the real axis.

Compared with the eigenvalues of matrix $\mathbf{H}'$, the eigenvalues of matrix $\mathbf{H}$ are shifted leftwards and are now centered at $(-d, 0)$. Thus, the eigenvalue distribution for sufficiently large $\mathbf{H}$ is: a single eigenvalue is approximated as $-d-(S-1)C\mathbb{E}(|Z|)$ (or more precisely, this single eigenvalue can be approximated as $-d-(S-1)C\mathbb{E}(|Z|)-\left(\sigma^2+\mathbb{E}^2(|Z|)(1-2C)\right)/(2\mathbb{E}(|Z|))$), and $S-1$ eigenvalues are contained in a line segment centered at $(-d + C\mathbb{E}(|Z|), 0)$ with length $2\sqrt{2\sigma^2 SC + 2SC\mathbb{E}^2(|Z|)(1-2C)}$ (Supplemental Fig. S1). Since the outlier is on the left side of the line segment and thus, the reactivity of competitive communities is

$$\mathcal{R} = -d + C\mathbb{E}(|Z|) + \sqrt{2\sigma^2 SC + 2SC\mathbb{E}^2(|Z|)(1-2C)}, \tag{17}$$

and the corresponding reactivity criterion is

$$C\mathbb{E}(|Z|) + \sqrt{2\sigma^2 SC + 2SC\mathbb{E}^2(|Z|)(1-2C)} > d. \tag{18}$$

1.2.3 Mutualistic community

We still first consider the matrix $\mathbf{M}' = \mathbf{M} + d\mathbf{I}$. It is clear that $\mathbf{M}'_{\text{mutualistic}}$ is equivalent to $-\mathbf{M}'_{\text{competitive}}$, which further leads to the fact that $\mathbf{H}'_{\text{mutualistic}}$ and $-\mathbf{H}'_{\text{competitive}}$ are equivalent. Thus, the eigenvalue distribution for sufficiently large $\mathbf{H}$ of mutualistic communities can be obtained: a single eigenvalue is approximated as $-d + (S-1)C\mathbb{E}(|Z|)$ (or more precisely, $-d + (S-1)C\mathbb{E}(|Z|) + \left(\sigma^2+\mathbb{E}^2(|Z|)(1-2C)\right)/(2\mathbb{E}(|Z|))$), and $S-1$ eigenvalues are contained in a line segment centered at $(-d - C\mathbb{E}(|Z|), 0)$ with length $2\sqrt{2\sigma^2 SC + 2SC\mathbb{E}^2(|Z|)(1-2C)}$ (Supplemental Fig. S1). In mutualistic case, the outlier is on the right side of the bulk eigenvalues, and the reactivity is determined by this outlier

$$\mathcal{R} = -d + (S-1)C\mathbb{E}(|Z|). \tag{19}$$

The corresponding reactivity criterion is then

$$(S-1)\,C\mathbb{E}\left(|Z|\right) > d. \tag{20}$$

Note that when S is not so large (i.e., $|E| \leq \sqrt{\mathrm{Var}\left(N_{ij}\right)/S}$), the outlier does not exist, and the reactivity can be obtained as

$$\mathcal{R} = -d - C\mathbb{E}\left(|Z|\right) + \sqrt{2\sigma^2 SC + 2SC\mathbb{E}^2\left(|Z|\right)(1-2C)}. \tag{21}$$

1.3 Community with mixed types of interactions

Previous parts deal with communities with single interaction type. In this part, we extend the theory to communities with mixed interaction types. We still first ignore the influence of diagonal elements, i.e., consider matrix $\mathbf{M}' = \mathbf{M} + d\mathbf{I}$. The statistics for sufficiently large matrix $\mathbf{M}'$ can be drawn according to the construction algorithm as

$$\begin{cases} \mathbb{E}\left(M'_{ij}\right) = C\mathbb{E}(|Z|)\left(P_{+/+} - P_{\text{-/-}}\right) \equiv E, \\ \mathrm{Var}\left(M'_{ij}\right) = \mathbb{E}\left(M'^2_{ij}\right) - \mathbb{E}^2\left(M'_{ij}\right) = C\sigma^2 - E^2 \equiv V, \\ \mathbb{E}\left(M'_{ij}M'_{ji}\right)_{i\neq j} = C\mathbb{E}^2(|Z|)\left(P_{+/+} + P_{\text{-/-}} - P_{\text{+/-}}\right) \equiv \rho. \end{cases} \tag{22}$$

Since $\mathbf{H}' = \left(\mathbf{M}' + \mathbf{M}'^{\mathrm{T}}\right)/2$, the statistics of $\mathbf{H}'$ are

$$\begin{cases} \mathbb{E}\left(H'_{ij}\right) = \mathbb{E}\left(\frac{M'_{ij}+M'_{ji}}{2}\right) = \mathbb{E}\left(M'_{ij}\right), \\ \mathrm{Var}\left(H'_{ij}\right) = \mathbb{E}\left(H'^2_{ij}\right) - \mathbb{E}^2\left(H'_{ij}\right) = \frac{1}{2}\mathbb{E}\left(M'^2_{ij}\right) + \frac{1}{2}\mathbb{E}\left(M'_{ij}M'_{ji}\right)_{i\neq j} - E^2, \\ \mathbb{E}\left(H'_{ij}H'_{ji}\right)_{i\neq j} = \mathbb{E}\left(H'^2_{ij}\right) = \frac{1}{2}\mathbb{E}\left(M'^2_{ij}\right) + \frac{1}{2}\mathbb{E}\left(M'_{ij}M'_{ji}\right)_{i\neq j}, \end{cases} \tag{23}$$

which are equivalent to

$$\begin{cases} \mathbb{E}\left(H'_{ij}\right) = C\mathbb{E}(|Z|)\left(P_{+/+} - P_{\text{-/-}}\right), \\ \mathrm{Var}\left(H'_{ij}\right) = \frac{1}{2}C\sigma^2 + \frac{1}{2}C\mathbb{E}^2(|Z|)(P_{+/+} + P_{\text{-/-}} - P_{\text{+/-}}) - E^2, \\ \mathbb{E}\left(H'_{ij}H'_{ji}\right)_{i\neq j} = \frac{1}{2}C\sigma^2 + \frac{1}{2}C\mathbb{E}^2(|Z|)\left(P_{+/+} + P_{\text{-/-}} - P_{\text{+/-}}\right). \end{cases} \tag{24}$$

These equations can further be represented as

$$\begin{cases} \mathbb{E}\left(H'_{ij}\right) = E, \\ \mathrm{Var}\left(H'_{ij}\right) = \frac{1}{2}\left(V + \rho - E^2\right), \\ \mathbb{E}\left(H'_{ij}H'_{ji}\right)_{i\neq j} = \frac{1}{2}\left(V + \rho + E^2\right). \end{cases} \tag{25}$$

Following the procedures used in the competitive case, we rewrite $\mathbf{H}'$ as

$$\begin{aligned} \mathbf{H}' &= \left(\mathbf{H}' - E \cdot \mathbf{1} \cdot \mathbf{1}^{\mathrm{T}} + E\mathbf{I}\right) + E \cdot \mathbf{1} \cdot \mathbf{1}^{\mathrm{T}} - E\mathbf{I} \\ &= \mathbf{N} + E \cdot \mathbf{1} \cdot \mathbf{1}^{\mathrm{T}} - E\mathbf{I}, \end{aligned} \tag{26}$$

where $\mathbf{1} \cdot \mathbf{1}^{\mathrm{T}}$ is an $S \times S$ matrix with each entry equaling 1. The statistics of $\mathbf{N}$ are

$$\begin{cases} \mathbb{E}\left(N_{ij}\right) = 0, \\ \mathrm{Var}\left(N_{ij}\right) = \mathrm{Var}(H'_{ij}) = \frac{1}{2}\left(V + \rho - E^2\right), \\ \mathbb{E}\left(N_{ij}N_{ji}\right)_{i\neq j} = \frac{1}{2}\left(V + \rho - E^2\right). \end{cases} \tag{27}$$

Then consider matrix $\mathbf{N}^* = \mathbf{N} \left/ \sqrt{S \cdot \mathrm{Var}(N_{ij})}\right.$, we have

$$\begin{cases} \mathbb{E}\left(N^*_{ij}\right) = 0, \\ \mathrm{Var}\left(N^*_{ij}\right) = \frac{1}{S}, \\ \mathbb{E}\left(N^*_{ij}N^*_{ji}\right)_{i\neq j} = \frac{1}{S}. \end{cases} \tag{28}$$

$\mathbf{N}^*$ now is a standard elliptic matrix, and its eigenvalues are contained in a line segment on the real axis whose location and length are determined by the elliptic law [1–4]. The eigenvalue distribution of $\mathbf{N}$ can then be derived: eigenvalues of $\mathbf{N}$ are contained in a line segment centered at $(0, 0)$ with length $4\sqrt{S\mathrm{Var}\left(N_{ij}\right)}$.

According to the low-rank perturbations theorem [3,5], we can obtain the eigenvalue distribution of matrix $\mathbf{H}'$ when the community is sufficiently large. When $|E| \leq \sqrt{\left(\mathrm{V} + \rho - E^2\right)/\left(2S\right)}$, eigenvalues of $\mathbf{H}'$ are contained in a line segment centered at $(-E, 0)$ with length $4\sqrt{S\mathrm{Var}\left(N_{ij}\right)}$ on the real axis. When $|E| > \sqrt{\left(\mathrm{V} + \rho - E^2\right)/\left(2S\right)}$, a single eigenvalue is approximated as $(S-1)\,E$ (or more precisely, $(S-1)\,E + \left(V + \rho - E^2\right)/\left(2E\right)$), and $S - 1$ eigenvalues are contained in a line segment centered at $(-E, 0)$ with length $4\sqrt{S\mathrm{Var}\left(N_{ij}\right)}$ on the real axis.

We then turn to matrix $\mathbf{H} = \mathbf{H}' - d\mathbf{I}$. When $|E| \leq \sqrt{\left(\mathrm{V} + \rho - E^2\right)/\left(2S\right)}$, eigenvalues of $\mathbf{H}'$ are contained in a line segment centered at $(-d - E, 0)$ with length $4\sqrt{S\mathrm{Var}\left(N_{ij}\right)}$ on the real axis. When

$|E| > \sqrt{(\mathrm{V} + \rho - E^2)\,/\,(2S)}$, a single eigenvalue is approximated as $-d+(S-1)\,E$ (or more precisely, $-d+(S-1)\,E+\left(V+\rho-E^2\right)/\,(2E)$), and $S-1$ eigenvalues are contained in a line segment centered at $(-d-E, 0)$ with length $4\sqrt{S\mathrm{Var}\,(N_{ij})}$ on the real axis.

Accordingly, we can obtain the reactivity of sufficiently large and complex communities with mixed types of interactions

$$\begin{cases} \mathcal{R} = -d - E + \sqrt{2S(\mathrm{V} + \rho - E^2)}, \ |E| \le \sqrt{\frac{V+\rho-E^2}{2S}}, \\ \mathcal{R} = \max\left(-d + (S-1)\,E, -d - E + \sqrt{2S(\mathrm{V} + \rho - E^2)}\right), \ |E| > \sqrt{\frac{V+\rho-E^2}{2S}}. \end{cases} \tag{29}$$

It is clear from Supplemental Fig. S2 that the increase of the proportion of competitive interactions and mutualistic interactions makes the community more reactive.

The corresponding reactivity criterion for sufficiently large and complex communities with mixed types of interactions can also be derived

$$\begin{cases} -E + \sqrt{2S(\mathrm{V} + \rho - E^2)} > d, \ |E| \le \sqrt{\frac{V+\rho-E^2}{2S}}, \\ \max\left(-d + (S-1)\,E, -d - E + \sqrt{2S(\mathrm{V} + \rho - E^2)}\right) > 0, \ |E| > \sqrt{\frac{V+\rho-E^2}{2S}}. \end{cases} \tag{30}$$

2 Supplementary Note 2: The influence of weak interactions on system reactivity

In the main text, we give a simple analysis to evaluate the effect of weak interactions on system reactivity. Since the analytical expressions derived in Supplementary Note 1 hold for arbitrary distributions of interaction strength, here we show the correctness of conclusions drawn in the main text by first comparing the reactivity criteria under normal distribution and uniform distribution. Then, we derive the corresponding reactivity criteria under Gamma distribution to further explore this question.

2.1 Comparison between normal distribution & uniform distribution

Suppose interaction strengths are sampled from a normal distribution $Z \sim N\left(0, \sigma^2\right)$ and a uniform distribution $Z \sim U\left[-\sqrt{3}\sigma, \sqrt{3}\sigma\right]$ respectively. These two distributions yield the same mean and variance: $\mathbb{E}\left(Z\right) = 0$ and $\mathrm{Var}\left(Z\right) = \sigma^2$. In the case of normal distribution, we have $\mathbb{E}(|Z|) = \left(\sqrt{2/\pi}\right)\sigma \approx 0.798\sigma$, while in the case of uniform distribution, we have $\mathbb{E}(|Z|) = \left(\sqrt{3}/2\right)\sigma \approx 0.866\sigma$ ($\mathbb{E}(|Z|)$ is the expected magnitude of interaction strength). This means that, on average, sampling from uniform distribution brings less weak interactions than sampling from normal distribution. We then check the reactivity of each type of communities under these two distributions.

Random community. Since the reactivity criterion for random community is $\sigma\sqrt{2SC} > d$, which doesn't contain $\mathbb{E}(|Z|)$, it is not affected by the exact form of interaction strength distribution (Supplemental Fig. S3).

Exploitative community. For exploitative communities, the reactivity criterion under normal distribution becomes $\sigma\sqrt{0.7264SC} > d$, while the reactivity criterion under uniform distribution is $\sigma\sqrt{0.5SC} > d$. The depiction of reactivity criterion (Supplemental Fig. S3) shows clearly that compared with its uniform distribution counterpart, exploitative community under normal distribution is more likely to be reactive.

Competitive community. In the case of normal distribution, the reactivity criterion is $0.7979\sigma C + \sqrt{\sigma^2 SC(3.2732 - 2.5465C)} > d$. In the case of uniform distribution, the reactivity becomes $0.866\sigma C + \sqrt{\sigma^2 SC(3.5 - 3C)} > d$. The depiction of reactivity criterion (Supplemental Fig. S3) shows clearly that compared with its uniform distribution counterpart, competitive community under normal distribution is less likely to be reactive.

Mutualistic community. Under normal distribution, the reactivity criterion is $0.7979\sigma(S -$

$1)C > d$. Under uniform distribution, the reactivity criterion becomes $0.866\sigma(S-1)C > d$. Obviously, mutualistic community under normal distribution is less likely to be reactive (Supplemental Fig. S3).

Analysis above proves the argument we state in the main text, i.e., prevalence of weak interactions make exploitative communities more reactive, have no effect on random communities, make competitive communities and mutualistic communities less reactive.

2.2 Reactivity criteria under Gamma distribution

We choose a symmetric distribution for the random variable Z, from which interspecies interaction strength takes value:

$$\begin{cases} Z|Z>0,\ Z\sim\Gamma(k,\sigma\sqrt{\frac{1}{k^2+k}}),\ k>0, \\ Z|Z<0,\ Z\sim-\Gamma(k,\sigma\sqrt{\frac{1}{k^2+k}}),\ k>0. \end{cases} \tag{31}$$

For arbitrary choice of k, $\mathbb{E}(Z)=0$ and $\mathrm{Var}(Z)=\sigma^2$ always hold. We can now adjust the proportion of weak interactions by changing the value of k. The corresponding expected magnitude of interaction strength can be calcuated as: $\mathbb{E}(|Z|)=k\sigma\sqrt{\frac{1}{k^2+k}}=\sigma\sqrt{\frac{k}{k+1}}$. Thus, large k leads to $\mathbb{E}(|Z|)\approx\sigma$, meaning that weak interactions are rare. Small k leads to $\mathbb{E}(|Z|)\approx 0$, meaning that weak interactions are preponderant (Supplemental Fig. S4).

The analytical expressions of reactivity of different types of communities under this Gamma distribution can be drawn as follows:

Random community: The reactivity of random communities is $\mathcal{R}=-d+\sigma\sqrt{2SC}$, meaning that prevalence of weak interactions has no effect on random communities (Supplemental Fig. S4).

Exploitative community: The reactivity of exploitative communities is

$$\mathcal{R}=-d+\sigma\sqrt{\frac{2SC}{k+1}}, \tag{32}$$

which leads to

$$\frac{\mathrm{d}\mathcal{R}}{\mathrm{d}k}=-\frac{\sigma SC}{(k+1)^2\sqrt{\frac{2SC}{k+1}}}<0. \tag{33}$$

Clearly, small k makes exploitative systems more reactive, and this leads to the conclusion that the prevalence of weak interactions makes exploitative systems more reactive (Supplemental Fig. S4).

Competitive community: The reactivity of competitive communities is

$$\mathcal{R}=-d+\sigma C\sqrt{\frac{k}{k+1}}+\sigma\sqrt{2SC\frac{2k(1-C)+1}{k+1}}, \tag{34}$$

which brings

$$\frac{\mathrm{d}\mathcal{R}}{\mathrm{d}k} = \frac{\sigma C}{2(k+1)^2\sqrt{\frac{k}{k+1}}} + \frac{\sigma SC(1-2C)}{(k+1)^2\sqrt{\frac{2SC(2k-2kC+1)}{k+1}}}. \tag{35}$$

When $C < 0.5$, equation above is always larger than 0, indicating that large k makes the competitive community more reactive. As for cases when $C > 0.5$, the results are more complicated. However, since we are interested in sufficiently large and stable competitive communities, the corresponding connectance should be relatively small, usually less than 0.5 (e.g., for a competitive community with parameter $d = 1, S = 100$ and $\sigma = 0.15$, the system becomes unstable when $C > 0.2$). We can then in general conclude that large k makes a stable competitive community more reactive (Supplemental Fig. S4).

Mutualistic community: For sufficiently large mutualistic community, the reactivity is $\mathcal{R} = -d + \sigma(S-1)C\sqrt{k/\left(k+1\right)}$, and we have

$$\frac{\mathrm{d}\mathcal{R}}{\mathrm{d}k} = \frac{\sigma(S-1)C}{2(k+1)^2\sqrt{\frac{k}{k+1}}} > 0 \tag{36}$$

Obviously, large k leads to a more reactive community (Supplemental Fig. S4).

Reactivity analysis under Gamma distribution again confirms our previous statement — prevalence of weak interactions make exploitative communities more reactive, have no effect on random communities, make competitive communities and mutualistic communities less reactive.

3 Supplementary Note 3: Measuring the distance between reactivity and instability

3.1 Random community

For random communities, the stability criterion is $\sigma\sqrt{SC} < d$ [1, 6], and the critical $S - C$ curve is described by

$$S = \frac{d^2}{\sigma^2}\frac{1}{C}. \tag{37}$$

The area of stable region in the $S - C$ plane when $C \in [C_1, C_2]$ is

$$A_{\text{stable, random}} = \frac{d^2}{\sigma^2}\ln\left(\frac{C_2}{C_1}\right). \tag{38}$$

The reactivity criterion is $\sigma\sqrt{2SC} > d$, and the corresponding critical $S - C$ curve is

$$S = \frac{d^2}{2\sigma^2}\frac{1}{C}. \tag{39}$$

The area of non-reactive region in the $S - C$ plane when $C \in [C_1, C2]$ is

$$A_{\text{non-reactive,random}} = \frac{d^2}{2\sigma^2}\ln\left(\frac{C_2}{C_1}\right). \tag{40}$$

Thus, the normalised distance (ND) between reactivity and instability is

$$\text{ND}_{\text{random}} = \frac{A_{\text{reactive stable, random}}}{A_{\text{stable, random}}} = 1 - \frac{A_{\text{non-reactive, random}}}{A_{\text{stable, random}}} = \frac{1}{2}. \tag{41}$$

When S or C is fixed, the area ratio can be replaced by critical S ratio or critical C ratio. Here we give an example of fixed C (i.e., $C = C_1$). The critical S for instability can be obtained as

$$S_{\text{stable,random}} = \frac{d^2}{\sigma^2}\frac{1}{C_1}, \tag{42}$$

and the critical S for reactivity is

$$S_{\text{reactive,random}} = \frac{d^2}{2\sigma^2}\frac{1}{C_1}. \tag{43}$$

The normalised distance then becomes

$$\mathrm{ND}_{\text{random}} = \frac{S_{\text{reactive stable,random}}}{S_{\text{stable,random}}} = 1 - \frac{S_{\text{non-reactive, random}}}{S_{\text{stable, random}}} = \frac{1}{2}. \tag{44}$$

3.2 Exploitative community

The stability criterion of exploitative communities is $\sigma\sqrt{SC}\left(1 - \mathbb{E}^2(|Z|)/\sigma^2\right) < d$ [1], and the corresponding critical $S - C$ curve becomes

$$S = \left(\frac{\sigma d}{\sigma^2 - \mathbb{E}^2(|Z|)}\right)^2 \frac{1}{C}. \tag{45}$$

When $C \in [C_1, C_2]$, the area of stable region can be calculated as

$$A_{\text{stable,exploitative}} = \left(\frac{\sigma d}{\sigma^2 - \mathbb{E}^2(|Z|)}\right)^2 \ln\left(\frac{C_2}{C_1}\right). \tag{46}$$

The reactivity criterion of exploitative communities is $\sqrt{2SC[\sigma^2 - \mathbb{E}^2(|Z|)]} > d$, leading to the critical $S - C$ curve

$$S = \frac{d^2}{2\left(\sigma^2 - \mathbb{E}^2(|Z|)\right)} \frac{1}{C}. \tag{47}$$

The area of non-reactive region is then

$$A_{\text{non-reactive,exploitative}} = \frac{d^2}{2\left(\sigma^2 - \mathbb{E}^2(|Z|)\right)} \ln\left(\frac{C_2}{C_1}\right). \tag{48}$$

The normalised distance between reactivity and instability is then

$$\begin{aligned} \mathrm{ND}_{\text{exploitative}} &= \frac{A_{\text{reactive stable, exploitative}}}{A_{\text{stable, exploitative}}} = 1 - \frac{A_{\text{non-reactive, exploitative}}}{A_{\text{stable, exploitative}}} \\ &= 1 - \frac{\sigma^2 - \mathbb{E}^2(|Z|)}{2\sigma^2} > \frac{1}{2}. \end{aligned} \tag{49}$$

Therefore, the normalised distance between reactivity and instability of exploitative communities is larger than that of random community communities.

We also give an example when C is fixed to C_1. In this case, the critical S for stability and reactivity are

$$S_{\text{stable,exploitative}} = \left(\frac{\sigma d}{\sigma^2 - \mathbb{E}^2(|Z|)}\right)^2 \frac{1}{C_1}, \tag{50}$$

and

$$S_{\text{reactive,exploitative}} = \frac{d^2}{2\left(\sigma^2 - \mathbb{E}^2(|Z|)\right)} \frac{1}{C_1} \tag{51}$$

respectively. The normalised distance is then

$$\begin{aligned} \text{ND}_{\text{exploitative}} &= \frac{S_{\text{reactive stable, exploitative}}}{S_{\text{stable, exploitative}}} = 1 - \frac{S_{\text{non-reactive, exploitative}}}{S_{\text{stable, exploitative}}} \\ &= 1 - \frac{\sigma^2 - \mathbb{E}^2(|Z|)}{2\sigma^2} > \frac{1}{2}. \end{aligned} \tag{52}$$

3.3 Competitive community

For competitive communities, the stability criterion [1] and reactivity criterion are

$$C\mathbb{E}(|Z|) + \sqrt{SC}\frac{\sigma^2 + (1-2C)\mathbb{E}^2(|Z|)}{\sqrt{\sigma^2 - C\mathbb{E}^2(|Z|)}} < d \tag{53}$$

and

$$C\mathbb{E}(|Z|) + \sqrt{2\sigma^2 SC + 2SC\mathbb{E}^2(|Z|)(1-2C)} > d \tag{54}$$

respectively. Therefore, the critical $S - C$ curves for stability and reactivity are

$$S = \left(d - C\mathbb{E}(|Z|)\right)^2 \frac{\sigma^2 - C\mathbb{E}^2(|Z|)}{\left(\sigma^2 + (1-2C)\,\mathbb{E}^2\left(|Z|\right)\right)^2} \frac{1}{C}, \tag{55}$$

and

$$S = \frac{\left(d - C\mathbb{E}(|Z|)\right)^2}{2\left(\sigma^2 + (1-2C)\,\mathbb{E}^2\left(|Z|\right)\right)} \frac{1}{C}. \tag{56}$$

We can't get exact analytical expressions for the area of stable region and non-reactive region, and thus the normalised distance between reactivity and instability. However, we can get a higher bound for this distance. For the critical $S - C$ curve of stability, following inequality holds

$$\begin{aligned} S &= \left(d - C\mathbb{E}(|Z|)\right)^2 \frac{\sigma^2 - C\mathbb{E}^2(|Z|)}{\left(\sigma^2 + (1-2C)\mathbb{E}^2(|Z|)\right)^2} \frac{1}{C} \\ &= \frac{\left(d - C\mathbb{E}(|Z|)\right)^2}{\sigma^2 + (1-2C)\mathbb{E}^2\left(|Z|\right)} \frac{\sigma^2 - C\mathbb{E}^2\left(|Z|\right)}{\sigma^2 + (1-2C)\,\mathbb{E}^2(|Z|)} \frac{1}{C} \\ &\leq \frac{\left(d - C\mathbb{E}(|Z|)\right)^2}{\sigma^2 + (1-2C)\,\mathbb{E}^2(|Z|)} \frac{1}{C}, \end{aligned} \tag{57}$$

meaning that we have

$$A_{\text{stable,competitive}} < 2A_{\text{non-reactive,competitive}}. \tag{58}$$

when $C \in [C_1, C_2]$. Thus, the normalised distance between reactivity and instability satisfies

$$\mathrm{ND}_{\text{competitive}} = \frac{A_{\text{reactive stable, random}}}{A_{\text{stable, random}}} = 1 - \frac{A_{\text{non-reactive, random}}}{A_{\text{stable, random}}} \leq \frac{1}{2}, \tag{59}$$

which is higher than random case.

We also show the case when C is fixed to C_1. The critical S for stability and reactivity are then

$$S = (d - C_1\mathbb{E}(|Z|))^2 \frac{\sigma^2 - C_1\mathbb{E}^2(|Z|)}{(\sigma^2 + (1-2C_1)\mathbb{E}^2(|Z|))^2} \frac{1}{C_1}, \tag{60}$$

and

$$S = \frac{(d - C_1\mathbb{E}(|Z|))^2}{2(\sigma^2 + (1-2C_1)\mathbb{E}^2(|Z|))} \frac{1}{C_1}. \tag{61}$$

Similiarly, we can prove that

$$\begin{aligned} \mathrm{ND}_{\text{competitive}} &= \frac{S_{\text{reactive stable, random}}}{S_{\text{stable, random}}} = 1 - \frac{S_{\text{non-reactive, random}}}{S_{\text{stable, random}}} \\ &= 1 - \frac{\sigma^2 + (1-2C_1)\,\mathbb{E}^2(|Z|)}{2\,(\sigma^2 - C_1\mathbb{E}^2(|Z|))} \leq \frac{1}{2}. \end{aligned} \tag{62}$$

It is worth noticing that unlike random case and exploitative case, this normalised distance is not a constant: as C increases, the distance increases

$$\frac{\mathrm{dND}}{\mathrm{d}C_1} = \frac{\mathbb{E}^2(|Z|)(\sigma^2 - \mathbb{E}^2(|Z|))}{2(C\mathbb{E}^2(|Z|) - \sigma^2)^2} > 0, \tag{63}$$

and when $C_1 = 1$, the normalised distance reaches its maximum (i.e., 1/2).

3.4 Mutualistic community

For sufficiently large mutualistic communities, the stability criterion and reactivity criterion are $(S-1)C\mathbb{E}(|Z|) < d$ and $(S-1)C\mathbb{E}(|Z|) > d$, leading to the same critical $S-C$ curve. Thus, it is clear that for mutualistic communities, the normalised distance between reactivity and instability is 0, i.e., a sufficiently large reactive mutualistic community is always unstable.

3.5 Community with mixed types of interactions

With the extended theory for the reactivity of community with mixed types of interactions, we are also able to assess the effect of different interaction types on the normalised distance between reactivity and instability.

Here we recall the statistics of off-diagonal elements of the community matrix

$$\begin{cases} \mathbb{E}\left(M_{ij}\right)_{i\neq j} = C\mathbb{E}(|Z|)\left(P_{+/+} - P_{\text{-/-}}\right) = E, \\ \text{Var}\left(M_{ij}\right)_{i\neq j} = C\sigma^2 - E^2 = V, \\ \mathbb{E}\left(M_{ij}M_{ji}\right)_{i\neq j} = C\mathbb{E}^2(|Z|)\left(P_{+/+} + P_{\text{-/-}} - P_{+/\text{-}}\right) = \rho. \end{cases} \tag{64}$$

For sufficiently large communities, when stability and reactivity are both determined by the outlier (i.e., mutualistic interactions are preponderant), the stability criterion and reactivity criterion are $(S-1)\,E < d$ and $(S-1)\,E > d$ respectively. In this case, the critical $S - C$ curves for stability and reactivity are the same, meaning that a reactive community is always unstable. When stability and reactivity are not determined by the outlier, the stability criterion and reactivity criterion are

$$-E + \sqrt{S\text{V}}\left(1 + \frac{\rho - E^2}{\text{V}}\right) < d, \tag{65}$$

and

$$-E + \sqrt{2S\left(\text{V} + \rho - E^2\right)} > d \tag{66}$$

respectively. The critical $S - C$ curves are then

$$S = \frac{V(d+E)^2}{\left(V + \rho - E^2\right)^2}, \tag{67}$$

and

$$S = \frac{(d+E)^2}{2\left(V + \rho - E^2\right)}. \tag{68}$$

Thus, the normalised distance between reactivity and instability is

$$\text{ND}_{\text{mixed}} = 1 - \frac{1 + \frac{\rho - E^2}{V}}{2} \tag{69}$$

Please note that here the distance is obtained by fixing system connectance. Since the correlation coefficient $r\left(M_{ij}, M_{ji}\right)_{i\neq j}$ of matrix $\mathbf{M}$ is

$$r(M_{ij}, M_{ji})_{i\neq j} = \frac{\text{Cov}\left(M_{ij}, M_{ji}\right)_{i\neq j}}{\sqrt{\text{Var}\left(M_{ij}\right)_{i\neq j}\text{Var}\left(M_{ji}\right)_{i\neq j}}} = \frac{\rho - E^2}{V}, \tag{70}$$

and $r(M_{ij}, M_{ji})_{i\neq j} \in [-1, 1]$. The normalised distance can be rewritten as

$$\mathrm{ND}_{\text{mixed}} = 1 - \frac{1 + r\left(M_{ij}, M_{ji}\right)_{i\neq j}}{2} \tag{71}$$

It is clear that a postive $r\left(M_{ij}, M_{ji}\right)_{i\neq j}$ (i.e., competitive and mutualistic interactions are preponderant) brings a relatively low normalised distance (lower than 0.5) while a negative $r\left(M_{ij}, M_{ji}\right)_{i\neq j}$ (i.e., exploitative interactions are preponderant) brings a relatively high normalised distance (higher than 0.5). Moreover, the derivative of this distance with respect to $P_{+/-}$ (i.e., the proportion of exploitative interactions) is

$$\begin{aligned}\frac{\mathrm{dND}}{\mathrm{d}P_{+/-}} &= \frac{E^2}{V} + \frac{C\mathbb{E}^2(|Z|)}{V} - \frac{C\mathbb{E}(|Z|)E}{2V}\frac{\rho - E^2}{V} \\ &= \frac{E^2}{V} + \frac{C\mathbb{E}^2(|Z|)}{V} - \frac{C\mathbb{E}(|Z|)E}{2V} r\left(M_{ij}, M_{ji}\right)_{i\neq j} \\ &= \frac{E^2}{V} + \frac{C\mathbb{E}(|Z|)}{V}\left(\mathbb{E}(|Z|) - \frac{1}{2}Er\left(M_{ij}, M_{ji}\right)_{i\neq j}\right) \\ &> 0,\end{aligned} \tag{72}$$

meaning that increase of exploitative interaction proportion leads to a higher normalised distance between reactivity and instability.

4 Supplementary Note 4: Empirical microbial communities analysed in our work

4.1 Mouse microbial communities

Mouse microbial communities studied in our work are constructed by Stein *et al.* based on the gLV model [7]. They provided data on intrinsic growth rates of 11 microbial species and pairwise interactions among these species. These 11 microbial species are *Barnesiella*, *und. Lachnospiraceae*, *uncl. Lachnospiraceae*, other microbial species, *Blautia*, *und. uncl. Mollicutes*, *Akkermansia*, *Coprobacillus*, *Clostridium difficile*, *Enterococcus*, and *und. Enterobacteriaceae*. We focus on two feasible and stable steady states (i.e., empirical communities 1-2 in Fig. 3d). Empirical community 1 contains *Blautia*, *und. uncl. Mollicutes*, *Coprobacillus*, and *und. Enterobacteriaceae*. Empirical community 2 contains other microbial species, *Blautia*, *und. uncl. Mollicutes*, *Clostridium difficile*, and *und. Enterobacteriaceae*. For detailed information about these two communities, please refer to Ref [7].

4.2 Soil microbial communities

Soil microbial communities studied in our work are constructed by Friedman *et al.* based on the gLV model [8]. They provided data on intrinsic growth rates of 8 microbial species and pairwise interactions among these species. These 8 microbial species are *Enterbacter aerogenes* (Ea), *Serratia marcescens* (Sm), *Pseudomonas citronellolis* (Pci), *Pseudomonas putida* (Pp), *Pseudomonas aurantiaca* (Pa), *Pseudomonas chlororaphis* (Pch), *Pseudomonas fluorescens* (Pf), and *Pseudomonas veronii* (Pv). We focus on four feasible and stable steady states (i.e., empirical communities 3-6 in Fig. 3d). Empirical community 3 contains Pa, Pci, Pf, and Pv. Empirical community 4 contains Pci, Pf, Pp, and Pv. Empirical community 5 contains Ea, Pci, Pv, and Sm. Empirical community 6 contains Pci, Pp, Pv, and Sm. For detailed information about these communities, please refer to Ref [8].

4.3 Randomly constructed microbial community

Randomly constructed microbial community studied in our work is constructed by Hu *et al.* based on the gLV model [9]. They provided data on intrinsic growth rates of 6 microbial genera and pairwise interactions among these species. These 6 microbial genera are *Leuconostoc* (Leu), *Pseudomonas* (Pse), *Yersinia* (Yer), *Pantoea* (Pan), *Klebsiella* (Kle), and *Acinetobacter* (Aci). We focus on one feasible and stable steady state (empirical community 7 in Fig. 3d), which contains Leu, Yer, Pan, Kle, and Aci. For detailed information about this community, please refer to Ref [9].

5 Supplementary Note 5: Influences of empirical food web structures

Previous theoretical analyses deal with unstructured ecological networks. However, natural communities can hardly be unstructured, and various studies show that empirical ecological network structures have important influences on ecological stability and many other properties [1,3,10–16]. Here we want to assess how these structural features affect reactivity by studying two widely-used food web models and their variants.

5.1 Cascade food web

Cascade model, a widely-used food web model [1,3,10,15–17], introduces trophic levels into exploitative communities compared with unstructured case. In the cascade model, species form a hierarchy and species with higher rank predate species with lower rank with a fixed probability C. The construction method of the community matrix for a cascade food web is presented in the Methods section.

From the construction method, it is clear that the only difference (which is introduced by trophic levels) between the community matrices of unstructured food web and cascade food web is the arrangement of pairwise positive and negative elements. For the unstructured case, positive and negative elements are mixed well in the whole off-diagonal part of the community matrix. For the cascade case, all positive elements are in the upper-triangular part and all negative elements are in the lower-triangular part. This difference, however, is broken in matrix $\mathbf{H}$, since $H_{ij} = H_{ji} = (M_{ij} + M_{ji})/2$. When all other parameters are the same, unstructured case and cascade case yield the same $\mathbf{H}$. Thus, cascade model has no influence on reactivity, as is presented in the main text.

5.2 Niche food web

Niche model is another widely-used food web model [1,10,14,15]. The species in a niche model are ordered by its 'niche value', η_i. A niche 'radius' r_i, which is proportional to η_i, is drawn for each species along with a 'niche center' c_i. Species i will prey all species j whose niche value η_j is in the range of $\left[c_i - \frac{1}{2}r_i, c_i + \frac{1}{2}r_i\right]$. Therefore, compared with unstructured case, niche model introduces not only trophic levels, but also intervality (i.e., each predator only preys upon species with consecutive niche values) and broad degree distribution [10] (Fig. 4c). Here degree of a species is the number of its interacting species. The construction method of the community matrix for a niche food web is presented in the Methods section. Please note that in cases where two species predate each other (i.e.,

species i preys on species j and simultaneously, species j preys on species i), we randomly designate one species as the predator and the other as the prey.

Compared with unstructured case and cascade case, niche model affects not only the arrangement of pairwise positive and negative elements in the community matrix, but also the number of interacting species of each species. In former two cases, each species has almost the same number of interacting species when community size is sufficiently large, i.e., unstructured case and cascade case yield almost the same degree distribution, which is relatively narrow (Fig. 4c). Niche model, however, brings broad degree distribution: the number of interacting species of some species in niche model is higher than average while that of other species is lower than average (Fig. 4c). Moreover, niche model also introduces intervality. These two properties are not mediated in matrix $\mathbf{H}$. Thus, $\mathbf{H}_{\text{niche}}$ is not equivalent to $\mathbf{H}_{\text{unstructured}}$, and niche model makes the system more reactive.

5.3 Interval cascade food web

Previous sections show that cascade model has no influence on reactivity while niche model makes the system more reactive. The main differences between cascade model and niche model are as follows: niche model introduces intervality and broader degree distribution. We want to specify which feature is the main driver to the distinct effects on reactivity. Thus, in this section and following sections, we construct three variants of cascade model (interval cascade food web, cascade food web with broad degree distribution, and interval cascade food web with broad degree distribution) to answer this question.

The community matrix of an interval cascade model can be constructed as follows [10]:

Step 1. For each species i, we first determine the number of its preys L_i as $L_i = C(i-1)$.

Step 2. Then, we sample a random starting point s_i from the uniform discrete distribution $U\{1, \cdots, i - L_i\}$ for each species i.

Step 3. For each $j \in [s_i, s_i + L_i - 1]$, set $K_{ij} = 1$ (this means species i predates all species from s_i to $s_i + L_i - 1$).

Step 4. For each $K_{ij} = 1$, sample 2 variables in pair from $|Z|$ and $-|Z|$ respectively. Then, assign the negative one to $M_{ij,i<j}$ and the positive one to $M_{ij,i>j}$. Z is a random variable with mean 0 and variance σ^2.

Step 5. Set the diagonal elements of $\mathbf{M}$ to $-d$.

On average, we can see that compared with unstructured case (and equivalently, cascade case), the reactivity of an interval cascade model increases slightly (Supplemental Figs. S5, S6). And the

introduction of intervality has little influence on degree distribution: the degree distribution is slightly changed with the presence of several nodes with relatively low degree. We can thus regard intervality and degree distribution as irrelevant variables.

5.4 Cascade food web with broad degree distribution

In this section we consider the construction of a cascade model with broad degree distribution by borrowing the idea from the construction methods of niche model. The corresponding community matrix can be constructed as follows [10]:

Step 1. For each species, we set a niche value η by sampling from a uniform distribution $U[0,1]$ and sort these species in increasing order (i.e., $\eta_1 < \eta_2 < \cdots < \eta_S$).

Step 2. For each species i, we set a niche radius r_i. $r_i = \eta_i \mathcal{B}$ and $\mathcal{B}$ is sampled from the Beta distribution $\text{Be}(1, 1/C - 1)$ (this ensures the connectance C can be maintained).

Step 3. For each species i, we set the number of resources (i.e., consumer-degree) L_i by sampling from a Binomial distribution $\text{B}(i-1, r_i)$.

Step 4. For each species i, we then generate a effective connectance $C_{\text{eff},i} = L_i/(i-1)$ ($C_{\text{eff},1} = 0$).

Step 5. For each element in the upper-triangular part of $\mathbf{K}$ (i.e., $K_{ij,i<j}$), draw a random variable p_1.

Step 6. If $p_1 < C_{\text{eff},j}$, we set $K_{ij} = 1$ and $K_{ji} = 0$. If $p_1 > C_{\text{eff},j}$, we set $K_{ij} = K_{ji} = 0$.

Step 7. For each $K_{ij} = 1$, sample 2 variables in pair from $|Z|$ and $-|Z|$ respectively. Then, assign the negative one to $M_{ij,i<j}$ and the positive one to $M_{ij,i>j}$. Z is a random variable with mean 0 and variance σ^2.

Step 8. Set the diagonal elements of $\mathbf{M}$ to $-d$.

Through the construction method stated above, the degree distribution widens (Supplemental Fig. S7). Compared with interval cascade model, the reactivity increase of cascade model with broad degree distribution is more notable (Supplemental Figs. S5, S6) on average, leading to the conjecture that change in degree distribution is the main driver to the effect that niche model has on reactivity.

5.5 Interval cascade food web with broad degree distribution

In this section, we consider interval cascade model and broad degree distribution. The corresponding community matrix is constructed as follows [10]:

Step 1. For each species, we set a niche value η by sampling from a uniform distribution $U[0,1]$ and sort these species in increasing order (i.e., $\eta_1 < \eta_2 < \cdots < \eta_S$).

Step 2. For each species i, we set a niche radius r_i. $r_i = \eta_i \mathcal{B}$ and $\mathcal{B}$ is sampled from a Beta distribution $\mathrm{Be}(1, 1/C - 1)$.

Step 3. For each species i, we set the number of resources (i.e., consumer-degree) L_i by sampling from a Binomial distribution $\mathrm{B}(i-1, r_i)$.

Step 4. Then we sample a random starting point s_i from the uniform discrete distribution $U\{1, \cdots, i - L_i\}$ for each species i.

Step 5. For each $j \in [s_i, s_i + L_i - 1]$, set $K_{ij} = 1$.

Step 6. For each $K_{ij} = 1$, sample 2 variables in pair from $|Z|$ and $-|Z|$ respectively. Then, assign the negative one to $M_{ij,i<j}$ and the positive one to $M_{ij,i>j}$. Z is a random variable with mean 0 and variance σ^2.

Step 7. Set the diagonal elements of $\mathbf{M}$ to $-d$.

The comparison among cascade model and its three variants (Supplemental Figs. S5, S6) proves our conjecture above: on average, change in degree distribution is the main driver to the effect that niche model has on the reactivity of exploitative communities.

6 Supplementary Note 6: Self-regulation and reactivity of complex ecosystems

Self-regulation is believed to play an important role in shaping ecosystem dynamics [1, 3, 18], and it is reasonable to deduce that self-regulation can significantly shape system reactivity. In the so-far constructed theoretical framework for reactivity analysis, which assumes that all species have homogeneous self-regulation strengths (i.e., $M_{ii} = -d$), we can quickly draw the conclusion that strong self-regulation leads to a less reactive community. However, species in natural communities tend to have heterogeneous self-regulation strengths, and there are studies suggesting that some species do not need to self-regulate [3, 19, 20]. We are then wondering how such heterogeneity of self-regulation strengths and non-self-regulating species influence system reactivity. Luckily, recent development [3–5, 21] of random matrix theory enables us to incorporate heterogeneous self-regulation strengths and non-self-regulating species into our analysis framework.

In this section, we will first introduce the concept of quaternions and the method of quaternionic resolvents, which are the essential mathematical tools to conduct theoretical analysis. Then, we will incorporate heterogeneity of self-regulation strengths and non-self-regulating species into reactivity analysis respectively.

6.1 Quaternions and the method of quaternionic resolvents

6.1.1 Quaternions

By defining the imaginary unit i with the property $\mathrm{i}^2 = -1$, mathematicians introduce the concept of complex number. A complex number z can be written as $z = a + b \cdot \mathrm{i}$, where $a,\ b \in \mathbb{R}$. Similar to this construction method, two symbols j and k with following properties

$$\mathrm{i}^2 = \mathrm{j}^2 = \mathrm{k}^2 = \mathrm{ijk} = -1,\ \mathrm{ij} = \mathrm{k},\ \mathrm{ji} = -\mathrm{k},\ \mathrm{jk} = \mathrm{i},\ \mathrm{kj} = -\mathrm{i},\ \mathrm{ki} = \mathrm{j},\ \mathrm{ik} = -\mathrm{j}, \tag{73}$$

and the concept of **quaternion** are introduced [22]. A quaternion can be expressed as

$$q = a + b \cdot \mathrm{i} + c \cdot \mathrm{j} + d \cdot \mathrm{k}, \tag{74}$$

where $a,\ b,\ c,\ d \in \mathbb{R}$. The set of quaternions is denoted as $\mathbb{H}$. By using the property $\mathrm{ij} = \mathrm{k}$, any quaternion $q \in \mathbb{H}$ can be equivalently written as

$$q = a + b \cdot \mathrm{i} + c \cdot \mathrm{j} + d \cdot \mathrm{k} = z + u \cdot \mathrm{j}, \tag{75}$$

where z and u are two complex numbers and $z = a + b \cdot \mathrm{i}$ and $u = c + d \cdot \mathrm{i}$.

6.1.2 The method of quaternionic resolvents

Suppose $\mathbf{N}$ is a $S \times S$ matrix with spectral density function $w(x),\ x \in \mathbb{C}$. Here spectral density function of $\mathbf{N}$ is defined as

$$w\left(x\right) = \frac{1}{S} \sum_{i=1}^{S} \delta\left(\lambda_i - x\right), \tag{76}$$

where λ_i is the eigenvalue of matrix $\mathbf{N}$, $x \in \mathbb{C}$ and $\delta(\cdot)$ is the Dirac delta function. The resolvent of a general matrix $\mathbf{N}$ is

$$G(z) = \int_{\mathbb{C}} \frac{w(s)}{s - z} \mathrm{d}s, \tag{77}$$

and the integration goes across the whole complex plane since the eigenvalues are in general complex. The resolvent has pole singularities (i.e., the denominator equals zero) when z is equal to one of the eigenvalues. Thus, researchers introduce quaternionic resolvent $G(q) \in \mathbb{H}$ [21], which is a function of $q \in \mathbb{H}$. Then, the resolvent of $\mathbf{N}$ becomes

$$G(q) = \int_{\mathbb{C}} w(s)(s - q)^{-1} \mathrm{d}s. \tag{78}$$

Since quaternionic multiplication is not commutative, here we use the inverse notation for quaternions. As long as $q = m + n \cdot \mathrm{j}$ satisfies $n \neq 0$, resolvent above is well-defined.

The spectral density function can be recovered from the resolvent as

$$w(x) = -\frac{1}{\pi} \lim_{\varepsilon \to 0^+} \mathrm{Re}\left(\frac{\partial}{\partial \bar{x}} G(x + \varepsilon \cdot \mathrm{j})\right), \tag{79}$$

where $\mathrm{Re}(\cdot)$ represents the real part, ε approaches zero from positive values, and $\partial / \partial \bar{x}$ is the anti-holomorphic derivative, which can be written as

$$\frac{\partial}{\partial \bar{x}} = \frac{1}{2}\left(\frac{\partial}{\partial a} + \mathrm{i} \cdot \frac{\partial}{\partial b}\right), \tag{80}$$

if $x = a + b \cdot \mathrm{i}$ with $a,\ b \in \mathbb{R}$. The spectral density function can be rewritten as

$$w\left(a+b\cdot\mathrm{i}\right) = -\frac{1}{2\pi}\lim_{\varepsilon\to 0^+}\mathrm{Re}\left(\left(\frac{\partial}{\partial a}+\mathrm{i}\cdot\frac{\partial}{\partial b}\right)G(a+b\cdot\mathrm{i}+\varepsilon\cdot\mathrm{j})\right). \tag{81}$$

This limit must be taken after the real part of the derivative is evaluated in principle. However, this only makes a difference if the derivative behaves singularly at $\varepsilon = 0$. Since in our cases, the limit never shows singularity at $\varepsilon = 0$, it thus can be taken as

$$w(a+b\cdot i) = -\frac{1}{2\pi}\mathrm{Re}\left(\left(\frac{\partial}{\partial a}+\mathrm{i}\cdot\frac{\partial}{\partial b}\right)G(a+b\cdot\mathrm{i})\right). \tag{82}$$

The spectral density function of the sum of matrix $\mathbf{N}$ and another 'deterministic' matrix $\mathbf{D}$ with known spectral distribution $p(h)$ can be calculated by the resolvent. Let $\mathbf{N}$ be a standard elliptic matrix with correlation $\mathbb{E}(N_{ij}N_{ji})_{i\neq j} = \tau/S$, Rogers [21] suggested that the resolvent of $\mathbf{N}+\mathbf{D}$ can be given by

$$G(q) = G_{\mathbf{D}}\left(q + (\tau+\mathrm{j})\circ G(q)\right). \tag{83}$$

Here $\circ$ represents entrywise product. The entrywise product of two quaternions q_1 and q_2 is defined as

$$q_1 \circ q_2 = (z_1 + u_1\cdot\mathrm{j})\circ(z_2+u_2\cdot\mathrm{j}) = z_1z_2 + u_1u_2\cdot\mathrm{j}. \tag{84}$$

Since the spectral density function of matrix $\mathbf{D}$ is $p\left(h\right)$, the resolvent of $\mathbf{D}$ can be written as

$$G_{\mathbf{D}}\left(q\right) = \int_{\mathbb{C}} p\left(s\right)\left(s-q\right)^{-1}\mathrm{d}s. \tag{85}$$

Then, we can obtain following equation

$$G(q) = \int_{\mathbb{C}} p\left(s\right)\left(s-q-(\tau+\mathrm{j})\circ G\left(q\right)\right)^{-1}\mathrm{d}s. \tag{86}$$

Then, we show how to solve this resolvent when $\mathbf{N}$ is a standard elliptic matrix [3]. Substitute q with $x+\varepsilon\cdot j$ and taking the $\varepsilon\to 0^+$ limit, this simplifies equation above as

$$G(x) = \int_{\mathbb{C}} p\left(s\right)\left(s-x-(\tau+\mathrm{j})\circ G\left(x\right)\right)^{-1}\mathrm{d}s. \tag{87}$$

Since G is a quaternion, we can let $G = \alpha + \beta \cdot j$ and obtain

$$\begin{aligned} \alpha + \beta \cdot \mathrm{j} &= \int_{\mathbb{C}} p(s)(s - x - \tau\alpha - \beta \cdot \mathrm{j})^{-1} \mathrm{d}s \\ &= \int_{\mathbb{C}} p(s) \frac{\bar{s} - \bar{x} - \tau\bar{\alpha} + \beta \cdot \mathrm{j}}{|s - x - \tau\alpha|^2 + |\beta|^2} \mathrm{d}s. \end{aligned} \tag{88}$$

Two quaternions $q_1 = m_1 + n_1 \cdot \mathrm{j}$ and $q_2 = m_2 + n_2 \cdot \mathrm{j}$ are equal if and only if $m_1 = m_2$ and $n_1 = n_2$. Thus, we have

$$\begin{cases} \alpha = \int_{\mathbb{C}} p(s) \frac{\bar{s} - \bar{x} - \tau\bar{\alpha}}{|s - x - \tau\alpha|^2 + |\beta|^2} \mathrm{d}s, \\ \beta = \int_{\mathbb{C}} p(s) \frac{\beta}{|s - x - \tau\alpha|^2 + |\beta|^2} \mathrm{d}s. \end{cases} \tag{89}$$

It is clear that $\beta = 0$ is always a solution. Whether this solution leads to a meaningful spectral density function should be checked by solving for the corresponding α and examining the $w(x)$ obtained. For cases when $\beta \neq 0$, equations above become

$$\begin{cases} \alpha = \int_{\mathbb{C}} p(s) \frac{\bar{s} - \bar{x} - \tau\bar{\alpha}}{|s - x - \tau\alpha|^2 + |\beta|^2} \mathrm{d}s, \\ 1 = \int_{\mathbb{C}} p(s) \frac{1}{|s - x - \tau\alpha|^2 + |\beta|^2} \mathrm{d}s. \end{cases} \tag{90}$$

Rewrite α ($\alpha \in \mathbb{C}$) as $\alpha = \alpha_{\mathrm{re}} + \alpha_{\mathrm{im}} \cdot \mathrm{i}$ ($\alpha_{\mathrm{re}},\ \alpha_{\mathrm{im}} \in \mathbb{R}$), we obtain

$$\begin{cases} \alpha_{\mathrm{re}} + \alpha_{\mathrm{im}} \cdot \mathrm{i} = \int_{\mathbb{C}} p(s) \frac{\bar{s} - \bar{x} - \tau(\alpha_{\mathrm{re}} - \alpha_{\mathrm{im}} \cdot \mathrm{i})}{|s - x - \tau(\alpha_{\mathrm{re}} + \alpha_{\mathrm{im}} \cdot \mathrm{i})|^2 + |\beta|^2} \mathrm{d}s, \\ 1 = \int_{\mathbb{C}} p(s) \frac{1}{|s - x - \tau(\alpha_{\mathrm{re}} + \alpha_{\mathrm{im}} \cdot \mathrm{i})|^2 + |\beta|^2} \mathrm{d}s. \end{cases} \tag{91}$$

Variable x ($x \in \mathbb{C}$) can also be written as $x = x_{\mathrm{re}} + x_{\mathrm{im}} \cdot \mathrm{i}$, we can then have

$$\begin{cases} \alpha_{\mathrm{re}} + \alpha_{\mathrm{im}} \cdot \mathrm{i} = \int_{\mathbb{C}} p(s) \frac{\bar{s} - (x_{\mathrm{re}} - x_{\mathrm{im}} \cdot \mathrm{i}) - \tau(\alpha_{\mathrm{re}} - \alpha_{\mathrm{im}} \cdot \mathrm{i})}{|s - x_{\mathrm{re}} - x_{\mathrm{im}} \cdot \mathrm{i} - \tau(\alpha_{\mathrm{re}} + \alpha_{\mathrm{im}} \cdot \mathrm{i})|^2 + |\beta|^2} \mathrm{d}s, \\ 1 = \int_{\mathbb{C}} p(s) \frac{1}{|s - x_{\mathrm{re}} - x_{\mathrm{im}} \cdot \mathrm{i} - \tau(\alpha_{\mathrm{re}} + \alpha_{\mathrm{im}} \cdot \mathrm{i})|^2 + |\beta|^2} \mathrm{d}s, \end{cases} \tag{92}$$

which further leads to

$$\begin{cases} \alpha_{\mathrm{re}} + \alpha_{\mathrm{im}} \cdot \mathrm{i} = \int_{\mathbb{C}} p(s) \frac{\bar{s} - (x_{\mathrm{re}} - x_{\mathrm{im}} \cdot \mathrm{i}) - \tau(\alpha_{\mathrm{re}} - \alpha_{\mathrm{im}} \cdot \mathrm{i})}{(s - x_{\mathrm{re}} - \tau\alpha_{\mathrm{re}})^2 + (x_{\mathrm{im}} + \tau\alpha_{\mathrm{im}})^2 + |\beta|^2} \mathrm{d}s, \\ 1 = \int_{\mathbb{C}} p(s) \frac{1}{(s - x_{\mathrm{re}} - \tau\alpha_{\mathrm{re}})^2 + (x_{\mathrm{im}} + \tau\alpha_{\mathrm{im}})^2 + |\beta|^2} \mathrm{d}s. \end{cases} \tag{93}$$

These equations are equivalent to following equations

$$\begin{cases} \alpha_{\mathrm{re}} = \int_{\mathbb{C}} p(s) \frac{\mathrm{Re}(s) - x_{\mathrm{re}} - \tau\alpha_{\mathrm{re}}}{(s - x_{\mathrm{re}} - \tau\alpha_{\mathrm{re}})^2 + (x_{\mathrm{im}} + \tau\alpha_{\mathrm{im}})^2 + |\beta|^2} \mathrm{d}s, \\ \alpha_{\mathrm{im}} = \int_{\mathbb{C}} p(s) \frac{-\mathrm{Im}(s) + x_{\mathrm{im}} + \tau\alpha_{\mathrm{im}}}{(s - x_{\mathrm{re}} - \tau\alpha_{\mathrm{re}})^2 + (x_{\mathrm{im}} + \tau\alpha_{\mathrm{im}})^2 + |\beta|^2} \mathrm{d}s, \\ 1 = \int_{\mathbb{C}} p(s) \frac{1}{(s - x_{\mathrm{re}} - \tau\alpha_{\mathrm{re}})^2 + (x_{\mathrm{im}} + \tau\alpha_{\mathrm{im}})^2 + |\beta|^2} \mathrm{d}s. \end{cases} \tag{94}$$

When the spectral density function $p(x)$ of matrix $\mathbf{D}$ is specified, above three equations can be solved and the spectral density function of matrix $\mathbf{N}+\mathbf{D}$ can be obtained from the solutions.

When matrix $\mathbf{D}$ has purely real eigenvalues, $\mathrm{Re}(s)=s,\ \mathrm{Im}(s)=0$, and equations above become [3]

$$\begin{cases} \alpha_{\mathrm{re}} = \int_{\mathbb{R}} p(s) \frac{s-x_{\mathrm{re}}-\tau\alpha_{\mathrm{re}}}{(s-x_{\mathrm{re}}-\tau\alpha_{\mathrm{re}})^2+(x_{\mathrm{im}}+\tau\alpha_{\mathrm{im}})^2+|\beta|^2} \mathrm{d}s, \\ \alpha_{\mathrm{im}} = \int_{\mathbb{R}} p(s) \frac{x_{\mathrm{im}}+\tau\alpha_{\mathrm{im}}}{(s-x_{\mathrm{re}}-\tau\alpha_{\mathrm{re}})^2+(x_{\mathrm{im}}+\tau\alpha_{\mathrm{im}})^2+|\beta|^2} \mathrm{d}s, \\ 1 = \int_{\mathbb{R}} p(s) \frac{1}{(s-x_{\mathrm{re}}-\tau\alpha_{\mathrm{re}})^2+(x_{\mathrm{im}}+\tau\alpha_{\mathrm{im}})^2+|\beta|^2} \mathrm{d}s. \end{cases} \tag{95}$$

Note that the integrals now only sweep through the real axis. In this case when $\mathbf{D}$ has purely real eigenvalues, the equation that determines α_{im} can be further written as

$$\begin{aligned} \alpha_{\mathrm{im}} &= (x_{\mathrm{im}}+\tau\alpha_{\mathrm{im}}) \int_{\mathbb{R}} p(s) \frac{1}{(s-x_{\mathrm{re}}-\tau\alpha_{\mathrm{re}})^2+(x_{\mathrm{im}}+\tau\alpha_{\mathrm{im}})^2+|\beta|^2} \mathrm{d}s \\ &= x_{\mathrm{im}}+\tau\alpha_{\mathrm{im}}. \end{aligned} \tag{96}$$

Thus,

$$\alpha_{\mathrm{im}} = \frac{x_{\mathrm{im}}}{1-\tau}. \tag{97}$$

Substitute this solution of α_{im} back to Eq. (95)

$$\begin{cases} \alpha_{\mathrm{re}} = \int_{\mathbb{R}} p(s) \frac{s-x_{\mathrm{re}}-\tau\alpha_{\mathrm{re}}}{(s-x_{\mathrm{re}}-\tau\alpha_{\mathrm{re}})^2+x_{\mathrm{im}}^2/(1-\tau)^2+|\beta|^2} \mathrm{d}s, \\ 1 = \int_{\mathbb{R}} p(s) \frac{1}{(s-x_{\mathrm{re}}-\tau\alpha_{\mathrm{re}})^2+x_{\mathrm{im}}^2/(1-\tau)^2+|\beta|^2} \mathrm{d}s. \end{cases} \tag{98}$$

Note that derivations in this section (i.e., 6.1.2) can also be found from the work by Barab*á*s *et al.* [3], here we still provide these derivations for clarity.

6.2 Incorporating heterogeneous self-regulation strengths into reactivity analysis

6.2.1 Random community

For simplicity, here we decompose $\mathbf{M}$ as $\mathbf{M}=\mathbf{M}_{\mathrm{intra}}+\mathbf{M}_{\mathrm{inter}}$, where $\mathbf{M}_{\mathrm{intra}}$ is a diagonal matrix which extracts the terms of self-regulations, and $\mathbf{M}_{\mathrm{inter}}$ extracts the terms of interspecies interactions. Note that here we focus on cases where self-regulation strengths are sampled from the uniform distribution $U\left[-d_{\mathrm{mean}}-\sqrt{3}\sigma_d, -d_{\mathrm{mean}}+\sqrt{3}\sigma_d\right]$.

From the construction process of $\mathbf{M}$, we can obtain the statistical features of $\mathbf{M}_{\mathrm{inter}}$: $\mathbb{E}\left(M_{\mathrm{inter},ij}\right) =$

0, $\mathrm{Var}\left(M_{\mathrm{inter},ij}\right) = C\sigma^2$ and $\mathbb{E}\left(M_{\mathrm{inter},ij}M_{\mathrm{inter},ji}\right)_{i\neq j} = 0$. Since $\mathbf{H}_{\mathrm{inter}} = \left(\mathbf{M}_{\mathrm{inter}} + \mathbf{M}_{\mathrm{inter}}^{\mathrm{T}}\right)/2$, we have

$$\begin{cases} \mathbb{E}\left(H_{\mathrm{inter},ij}\right) = 0, \\ \mathrm{Var}\left(H_{\mathrm{inter},ij}\right) = \frac{1}{2}C\sigma^2, \\ \mathbb{E}\left(H_{\mathrm{inter},ij}H_{\mathrm{inter},ji}\right)_{i\neq j} = \frac{1}{2}C\sigma^2. \end{cases} \tag{99}$$

To locate the rightmost eigenvalue of $\mathbf{H} = \mathbf{H}_{\mathrm{inter}} + \mathbf{H}_{\mathrm{intra}}$, we construct a matrix $\mathbf{N} = \mathbf{H}/\sqrt{S \cdot \mathrm{Var}\left(H_{\mathrm{inter},ij}\right)}$. Matrix $\mathbf{N}$ can also be decomposed as

$$\mathbf{N} = \mathbf{N}_{\mathrm{inter}} + \mathbf{N}_{\mathrm{intra}}, \tag{100}$$

where $\mathbf{N}_{\mathrm{inter}} = \mathbf{H}_{\mathrm{inter}}/\sqrt{S \cdot \mathrm{Var}\left(H_{\mathrm{inter},ij}\right)}$, $\mathbf{N}_{\mathrm{intra}} = \mathbf{H}_{\mathrm{intra}}/\sqrt{S \cdot \mathrm{Var}\left(H_{\mathrm{inter},ij}\right)}$. Therefore, we have

$$\begin{cases} \mathbb{E}\left(N_{\mathrm{inter},ij}\right) = 0, \\ \mathrm{Var}\left(N_{\mathrm{inter},ij}\right) = \frac{1}{S}, \\ \mathbb{E}\left(N_{\mathrm{inter},ij}N_{\mathrm{inter},ji}\right)_{i\neq j} = \frac{1}{S}, \end{cases} \tag{101}$$

and the diagonal entries of $\mathbf{N}_{\mathrm{intra}}$ are now sampled from a uniform distribution

$$U\left[\left(-d_{\mathrm{mean}} - \sqrt{3}\sigma_d\right)/\sqrt{S \cdot \mathrm{Var}\left(H_{\mathrm{inter},ij}\right)}, \left(-d_{\mathrm{mean}} + \sqrt{3}\sigma_d\right)/\sqrt{S \cdot \mathrm{Var}\left(H_{\mathrm{inter},ij}\right)}\right]. \tag{102}$$

For simplicity, we denote

$$\begin{cases} d_1 = \left(-d_{\mathrm{mean}} - \sqrt{3}\sigma_d\right)/\sqrt{S \cdot \mathrm{Var}\left(H_{\mathrm{inter},ij}\right)}, \\ d_2 = \left(-d_{\mathrm{mean}} + \sqrt{3}\sigma_d\right)/\sqrt{S \cdot \mathrm{Var}\left(H_{\mathrm{inter},ij}\right)}. \end{cases} \tag{103}$$

We can obtain the spectral density of $\mathbf{N}_{\mathrm{intra}}$ as

$$p\left(h\right) = \frac{H(h-d_1) - H(h-d_2)}{d_2 - d_1}, \tag{104}$$

where $H\left(\cdot\right)$ is the Heaviside step function.

According to previous discussions, the equations for the resolvent of matrix $\mathbf{N} = \mathbf{N}_{\mathrm{inter}} + \mathbf{N}_{\mathrm{intra}}$

can be obtained as

$$\begin{cases} 1 = \frac{\tan^{-1}\left(\frac{x+\tau\alpha_{\rm re}-d_2}{\sqrt{y^2/(1-\tau^2)+|\beta|^2}}\right)-\tan^{-1}\left(\frac{x+\tau\alpha_{\rm re}-d_1}{\sqrt{y^2/(1-\tau^2)+|\beta|^2}}\right)}{(d_1-d_2)\sqrt{y^2/(1-\tau^2)+|\beta|^2}}, \\ \alpha_{\rm re} = \frac{1}{2(d_2-d_1)}\log\left(\frac{(x+\tau\alpha_{\rm re}-d_2)^2+y^2/\left(1-\tau^2\right)+|\beta|^2}{(x+\tau\alpha_{\rm re}-d_1)^2+y^2/(1-\tau^2)+|\beta|^2}\right), \end{cases} \tag{105}$$

where $x = \mathrm{Re}\,(\lambda_{\mathbf{N}})$, $y = \mathrm{Im}\,(\lambda_{\mathbf{N}})$. Since $\mathbf{N}$ is a symmetric matrix, all eigenvalues of $\mathbf{N}$ are real numbers, bringing $y = 0$. Moreover, the symmetric nature also leads to $\tau = 1$. Thus, equations above can be written as

$$\begin{cases} 1 = \frac{\tan^{-1}\left(\frac{x+\alpha_{\rm re}-d_2}{|\beta|}\right)-\tan^{-1}\left(\frac{x+\alpha_{\rm re}-d_1}{|\beta|}\right)}{(d_1-d_2)|\beta|}, \\ \alpha_{\rm re} = \frac{1}{2(d_2-d_1)}\log\left(\frac{(x+\alpha_{\rm re}-d_2)^2+|\beta|^2}{(x+\alpha_{\rm re}-d_1)^2+|\beta|^2}\right), \end{cases} \tag{106}$$

Since when $|\beta|$ equals exactly zero, the corresponding complex numbers of $x + y \cdot \mathrm{i}$ locate on the outer edge of the spectral distribution. Therefore, in our case, taking the $|\beta| \to 0$ limit can lead to the rightmost eigenvalue and the leftmost eigenvalue of the eigenvalue distribution. We can now obtain following equations

$$\begin{cases} 1 = \frac{1}{(x+\alpha_{\rm re}-d_2)(x+\alpha_{\rm re}-d_1)}, \\ \alpha_{\rm re} = \frac{1}{d_2-d_1}\log\left(\left|\frac{x+\alpha_{\rm re}-d_2}{x+\alpha_{\rm re}-d_1}\right|\right). \end{cases} \tag{107}$$

To decouple the equations above, we now introduce a variable $\tilde{x} = x + \alpha_{\rm re}$ and have

$$\begin{cases} 1 = \frac{1}{(\tilde{x}-d_2)(\tilde{x}-d_1)}, \\ \alpha_{\rm re} = \frac{1}{d_2-d_1}\log\left(\left|\frac{\tilde{x}-d_2}{\tilde{x}-d_1}\right|\right). \end{cases} \tag{108}$$

$\tilde{x}$ can be obtained by solving the first sub-equation of Eq. (108)

$$\begin{cases} \tilde{x}_1 = \frac{d_1+d_2}{2} + \sqrt{\left(\frac{d_2-d_1}{2}\right)^2 + 1}, \\ \tilde{x}_2 = \frac{d_1+d_2}{2} - \sqrt{\left(\frac{d_2-d_1}{2}\right)^2 + 1}. \end{cases} \tag{109}$$

Substituting $\tilde{x}$ back into the second sub-equation of Eq. (108) brings us $\alpha_{\rm re}$

$$\begin{cases} \alpha_{\rm re,1} = -\frac{2}{d_2-d_1}\tanh^{-1}\left(\frac{d_2-d_1}{\sqrt{(d_2-d_1)^2+4}}\right), \\ \alpha_{\rm re,2} = \frac{2}{d_2-d_1}\tanh^{-1}\left(\frac{d_2-d_1}{\sqrt{(d_2-d_1)^2+4}}\right) \end{cases} \tag{110}$$

Therefore, we can get the solutions for x

$$\begin{cases} x_1 = \tilde{x}_1 - \alpha_{\mathrm{re},1} = \frac{d_1+d_2}{2} + \sqrt{\left(\frac{d_2-d_1}{2}\right)^2 + 1} + \frac{2}{d_2-d_1}\tanh^{-1}\left(\frac{d_2-d_1}{\sqrt{(d_2-d_1)^2+4}}\right), \\ x_2 = \tilde{x}_2 - \alpha_{\mathrm{re},2} = \frac{d_1+d_2}{2} - \sqrt{\left(\frac{d_2-d_1}{2}\right)^2 + 1} - \frac{2}{d_2-d_1}\tanh^{-1}\left(\frac{d_2-d_1}{\sqrt{(d_2-d_1)^2+4}}\right). \end{cases} \tag{111}$$

Since x_1 now denotes the rightmost eigenvalue of $\mathbf{N}$, and x_2 denotes the leftmost eigenvalue of $\mathbf{N}$, we have

$$\begin{cases} \lambda_{\mathbf{N},1} = \frac{d_1+d_2}{2} + \sqrt{\left(\frac{d_2-d_1}{2}\right)^2 + 1} + \frac{2}{d_2-d_1}\tanh^{-1}\left(\frac{d_2-d_1}{\sqrt{(d_2-d_1)^2+4}}\right), \\ \lambda_{\mathbf{N},2} = \frac{d_1+d_2}{2} - \sqrt{\left(\frac{d_2-d_1}{2}\right)^2 + 1} - \frac{2}{d_2-d_1}\tanh^{-1}\left(\frac{d_2-d_1}{\sqrt{(d_2-d_1)^2+4}}\right). \end{cases} \tag{112}$$

The eigenvalue distribution of $\mathbf{N}$ can be obtained as: eigenvalues of $\mathbf{N}$ are contained in a line segment with left endpoint $(\lambda_{\mathbf{N},2}, 0)$ and right endpoint $(\lambda_{\mathbf{N},1}, 0)$ on the real axis. And the eigenvalue distribution of $\mathbf{H}$ is thus: eigenvalues of $\mathbf{H}$ are contained in a line segment with left endpoint $\left(\lambda_{\mathbf{N},2}\sqrt{\frac{1}{2}SC\sigma^2}, 0\right)$ and right endpoint $\left(\lambda_{\mathbf{N},1}\sqrt{\frac{1}{2}SC\sigma^2}, 0\right)$ on the real axis (Supplemental Fig. S8).

The reactivity of a random community where all species have heterogeneous self-regulation strengths (self-regulation strengths are sampled from a uniform distribution) can then be obtained

$$\mathcal{R} = \frac{\sqrt{2}}{2}\sigma\sqrt{SC}\left(\frac{d_1+d_2}{2} + \sqrt{\left(\frac{d_2-d_1}{2}\right)^2 + 1} + \frac{2}{d_2-d_1}\tanh^{-1}\left(\frac{d_2-d_1}{\sqrt{(d_2-d_1)^2+4}}\right)\right), \tag{113}$$

where

$$\begin{cases} d_1 = \left(-\sqrt{2}d_{\mathrm{mean}} - \sqrt{6}\sigma_d\right)/\sigma\sqrt{SC}, \\ d_2 = \left(-\sqrt{2}d_{\mathrm{mean}} + \sqrt{6}\sigma_d\right)/\sigma\sqrt{SC}. \end{cases} \tag{114}$$

Clearly, $d_2 - d_1 = 2\sqrt{6}\sigma_d/\sigma\sqrt{SC}$, which is proportional to σ_d. To evaluate the influence of the heterogeneity of self-regulation on the reactivity of random communities, we here introduce two new variables

$$\begin{cases} \tilde{d} = \frac{d_1+d_2}{2}, \\ \tilde{\sigma}_d = d_2 - d_1. \end{cases} \tag{115}$$

It is clear that $\tilde{\sigma}_d > 0$. The expression of reactivity now becomes

$$\mathcal{R} = \frac{\sqrt{2}}{2}\sigma\sqrt{SC}\left(\tilde{d} + \sqrt{\left(\frac{\tilde{\sigma}_d}{2}\right)^2 + 1} + \frac{2}{\tilde{\sigma}_d}\tanh^{-1}\left(\frac{\tilde{\sigma}_d}{\sqrt{\tilde{\sigma}_d^2+4}}\right)\right). \tag{116}$$

Let $f\left(\tilde{\sigma}_d\right)=\tilde{d}+\sqrt{\left(\frac{\tilde{\sigma}_d}{2}\right)^2+1}+\frac{2}{\tilde{\sigma}_d}\tanh^{-1}\left(\frac{\tilde{\sigma}_d}{\sqrt{\tilde{\sigma}_d^2+4}}\right)$, we have

$$\frac{\mathrm{d}f}{\mathrm{d}\tilde{\sigma}_d}=\frac{1}{2\tilde{\sigma}_d}\left(\sqrt{\tilde{\sigma}_d^2+4}-\frac{4}{\tilde{\sigma}_d}\tanh^{-1}\left(\frac{\tilde{\sigma}_d}{\sqrt{\tilde{\sigma}_d^2+4}}\right)\right). \tag{117}$$

Then, let $g\left(\tilde{\sigma}_d\right)=\sqrt{\tilde{\sigma}_d^2+4}-\frac{4}{\tilde{\sigma}_d}\tanh^{-1}\left(\frac{\tilde{\sigma}_d}{\sqrt{\tilde{\sigma}_d^2+4}}\right)$, we can obtain

$$\frac{\mathrm{d}g}{\mathrm{d}\tilde{\sigma}_d}=\frac{1}{\tilde{\sigma}_d^2\sqrt{\tilde{\sigma}_d^2+4}}\left(\tilde{\sigma}_d^3-4\tilde{\sigma}_d+4\tanh^{-1}\left(\frac{\tilde{\sigma}_d}{\sqrt{\tilde{\sigma}_d^2+4}}\right)\right). \tag{118}$$

We then introduce a third function $m\left(\tilde{\sigma}_d\right)=\tilde{\sigma}_d^3-4\tilde{\sigma}_d$. Since $\tilde{\sigma}_d>0$, $m_{\min}\left(\tilde{\sigma}_d\right)=m\left(2\sqrt{3}/3\right)=0$. Thus, $\mathrm{d}g/\mathrm{d}\tilde{\sigma}_d>0$, and $g\left(\tilde{\sigma}_d\right)>\lim_{\tilde{\sigma}_d\to 0}g\left(\tilde{\sigma}_d\right)=0$. This means that $f\left(\tilde{\sigma}_d\right)$ is a monotonically increasing function of $\tilde{\sigma}_d$ for fixed $\tilde{d}$. Therefore, for fixed d_{mean}, $\mathcal{R}$ is also a monotonically increasing function of σ_d, suggesting that the increase of self-regulation heterogeneity makes a random community more reactive.

6.2.2 Community with mixed types of interactions

We still decompose $\mathbf{M}$ as $\mathbf{M}=\mathbf{M}_{\text{inter}}+\mathbf{M}_{\text{intra}}$, and focus on cases where self-regulation strengths are sampled from the uniform distribution $U\left[-d_{\text{mean}}-\sqrt{3}\sigma_d,-d_{\text{mean}}+\sqrt{3}\sigma_d\right]$. The statistics of $\mathbf{M}_{\text{inter}}$ can be obtained as

$$\begin{cases}\mathbb{E}\left(M_{\text{inter},ij}\right)=C\mathbb{E}\left(|Z|\right)\left(P_{+/+}-P_{\text{-/-}}\right)\equiv E,\\ \text{Var}\left(M_{\text{inter},ij}\right)=\mathbb{E}\left(M_{\text{inter},ij}^2\right)-\mathbb{E}^2\left(M_{\text{inter},ij}\right)=C\sigma^2-E^2\equiv V,\\ \mathbb{E}\left(M_{\text{inter},ij}M_{\text{inter},ji}\right)_{i\neq j}=C\mathbb{E}^2\left(|Z|\right)\left(P_{+/+}+P_{\text{-/-}}-P_{+/\text{-}}\right)\equiv\rho.\end{cases} \tag{119}$$

Since $\mathbf{H}_{\text{inter}}=\left(\mathbf{M}_{\text{inter}}+\mathbf{M}_{\text{inter}}^{\mathrm{T}}\right)/2$, we have

$$\begin{cases}\mathbb{E}\left(H_{\text{inter},ij}\right)=E,\\ \text{Var}\left(H_{\text{inter},ij}\right)=\frac{1}{2}\left(V+\rho-E^2\right),\\ \mathbb{E}\left(H_{\text{inter},ij}H_{\text{inter},ji}\right)_{i\neq j}=\frac{1}{2}\left(V+\rho+E^2\right).\end{cases} \tag{120}$$

To locate the rightmost eigenvalue of $\mathbf{H}$, we rewrite $\mathbf{H}$ as

$$\begin{aligned} \mathbf{H} &= \left(\mathbf{H} - E \cdot \mathbf{1} \cdot \mathbf{1}^{\mathrm{T}} + E\mathbf{I}\right) + E \cdot \mathbf{1} \cdot \mathbf{1}^{\mathrm{T}} - E\mathbf{I} \\ &= \mathbf{N} + E \cdot \mathbf{1} \cdot \mathbf{1}^{\mathrm{T}} - E\mathbf{I}, \end{aligned} \tag{121}$$

where $\mathbf{1} \cdot \mathbf{1}^{\mathrm{T}}$ is a $S \times S$ matrix with each entry equaling 1. $\mathbf{N}$ can also be decomposed as

$$\mathbf{N} = \mathbf{N}_{\text{inter}} + \mathbf{N}_{\text{intra}}, \tag{122}$$

where $\mathbf{N}_{\text{inter}} = \mathbf{H}_{\text{inter}} - E \cdot \mathbf{1} \cdot \mathbf{1}^{\mathrm{T}} + E\mathbf{I}$, $\mathbf{N}_{\text{intra}} = \mathbf{H}_{\text{intra}}$. The statistics of $\mathbf{N}_{\text{inter}}$ can then be drawn as

$$\begin{cases} \mathbb{E}\left(N_{\text{inter},ij}\right) = 0, \\ \operatorname{Var}\left(N_{\text{inter},ij}\right) = \frac{1}{2}\left(V + \rho - E^2\right), \\ \mathbb{E}\left(N_{\text{inter},ij} N_{\text{inter},ji}\right)_{i \neq j} = \frac{1}{2}\left(V + \rho - E^2\right). \end{cases} \tag{123}$$

Then, consider matrix

$$\mathbf{N}^* = \frac{\mathbf{N}}{\sqrt{S \cdot \operatorname{Var}\left(N_{\text{inter},ij}\right)}}. \tag{124}$$

$\mathbf{N}^*$ can also be written as

$$\mathbf{N}^* = \mathbf{N}^*_{\text{inter}} + \mathbf{N}^*_{\text{intra}}, \tag{125}$$

where $\mathbf{N}^*_{\text{inter}} = \mathbf{N}^*_{\text{inter}} / \sqrt{S \cdot \operatorname{Var}\left(N_{\text{inter},ij}\right)}$, $\mathbf{N}^*_{\text{intra}} = \mathbf{N}^*_{\text{intra}} / \sqrt{S \cdot \operatorname{Var}\left(N_{\text{inter},ij}\right)}$. We now have

$$\begin{cases} \mathbb{E}\left(N^*_{\text{inter},ij}\right) = 0, \\ \operatorname{Var}\left(N^*_{\text{inter},ij}\right) = \frac{1}{S}, \\ \mathbb{E}\left(N^*_{\text{inter},ij} N_{\text{inter},ji}\right)_{i \neq j} = \frac{1}{S}, \end{cases} \tag{126}$$

and the diagonal entries of $\mathbf{N}^*_{\text{intra}}$ are now sampled from a uniform distribution

$$U\left[\left(-d_{\text{mean}} - \sqrt{3}\sigma_d\right) / \sqrt{S \cdot \operatorname{Var}\left(N_{\text{inter},ij}\right)}, \left(-d_{\text{mean}} + \sqrt{3}\sigma_d\right) / \sqrt{S \cdot \operatorname{Var}\left(N_{\text{inter},ij}\right)}\right]. \tag{127}$$

We still denote

$$\begin{cases} d_1 = \left(-d_{\text{mean}} - \sqrt{3}\sigma_d\right) / \sqrt{S \cdot \operatorname{Var}\left(N_{\text{inter},ij}\right)}, \\ d_2 = \left(-d_{\text{mean}} + \sqrt{3}\sigma_d\right) / \sqrt{S \cdot \operatorname{Var}\left(N_{\text{inter},ij}\right)}, \end{cases} \tag{128}$$

leading to the spectral density of $\mathbf{N}_{\text{intra}}$

$$p(h) = \frac{H(h - d_1) - H(h - d_2)}{d_2 - d_1}, \tag{129}$$

where $H(\cdot)$ is the Heaviside step function.

Similar to the method in random case, we can obtain two endpoints of the eigenvalue distribution of $\mathbf{N}^*$

$$\begin{cases} \lambda_{\mathbf{N}^*,1} = \frac{d_1+d_2}{2} + \sqrt{\left(\frac{d_2-d_1}{2}\right)^2 + 1} + \frac{2}{d_2-d_1} \tanh^{-1}\left(\frac{d_2-d_1}{\sqrt{(d_2-d_1)^2+4}}\right), \\ \lambda_{\mathbf{N}^*,2} = \frac{d_1+d_2}{2} - \sqrt{\left(\frac{d_2-d_1}{2}\right)^2 + 1} - \frac{2}{d_2-d_1} \tanh^{-1}\left(\frac{d_2-d_1}{\sqrt{(d_2-d_1)^2+4}}\right), \end{cases} \tag{130}$$

where $\lambda_{\mathbf{N}^*,1}$ is the rightmost eigenvalue, and $\lambda_{\mathbf{N}^*,2}$ is the leftmost eigenvalue. That is, the eigenvalues of $\mathbf{N}^*$ are distributed in a line segment with left endpoint $(\lambda_{\mathbf{N}^*,2}, 0)$ and right endpoint $(\lambda_{\mathbf{N}^*,1}, 0)$ on the real axis. Therefore, eigenvalues of $\mathbf{N}$ are contained in a line segment with left endpoint $\left(\lambda_{\mathbf{N}^*,2}\sqrt{\frac{1}{2}S(V+\rho-E^2)}, 0\right)$ and right endpoint $\left(\lambda_{\mathbf{N}^*,1}\sqrt{\frac{1}{2}S(V+\rho-E^2)}, 0\right)$ on the real axis.

Now we can turn to matrix $\mathbf{H} = \mathbf{N} + E \cdot \mathbf{1} \cdot \mathbf{1}^{\mathrm{T}} - E \cdot \mathbf{I}$. To derive the eigenvalue distribution of matrix $\mathbf{H}$, we here consider matrix $\mathbf{H}' = \mathbf{H} + d_{\text{mean}}\mathbf{I}$. We also rewrite $\mathbf{H}'$ as

$$\mathbf{H}' = \mathbf{N}' + E \cdot \mathbf{1} \cdot \mathbf{1}^{\mathrm{T}} - E \cdot \mathbf{I}, \tag{131}$$

where $\mathbf{N}' = \mathbf{N} + d_{\text{mean}}\mathbf{I}$. Clearly, the eigenvalues of $\mathbf{N}'$ are distributed in a line segment with left endpoint $\left(d_{\text{mean}} + \lambda_{\mathbf{N}^*,2}\sqrt{\frac{1}{2}S(V+\rho-E^2)}, 0\right)$ and right endpoint $\left(d_{\text{mean}} + \lambda_{\mathbf{N}^*,1}\sqrt{\frac{1}{2}S(V+\rho-E^2)}, 0\right)$, and the diagonal terms of $\mathbf{N}'$ are now sampled from the uniform distribution $\left[-\sqrt{3}\sigma_d, \sqrt{3}\sigma_d\right]$. The statistics of $\mathbf{N}'$ can be derived as follows

$$\begin{cases} \mathbb{E}\left(N'_{ij}\right) = 0, \\ \mathrm{Var}\left(N'_{ij}\right) = \mathbb{E}\left(N'^2_{ij}\right) - \mathbb{E}^2\left(N'_{ij}\right). \end{cases} \tag{132}$$

Since

$$\mathbb{E}\left(N'^2_{ij}\right) = \frac{S(S-1)\,\mathbb{E}\left(N'^2_{ij}\right)_{i\neq j} + S\mathbb{E}\left(N'^2_{ij}\right)_{i=j}}{S^2}, \tag{133}$$

and

$$\begin{cases} \mathbb{E}\left(N'^2_{ij}\right)_{i\neq j} = \mathbb{E}\left(N^2_{\text{inter},ij}\right) = \mathrm{Var}\left(N_{\text{inter},ij}\right) + \mathbb{E}^2\left(N_{\text{inter},ij}\right) = \frac{1}{2}\left(V+\rho-E^2\right), \\ \mathbb{E}\left(N'^2_{ij}\right)_{i=j} = \mathrm{Var}\left(N'_{ij}\right)_{i=j} + \mathbb{E}^2\left(N'_{ij}\right)_{i=j} = \sigma_d^2, \end{cases} \tag{134}$$

for sufficiently large S, we have

$$\mathbb{E}\left(N_{ij}'^2\right) = \frac{1}{2}\left(V + \rho - E^2\right) + \frac{1}{S}\sigma_d^2. \tag{135}$$

Thus,

$$\mathrm{Var}\left(N_{ij}'\right) = \frac{1}{2}\left(V + \rho - E^2\right) + \frac{1}{S}\sigma_d^2. \tag{136}$$

According to low-rank perturbation theorem (note that $\mathbf{H}'$ may not satisfy the requirements of the low-rank perturbation theorem, but we here still assume so, and we find that this assumption is acceptable according to results from numerical simulations), we can obtain the eigenvalue distribution of $\mathbf{H}'$. When $|E| \leq \sqrt{\mathrm{Var}\left(N_{ij}'\right)/S}$, eigenvalues of $\mathbf{H}'$ are contained in a line segment with left endpoint

$$\left(d_{\mathrm{mean}} + \lambda_{\mathbf{N}^*,2}\sqrt{\frac{1}{2}S\left(V + \rho - E^2\right)} - E, 0\right) \tag{137}$$

and right endpoint

$$\left(d_{\mathrm{mean}} + \lambda_{\mathbf{N}^*,1}\sqrt{\frac{1}{2}S\left(V + \rho - E^2\right)} - E, 0\right) \tag{138}$$

on the real axis. When $|E| > \sqrt{\mathrm{Var}\left(N_{ij}'\right)/S}$, a single eigenvalue is equal to $(S-1)\,E + \frac{1}{2E}\left(V + \rho - E^2\right) + \frac{1}{SE}\sigma_d^2$, and $S-1$ eigenvalues are contained in a line segment with left endpoint

$$\left(d_{\mathrm{mean}} + \lambda_{\mathbf{N}^*,2}\sqrt{\frac{1}{2}S\left(V + \rho - E^2\right)} - E, 0\right) \tag{139}$$

and right endpoint

$$\left(d_{\mathrm{mean}} + \lambda_{\mathbf{N}^*,1}\sqrt{\frac{1}{2}S\left(V + \rho - E^2\right)} - E, 0\right) \tag{140}$$

on the real axis.

Thus, we can obtain the eigenvalue distribution of matrix $\mathbf{H}$ (Supplemental Fig. S8). When $|E| \leq \sqrt{\mathrm{Var}\left(N_{ij}'\right)/S}$, eigenvalues of $\mathbf{H}$ are contained in a line segment with left endpoint

$$\left(\lambda_{\mathbf{N}^*,2}\sqrt{\frac{1}{2}S\left(V + \rho - E^2\right)} - E, 0\right) \tag{141}$$

and right endpoint

$$\left(\lambda_{\mathbf{N}^*,1}\sqrt{\frac{1}{2}S\left(V + \rho - E^2\right)} - E, 0\right) \tag{142}$$

on the real axis. When $|E| > \sqrt{\mathrm{Var}\left(N'_{ij}\right)/S}$, a single eigenvalue is equal to $-d_{\mathrm{mean}} + (S-1)\,E + \frac{1}{2E}\left(V+\rho-E^2\right) + \frac{1}{SE}\sigma_d^2$, and $S-1$ eigenvalues are contained in a line segment with left endpoint

$$\left(\lambda_{\mathbf{N}^*,2}\sqrt{\frac{1}{2}S\left(V+\rho-E^2\right)} - E, 0\right) \tag{143}$$

and right endpoint

$$\left(\lambda_{\mathbf{N}^*,1}\sqrt{\frac{1}{2}S\left(V+\rho-E^2\right)} - E, 0\right) \tag{144}$$

on the real axis.

Therefore, we can derive the expression of reactivity for community with mixed types of interactions:

When $|E| \le \sqrt{\left(V+\rho-E^2\right)/(2S) + \sigma_d^2/S^2}$:

$$\begin{aligned}\mathcal{R} &= \lambda_{\mathbf{N}^*,1}\sqrt{\frac{1}{2}S\left(V+\rho-E^2\right)} - E \\ &= \sqrt{\frac{1}{2}S\left(V+\rho-E^2\right)}\cdot \\ &\left(\frac{d_1+d_2}{2} + \sqrt{\left(\frac{d_2-d_1}{2}\right)^2+1} + \frac{2}{d_2-d_1}\tanh^{-1}\left(\frac{d_2-d_1}{\sqrt{\left(d_2-d_1\right)^2+4}}\right)\right) - E,\end{aligned} \tag{145}$$

where

$$\begin{cases} d_1 = \left(-d_{\mathrm{mean}} - \sqrt{3}\sigma_d\right)/\sqrt{S\cdot\mathrm{Var}\left(N_{\mathrm{inter},ij}\right)}, \\ d_2 = \left(-d_{\mathrm{mean}} + \sqrt{3}\sigma_d\right)/\sqrt{S\cdot\mathrm{Var}\left(N_{\mathrm{inter},ij}\right)}. \end{cases} \tag{146}$$

When $|E| > \sqrt{\left(V+\rho-E^2\right)/(2S) + \sigma_d^2/S^2}$:

$$\mathcal{R} = \max\left(\lambda_{\mathbf{N}^*,1}\sqrt{\frac{1}{2}S\left(V+\rho-E^2\right)} - E, \lambda_{\mathbf{H},\mathrm{outlier}}\right). \tag{147}$$

Clearly, reactivity of a community with mixed types of interactions is either

$$\begin{aligned}\mathcal{R} &= \sqrt{\frac{1}{2}S\left(V+\rho-E^2\right)}\cdot \\ &\left(\frac{d_1+d_2}{2} + \sqrt{\left(\frac{d_2-d_1}{2}\right)^2+1} + \frac{2}{d_2-d_1}\tanh^{-1}\left(\frac{d_2-d_1}{\sqrt{\left(d_2-d_1\right)^2+4}}\right)\right) - E,\end{aligned} \tag{148}$$

or

$$\mathcal{R} = -d_{\mathrm{mean}} + (S-1)\,E + \frac{1}{2E}\left(V + \rho - E^2\right) + \frac{1}{SE}\sigma_d^2. \tag{149}$$

If the reactivity expression has the form stated by Eq. (148), similar to the analysis in the random case, we can prove that the increase of self-regulation heterogeneity makes the system more reactive. If the reactivity expression has the form stated by Eq. (149), it is also clear that the increase of self-regulation heterogeneity makes the system more reactive. Thus, just as the random case, for community with mixed interaction types, the increase of self-regulation heterogeneity makes the system more reactive.

Note that for communities with mixed types of interactions, the derivation of theoretical expression of reactivity stands on the assumption that $\mathbf{H}'$ satisfies the requirements of the low-rank perturbation theorem. Therefore, the estimation of rightmost eigenvalue of matrix $\mathbf{H}$ may not be accurate for some extreme cases.

6.3 Incorporating non-self-regulating species into reactivity analysis

6.3.1 Random community

We still decompose the community matrix as stated previously. Here we consider the more general case, where S_n species self-regulate with strength d_{s}, $S - S_n$ species self-regulate with strength d_{e}. Clearly, the spectral density of $\mathbf{M}_{\mathrm{intra}}$ is

$$p\,(h) = (1-P)\,\delta\,(h + d_{\mathrm{s}}) + P\delta\,(h + d_{\mathrm{e}})\,, \tag{150}$$

where $P = 1 - S_n/S$, $\delta\,(\cdot)$ is the Dirac delta function.

Similar to cases with heterogeneous self-regulation strengths, we first construct a matrix $\mathbf{N} = \mathbf{H}/\sqrt{S \cdot \mathrm{Var}\,(H_{\mathrm{inter},ij})}$, which can also be expressed as

$$\mathbf{N} = \mathbf{N}_{\mathrm{inter}} + \mathbf{N}_{\mathrm{intra}}, \tag{151}$$

where $\mathbf{N}_{\mathrm{inter}} = \mathbf{H}_{\mathrm{inter}}/\sqrt{S \cdot \mathrm{Var}\,(H_{\mathrm{inter},ij})}$, $\mathbf{N}_{\mathrm{intra}} = \mathbf{H}_{\mathrm{intra}}/\sqrt{S \cdot \mathrm{Var}\,(H_{\mathrm{inter},ij})}$. We denote

$$\begin{cases} d_1 = -\frac{d_{\mathrm{e}}}{\sqrt{S \cdot \mathrm{Var}\left(H_{\mathrm{inter},ij}\right)}}, \\ d_2 = -\frac{d_{\mathrm{s}}}{\sqrt{S \cdot \mathrm{Var}\left(H_{\mathrm{inter},ij}\right)}}, \end{cases} \tag{152}$$

for simplicity, and this leads to the spectral density function of matrix $\mathbf{N}_{\text{intra}}$

$$p_{\mathbf{N}_{\text{intra}}}(h) = P\delta(h-d_1) + (1-P)\delta(h-d_2). \tag{153}$$

Since $\mathbf{N}_{\text{inter}}$ is now a standard elliptic matrix, the equations for the resolvent of matrix $\mathbf{N} = \mathbf{N}_{\text{inter}} + \mathbf{N}_{\text{intra}}$ can be derived

$$\begin{cases} 1 = \frac{P}{(x+\tau\alpha_{\text{re}}-d_1)^2+y^2/(1-\tau)^2+|\beta|^2} + \frac{1-P}{(x+\tau\alpha_{\text{re}}-d_2)^2+y^2/(1-\tau)^2+|\beta|^2}, \\ \alpha_{\text{re}} = -\frac{P(x+\tau\alpha_{\text{re}}-d_1)}{(x+\tau\alpha_{\text{re}}-d_1)^2+y^2/(1-\tau)^2+|\beta|^2} - \frac{(1-P)(x+\tau\alpha_{\text{re}}-d_2)}{(x+\tau\alpha_{\text{re}}-d_2)^2+y^2/(1-\tau)^2+|\beta|^2}, \end{cases} \tag{154}$$

where $x = \text{Re}(\lambda_{\mathbf{N}})$, $y = \text{Im}(\lambda_{\mathbf{N}})$. Again, since $\mathbf{N}$ is a symmetric matrix, equations above can be rewritten as

$$\begin{cases} 1 = \frac{P}{(x+\alpha_{\text{re}}-d_1)^2+|\beta|^2} + \frac{1-P}{(x+\alpha_{\text{re}}-d_2)^2+|\beta|^2}, \\ \alpha_{\text{re}} = -\frac{P(x+\alpha_{\text{re}}-d_1)}{(x+\alpha_{\text{re}}-d_1)^2+|\beta|^2} - \frac{(1-P)(x+\alpha_{\text{re}}-d_2)}{(x+\alpha_{\text{re}}-d_2)^2+|\beta|^2}. \end{cases} \tag{155}$$

When $|\beta| \to 0$, we can have

$$\begin{cases} 1 = \frac{P}{(x+\alpha_{\text{re}}-d_1)^2} + \frac{1-P}{(x+\alpha_{\text{re}}-d_2)^2}, \\ \alpha_{\text{re}} = -\frac{P}{x+\alpha_{\text{re}}-d_1} - \frac{(1-P)}{x+\alpha_{\text{re}}-d_2}. \end{cases} \tag{156}$$

Suppose $\tilde{x} = x - d_2 + \alpha_{\text{re}}$ and $\Delta = d_2 - d_1 > 0$, equations above become

$$\begin{cases} 1 = \frac{P}{(\tilde{x}+\Delta)^2} + \frac{1-P}{\tilde{x}^2}, \\ \alpha_{\text{re}} = -\frac{P}{\tilde{x}+\Delta} - \frac{1-P}{\tilde{x}}. \end{cases} \tag{157}$$

Solving these equations and find the largest root of $\tilde{x}$, we can obtain the rightmost eigenvalue of matrix $\mathbf{N}$ [3]

$$\lambda_{\mathbf{N},1} = \tilde{x}_1 + d_2 - \alpha_{\text{re}} = \tilde{x}_1 + d_2 + \frac{P}{\tilde{x}_1+\Delta} + \frac{1-P}{\tilde{x}_1}, \tag{158}$$

where

$$\begin{cases} \tilde{x}_1 = -\frac{\Delta}{2} + \frac{\sqrt{3}}{6}\sqrt{2\Delta^2 + \frac{6\sqrt{3}\Delta(1-2P)\sqrt{Q}}{\sqrt{\Delta^4+\Delta^2(Q-2)+(Q+1)^2}} - \frac{(\Delta^2-1)^2}{Q} - Q + 4 +} \\ \qquad \frac{\sqrt{3}}{6}\sqrt{\frac{\Delta^4+\Delta^2(Q-2)+(Q+1)^2}{Q}}, \\ Q = \left(\Delta^6 - 3\Delta^4 + \Delta^2\left(54P^2-54P+3\right) - 1\right. \\ \qquad \left. +6\sqrt{3}\Delta\sqrt{(P-1)P(\Delta^6-3\Delta^4+3\Delta^2(9P^2-9P+1)-1)}\right)^{\frac{1}{3}}. \end{cases} \tag{159}$$

Therefore, the rightmost eigenvalue of matrix $\mathbf{H}$ is (Supplemental Fig. S9)

$$\lambda_{\mathbf{H},1} = \frac{\sqrt{2}}{2}\sigma\sqrt{SC}\left(\tilde{x}_1 + d_2 + \frac{P}{\tilde{x}_1 + \Delta} + \frac{1-P}{\tilde{x}_1}\right), \tag{160}$$

and the expression of reactivity for random community where not all species self-regulate is

$$\mathcal{R} = \frac{\sqrt{2}}{2}\sigma\sqrt{SC}\left(\tilde{x}_1 + d_2 + \frac{P}{\tilde{x}_1 + \Delta} + \frac{1-P}{\tilde{x}_1}\right). \tag{161}$$

To study the influence of non-self-regulating species on system reactivity, we consider an extreme case where $d_{\mathrm{s}} = 0$, $d_{\mathrm{e}} = +\infty$. This leads to $d_1 = -\infty$ and $d_2 = 0$. The equations for resolvent (for matrix $\mathbf{N}$) become

$$\begin{cases} 1 = \frac{1-P}{\tilde{x}^2}, \\ \alpha_{\mathrm{re}} = -\frac{1-P}{\tilde{x}}, \end{cases} \tag{162}$$

and the expression of reactivity is now

$$\mathcal{R} = \sigma\sqrt{2SC\left(1-P\right)}. \tag{163}$$

Clearly, as long as $P < 1$, $\mathcal{R} > 0$, the system is reactive. This means that a single non-self-regulating species is enough to drive the system reactive, even if other species have infinitely strong self-regulation strength.

6.3.2 Community with mixed types of interactions

Similar to cases with heterogeneous self-regulation strengths, we still decompose the community matrix as $\mathbf{M} = \mathbf{M}_{\mathrm{inter}} + \mathbf{M}_{\mathrm{intra}}$, and the statistics of $\mathbf{M}_{\mathrm{inter}}$ are defined as stated previously. Matrix $\mathbf{H}$ can also be rewritten as

$$\begin{aligned} \mathbf{H} &= \left(\mathbf{H} - E\cdot\mathbf{1}\cdot\mathbf{1}^{\mathrm{T}} + E\mathbf{I}\right) + E\cdot\mathbf{1}\cdot\mathbf{1}^{\mathrm{T}} - E\mathbf{I} \\ &= \mathbf{N} + E\cdot\mathbf{1}\cdot\mathbf{1}^{\mathrm{T}} - E\mathbf{I}. \end{aligned} \tag{164}$$

We then consider matrix $\mathbf{N} = \mathbf{N}_{\mathrm{inter}} + \mathbf{N}_{\mathrm{intra}}$ first. To obtain the rightmost eigenvalue of matrix $\mathbf{N}$, we construct a new matrix $\mathbf{N}^* = \mathbf{N}/\sqrt{S\cdot\mathrm{Var}\left(N_{\mathrm{inter},ij}\right)}$. Clearly, $\mathbf{N}^*$ can also be written as $\mathbf{N}^* = \mathbf{N}^*_{\mathrm{inter}} + \mathbf{N}^*_{\mathrm{intra}}$. $\mathbf{N}^*_{\mathrm{inter}}$ is now a standard elliptic matrix, and the spectral density function of $\mathbf{N}^*_{\mathrm{intra}}$ is

$$p\left(h\right) = P\delta\left(h - d_1\right) + \left(1-P\right)\delta\left(h - d_2\right), \tag{165}$$

where

$$\begin{cases} d_1 = -\frac{d_{\mathrm{e}}}{\sqrt{S \cdot \mathrm{Var}\left(N_{\mathrm{inter},ij}\right)}}, \\ d_2 = -\frac{d_{\mathrm{s}}}{\sqrt{S \cdot \mathrm{Var}\left(N_{\mathrm{inter},ij}\right)}}. \end{cases} \tag{166}$$

Just as the random case, the rightmost eigenvalue of matrix $\mathbf{N}^*$ can be obtained as

$$\lambda_{\mathbf{N}^*,1} = \tilde{x}_1 + d_2 - \alpha_{\mathrm{re}} = \tilde{x}_1 + d_2 + \frac{P}{\tilde{x}_1 + \Delta} + \frac{1-P}{\tilde{x}_1}, \tag{167}$$

where

$$\begin{cases} \tilde{x}_1 = -\frac{\Delta}{2} + \frac{\sqrt{3}}{6}\sqrt{2\Delta^2 + \frac{6\sqrt{3}\Delta\left(1-2P\right)\sqrt{Q}}{\sqrt{\Delta^4 + \Delta^2\left(Q-2\right) + \left(Q+1\right)^2}} - \frac{\left(\Delta^2-1\right)^2}{Q} - Q + 4} + \\ \qquad \frac{\sqrt{3}}{6}\sqrt{\frac{\Delta^4 + \Delta^2\left(Q-2\right) + \left(Q+1\right)^2}{Q}}, \\ Q = \Big(\Delta^6 - 3\Delta^4 + \Delta^2\left(54P^2 - 54P + 3\right) - 1 \\ \qquad +6\sqrt{3}\Delta\sqrt{\left(P-1\right)P\left(\Delta^6 - 3\Delta^4 + 3\Delta^2\left(9P^2 - 9P + 1\right) - 1\right)}\Big)^{\frac{1}{3}}. \end{cases} \tag{168}$$

Thus, we can have $\lambda_{\mathbf{N}} = \lambda_{\mathbf{N}^*,1}\sqrt{\frac{1}{2}S\left(V + \rho - E^2\right)}$. We can then turn to matrix $\mathbf{H} = \mathbf{N} + E \cdot \mathbf{1} \cdot \mathbf{1}^{\mathrm{T}} - E \cdot \mathbf{I}$. To obtain its rightmost eigenvalue, we here consider matrix $\mathbf{H}' = \mathbf{H} + d_{\mathrm{mean}}\mathbf{I}$, where $d_{\mathrm{mean}} = \left(1-P\right)d_{\mathrm{s}} + Pd_{\mathrm{e}}$. We also rewrite $\mathbf{H}'$ as

$$\mathbf{H}' = \mathbf{N}' + E \cdot \mathbf{1} \cdot \mathbf{1}^{\mathrm{T}} - E \cdot \mathbf{I}, \tag{169}$$

where $\mathbf{N}' = \mathbf{N} + d_{\mathrm{mean}}\mathbf{I}$. Clearly, the rightmost eigenvalue of $\mathbf{N}'$ is

$$\lambda_{\mathbf{N}',1} = d_{\mathrm{mean}} + \lambda_{\mathbf{N}^*,1}\sqrt{\frac{1}{2}S\left(V + \rho - E^2\right)}, \tag{170}$$

and P proportion of diagonal terms of $\mathbf{N}'$ are $d_{\mathrm{mean}} - d_{\mathrm{e}}$, $1-P$ proportion of diagonal terms of $\mathbf{N}'$ are d_{mean}. The statistics of $\mathbf{N}'$ can be obtained as follows

$$\begin{cases} \mathbb{E}\left(N'_{ij}\right) = 0, \\ \mathrm{Var}\left(N'_{ij}\right) = \mathbb{E}\left(N'^2_{ij}\right) - \mathbb{E}^2\left(N'_{ij}\right). \end{cases} \tag{171}$$

Since

$$\mathbb{E}\left(N'^2_{ij}\right) = \frac{S\left(S-1\right)\mathbb{E}\left(N'^2_{ij}\right)_{i \neq j} + S\mathbb{E}\left(N'^2_{ij}\right)_{i=j}}{S^2}, \tag{172}$$

and

$$\begin{cases} \mathbb{E}\left(N_{ij}'^2\right)_{i\neq j} = \mathbb{E}\left(N_{\text{inter},ij}^2\right) = \text{Var}\left(N_{\text{inter},ij}\right) + \mathbb{E}^2\left(N_{\text{inter},ij}\right) = \frac{1}{2}\left(V+\rho+E^2\right), \\ \mathbb{E}\left(N_{ij}'^2\right)_{i=j} = P\left(d_{\text{mean}}-d_{\text{e}}\right)^2 + (1-P)\left(d_{\text{mean}}-d_{\text{s}}\right)^2. \end{cases} \tag{173}$$

For sufficiently large S, we have

$$\begin{aligned} \mathbb{E}\left(N_{ij}'^2\right) &= \frac{1}{2}\left(V+\rho-E^2\right) + \frac{1}{S}\left(P\left(d_{\text{mean}}-d_{\text{e}}\right)^2 + (1-P)\left(d_{\text{mean}}-d_{\text{s}}\right)^2\right) \\ &= \frac{1}{2}\left(V+\rho-E^2\right) + \frac{1}{S}\left(P\left(d_{\text{mean}}-d_{\text{e}}\right)^2 + (1-P)\,d_{\text{mean}}^2\right). \end{aligned} \tag{174}$$

Thus,

$$\text{Var}\left(N_{ij}'\right) = \frac{1}{2}\left(V+\rho-E^2\right) + \frac{1}{S}\left(P\left(d_{\text{mean}}-d_{\text{e}}\right)^2 + (1-P)\,d_{\text{mean}}^2\right). \tag{175}$$

According to low-rank perturbation theorem (here we still assume the $\mathbf{H}'$ satisfies the low-rank perturbation theorem), we have

When $|E| \leq \sqrt{\text{Var}\left(N_{ij}'\right)/S}$:

$$\lambda_{\mathbf{H}',1} = d_{\text{mean}} + \lambda_{\mathbf{N}^*,1}\sqrt{\frac{1}{2}S\left(V+\rho-E^2\right)} - E. \tag{176}$$

When $|E| > \sqrt{\text{Var}\left(N_{ij}'\right)/S}$:

$$\begin{aligned} \lambda_{\mathbf{H}',1} = \max\Bigg(& d_{\text{mean}} + \lambda_{\mathbf{N}^*,1}\sqrt{\frac{1}{2}S\left(V+\rho-E^2\right)} - E, \\ & (S-1)\,E + \frac{1}{2E}\left(V+\rho-E^2\right) + \frac{1}{SE}\left(P\left(d_{\text{mean}}-d_{\text{e}}\right)^2 + (1-P)\,d_{\text{mean}}^2\right)\Bigg). \end{aligned} \tag{177}$$

Then, we can give the expression of reactivity for communities with complex interaction types where not all species self-regulate (Supplemental Fig. S9):

When $|E| \leq \sqrt{\text{Var}\left(N_{ij}'\right)/S}$:

$$\mathcal{R} = \lambda_{\mathbf{N}^*,1}\sqrt{\frac{1}{2}S\left(V+\rho-E^2\right)} - E. \tag{178}$$

When $|E| > \sqrt{\mathrm{Var}\left(N'_{ij}\right)/S}$:

$$\begin{aligned}\mathcal{R} = &\max\Bigg(\lambda_{\mathbf{N}^*,1}\sqrt{\frac{1}{2}S\left(V+\rho-E^2\right)} - E, -d_{\mathrm{mean}} + \\ &(S-1)E + \frac{1}{2E}\left(V+\rho-E^2\right) + \frac{1}{SE}\left(P\left(d_{\mathrm{mean}}-d_{\mathrm{e}}\right)^2 + (1-P)\,d_{\mathrm{mean}}^2\right)\Bigg).\end{aligned} \tag{179}$$

To evaluate the influence of non-self-regulating species on system reactivity, we can also consider the extreme case where $d_{\mathrm{e}} = +\infty$ (just as the random case), which leads to the conclusion that a single non-self-regulating species is enough to make the system reactive.

Note that for communities with mixed types of interactions, the derivation of theoretical expression of reactivity stands on the assumption that $\mathbf{H}'$ satisfies the requirements of the low-rank perturbation theorem. Therefore, the estimation of rightmost eigenvalue of matrix $\mathbf{H}$ may not be accurate for some extreme cases.

7 Supplementary Note 7: Correlation between measures of variability, reactivity and asymptotic stability

In the main text and Supplementary Fig. S11-S14, we study the performance of communities under frequent perturbations by regularly increasing or decreasing abundances of random-selected species, and the performance is quantified by calculating the proportion of communities experiencing species loss. These frequent perturbations can also be studied through perturbed community models. In such settings, variability, which depicts the tendency of a variable to change over time, is a good index to evaluate the performance of a given community. Variability can be an indicator of the risk that a community might go extinct or collapse under frequent perturbations - communities with higher variability have a higher probability to experience species loss or system collapse.

In this Supplementary Note, we extend our discussion to these perturbed community models and correlate the measure of stability, reactivity and variability. Specifically, we construct a perturbed linearised model to draw the relationship between stability, reactivity and variability.

Following work by Arnoldi *et al.* [23], the perturbed linearised model is constructed as

$$\frac{\mathrm{d}x_i\left(t\right)}{\mathrm{d}t} = \sum_{j=1}^{S} M_{ij} x_j\left(t\right) + \sigma_{\mathrm{p},i} \sqrt{X_i\left(t\right)}^{\alpha} \xi_i\left(t\right), \tag{180}$$

where $X_i\left(t\right)$ represents the abundance of species i at time t, $x_i\left(t\right)$ depicts the deviation from equilibrium abundance X_i^*, coefficients M_{ij} are elements of the community matrix and represent the effect that a small change in abundance of species j has on the abundance of species i, and $\sigma_{\mathrm{p},i} \sqrt{X_i\left(t\right)}^{\alpha} \xi_i\left(t\right)$ is the perturbation term. In the perturbation term, $\xi_i\left(t\right)$ denotes a standard white-noise source. α controls the type of perturbation. Setting $\alpha = 0$ incorporates purely exogenous perturbations, such as random removal or addition of individuals, into the model, and this type of perturbation is immigration type perturbation. Setting $\alpha = 1$ means individuals respond incoherently. Such incoherent responses includes demographic stochasticity resulting from the variation of birth and death events. Therefore, this type of perturbation is called demographic type perturbation. Setting $\alpha = 2$ means individuals of a given species respond in synchrony to a perturbation. This type of perturbation is called environmental type perturbation as fluctuations of environmental variables typically affect all individuals of a given species.

In this model, variability $\mathcal{V}$ of a community is defined as

$$\mathcal{V} = \frac{\sigma_{\text{out}}^2}{\sigma_{\text{in}}^2}, \tag{181}$$

where $\sigma_{\text{in}}^2 = (1/S)\sum_i \sigma_i^2$, $\sigma_{\text{out}}^2 = (1/S)\sum_i \text{Var}\left(X_i\left(t\right)\right)$. Correspondingly, invariability $\mathcal{I}$ can be constructed as

$$\mathcal{I} = \frac{1}{2\mathcal{V}}. \tag{182}$$

Since perturbations can be regarded as external factors, and stability and reactivity are two inherent properties of the community. Therefore, stability and reactivity are still obtained from the community matrix $\mathbf{M}$.

Preliminary numerical calculations (Supplemental Fig. S15) show that compared with non-reactive stable communities, the invariability of reactive stable communities is relatively low (correspondingly, the variability is relatively high). Further numerical calculations (Supplemental Fig. S16-S28) confirm this finding and further reveal that the increase of invariability (correspondingly, the decrease of variability) is coupled with the decrease of reactivity and the increase of stability. This indicates that reactive stable communities are more vulnerable to species loss and system collapse, which supports our claim that reactivity can be a better predictor of species extinctions than stability under frequent perturbations.

8 Supplementary Note 8: Species abundances and reactivity of complex ecosystems

So far, our work follows the tradition initiated by May and ignores the possible abundance distribution of species. That is, we directly model the community matrix $\mathbf{M}$. However, elements of the community matrix are determined by interaction strengths as well as species abundances. The role of species abundances in constructing the community matrix $\mathbf{M}$ can be clearly seen when considering the well-known gLV model

$$\frac{\mathrm{d}X_i(t)}{\mathrm{d}t} = X_i(t)\left(r_i - s_i X_i(t) + \sum_{j\neq i} A_{ij} X_j(t)\right), \tag{183}$$

where r_i is the intrinsic growth rate of species i, s_i is the per-capita growth rate of species i, A_{ij} is the per-capita effect of species j on species i. The potential feasible equilibrium can be obtained by solving following system of equations

$$r_i - s_i X_i^* + \sum_{j\neq i} A_{ij} X_j^* = 0. \tag{184}$$

Therefore, community matrix with respect to this equilibrium can be derived as

$$\mathbf{M} = \mathrm{diag}\left(\mathbf{X}^*\right)\mathbf{A}, \tag{185}$$

where $\mathrm{diag}\left(\mathbf{X}^*\right)$ is a diagonal matrix with equilibrium abundance vector $\mathbf{X}^*$ on its main diagonal. Under the gLV modelling framework, our previous discussion can be interpreted as communities having homogeneous equilibrium abundances. Therefore, the influence of species abundances on system reactivity is unknown.

The main goal of this Supplementary Note is to extend our previous discussion to incorporate heterogeneous species abundances into reactivity analysis of complex ecosystems. Based on the gLV model, we first consider a simple case with S species to study the influence of rare species. In this case, $S-1$ species have the same abundance while one species has a much smaller abundance. Secondly, we develop an approximation theory to estimate the reactivity for communities with heterogeneous species abundances. With this approximation theory, we then discuss the relationship between the reactivity of $\mathbf{A}$ and the reactivity of $\mathbf{M}$.

8.1 Rare species tend to control system reactivity

There exists studies showing that abundant species tend to govern short-term dynamics after pulse perturbations, and rare species tend to govern long-term dynamics. Recently, work by Arnoldi *et al.* studied the influence of rare species (such species can be denoted as 'satellite' species, and abundant species can be denoted as 'core' species) on system stability and reveal that rare species tend to control system stability [24]. We are then wondering whether system reactivity can also be controlled by satellite species.

To explore this problem, we perform numerical simulations on a set of gLV systems with community size S. In these systems, $S-1$ species are core species with unit equilibrium abundance, and one species is satellite species with equilibrium abundance X_s^* much smaller than 1 (i.e., $X_s^* \ll 1$). By randomly removing an abundant species and the satellite species respectively, we find that just as stability, rare species tend to control system reactivity (Supplemental Fig. S19). Specifically, before and after a random abundant species is removed, the system yields almost the same reactivity value (Supplemental Fig. S19). However, the system does yield extremely different reactivity values before and after the satellite species is removed (Supplemental Fig. S19). In contrast, rare species has little influence on the observable short-term dynamics (Supplemental Fig. S20). Note that here we use the average return rate over a certain interval $\mathcal{R}_t^{\mathrm{avg}}$ as a measure of short-term dynamics. For detailed information about $\mathcal{R}_t^{\mathrm{avg}}$, please refer to Ref [24].

The influence of satellite species can be understood from the perspective of eigenvalue distribution of matrix $\mathbf{H}$. Due to the extremely low abundance, satellite species introduces a very low eigenvalue of $\mathbf{H}$, and this eigenvalue controls system reactivity. When the satellite species is removed, this very low eigenvalue also disappears, making the system yields an extremely different reactivity value. However, the removal of an abundant species does not influence this very low eigenvalue, and thus has no influence on system reactivity. Therefore, for such systems, reactivity may only reflect the feature of the satellite species, and thus provide limited information of system dynamics.

8.2 Incorporating heterogeneous species abundances into reactivity analysis

8.2.1 Modelling framework

To incorporate species abundances into reactivity analysis, we now sample the equilibrium abundance of species i (i.e., X_i^*) from a random distribution with mean μ_X and variance σ_X. Interaction matrix $\mathbf{A}$ is constructed following the same method as constructing community matrix previously (see Methods). For simplicity, here we consider communities with homogeneous per-capita self-regulation strength

(i.e., $s_i = s$). Following this modelling framework, the community matrix now takes following form

$$\mathbf{M} = \begin{bmatrix} A_{11}X_1^* & A_{12}X_1^* & \cdots & A_{1S}X_1^* \\ A_{21}X_2^* & A_{22}X_2^* & \cdots & A_{2S}X_2^* \\ \vdots & \vdots & \ddots & \vdots \\ A_{S1}X_S^* & A_{S2}X_S^* & \cdots & A_{SS}X_S^* \end{bmatrix}. \tag{186}$$

8.2.2 Random community

To perform theoretical reactivity analysis, we use the rightmost eigenvalue of matrix $\mathbf{W} = \left(\mathbf{R} + \mathbf{R}^{\mathrm{T}}\right)/2$ to approximate the rightmost eigenvalue of matrix $\mathbf{H} = \left(\mathbf{M} + \mathbf{M}^{\mathrm{T}}\right)/2$ (this approximation is supported by numerical simulations, see analysis below). Matrix $\mathbf{R}$ takes following form

$$\mathbf{R} = \begin{bmatrix} A_{11}Ab_{11} & A_{12}Ab_{12} & \cdots & A_{1S}Ab_{1S} \\ A_{21}Ab_{21} & A_{22}Ab_{22} & \cdots & A_{2S}Ab_{2S} \\ \vdots & \vdots & \ddots & \vdots \\ A_{S1}Ab_{S1} & A_{S2}Ab_{S2} & \cdots & A_{SS}Ab_{SS} \end{bmatrix}, \tag{187}$$

where Ab_{ij} are sampled from a random distribution with mean μ_X and variance σ_X independently. Note that in following analysis, we focus on the cases where species abundances are sampled from the uniform distribution $U\left[\mu_X - \sqrt{3}\sigma_X, \mu_X + \sqrt{3}\sigma_X\right]$. Therefore, the diagonal elements of $\mathbf{R}$ are now sampled from a uniform distribution $U\left[-s\mu_X - \sqrt{3}s\sigma_X, -s\mu_X + \sqrt{3}s\sigma_X\right]$. By denoting $d_{\text{mean}} = s\mu_X$ and $\sigma_d = s\sigma_X$, this distribution can be equivalently written as $U\left[-d_{\text{mean}} - \sqrt{3}\sigma_d, -d_{\text{mean}} + \sqrt{3}\sigma_d\right]$.

For simplicity, we decompose $\mathbf{R}$ as $\mathbf{R} = \mathbf{R}_{\text{intra}} + \mathbf{R}_{\text{inter}}$, where $\mathbf{R}_{\text{intra}}$ is a diagonal matrix which extracts the diagonal elements of $\mathbf{R}$, and $\mathbf{R}_{\text{inter}}$ extracts off-diagonal elements of $\mathbf{R}$. Since

$$\text{Var}\left(A_{\text{inter},ij}Ab_{ij}\right) = \text{Var}\left(A_{\text{inter},ij}\right)\text{Var}\left(Ab_i\right) + \text{Var}\left(A_{\text{inter},ij}\right)\mathbb{E}^2\left(Ab_i\right) + \text{Var}\left(Ab\right)\mathbb{E}^2\left(A_{\text{inter},ij}\right), \tag{188}$$

the statistics of $\mathbf{R}_{\text{inter}}$ can be obtained as

$$\begin{cases} \mathbb{E}\left(R_{\text{inter},ij}\right) = \mathbb{E}\left(R_{\text{inter},ij}Ab_i\right) = 0, \\ \text{Var}\left(R_{\text{inter},ij}\right) = C\sigma^2\left(\mu_X^2 + \sigma_X^2\right), \\ \mathbb{E}\left(R_{\text{inter},ij}R_{\text{inter},ji}\right)_{i\neq j} = 0. \end{cases} \tag{189}$$

Since $\mathbf{W} = \left(\mathbf{R} + \mathbf{R}^{\mathrm{T}}\right)/2$, we have

$$\begin{cases} \mathbb{E}\left(W_{\text{inter},ij}\right) = 0, \\ \operatorname{Var}\left(W_{\text{inter},ij}\right) = \frac{1}{2}C\sigma^2\left(\mu_X^2 + \sigma_X^2\right), \\ \mathbb{E}\left(W_{\text{inter},ij}W_{\text{inter},ji}\right)_{i\neq j} = \frac{1}{2}C\sigma^2\left(\mu_X^2 + \sigma_X^2\right), \end{cases} \tag{190}$$

and diagonal entries of $\mathbf{W}_{\text{intra}}$ are sampled from a uniform distribution

$$U\left[-d_{\text{mean}} - \sqrt{3}\sigma_d, -d_{\text{mean}} + \sqrt{3}\sigma_d\right], \tag{191}$$

where $d_{\text{mean}} = s\mu_X,\ \sigma_d = s\sigma_X$. It is clear that matrix $\mathbf{W}$ is now a random matrix with heterogeneous diagonal elements. Therefore, we can now utilize the same method used in Supplementary Note 6 and the work by Barabás *et al.* [3] to derive the eigenvalue distribution of matrix $\mathbf{W}$.

Similarly, we first construct matrix $\mathbf{N} = \mathbf{W}/\sqrt{S\cdot\operatorname{Var}\left(W_{\text{inter},ij}\right)}$. Matrix $\mathbf{N}$ can also be decomposed as

$$\mathbf{N} = \mathbf{N}_{\text{intra}} + \mathbf{N}_{\text{inter}}. \tag{192}$$

Thus, we have

$$\begin{cases} \mathbb{E}\left(N_{\text{inter},ij}\right) = 0, \\ \operatorname{Var}\left(N_{\text{inter},ij}\right) = \frac{1}{S}, \\ \mathbb{E}\left(N_{\text{inter},ij}N_{\text{inter},ji}\right)_{i\neq j} = \frac{1}{S}. \end{cases} \tag{193}$$

and diagonal entries of $\mathbf{N}_{\text{intra}}$ are now sampled from a uniform distribution

$$U\left[\left(-d_{\text{mean}} - \sqrt{3}\sigma_d\right)/\sqrt{S\cdot\operatorname{Var}\left(W_{\text{inter},ij}\right)}, \left(-d_{\text{mean}} + \sqrt{3}\sigma_d\right)/\sqrt{S\cdot\operatorname{Var}\left(W_{\text{inter},ij}\right)}\right], \tag{194}$$

where $d_{\text{mean}} = s\mu_X, \sigma_d = s\sigma_X$.

For simplicity, we denote

$$\begin{cases} d_1 = \left(-d_{\text{mean}} - \sqrt{3}\sigma_d\right)/\sqrt{S\cdot\operatorname{Var}\left(W_{\text{inter},ij}\right)}, \\ d_2 = \left(-d_{\text{mean}} + \sqrt{3}\sigma_d\right)/\sqrt{S\cdot\operatorname{Var}\left(W_{\text{inter},ij}\right)}. \end{cases} \tag{195}$$

Analyses in Supplementary Note 6 and the work by Barabás *et al.* [3] show that the eigenvalues of matrix $\mathbf{N}$ are now distributed in a line segment with left endpoint $(\lambda_{\mathbf{N},2}, 0)$ and right endpoint

$(\lambda_{\mathbf{N},1}, 0)$ on the real axis. Here

$$\begin{cases} \lambda_{\mathbf{N},1} = \frac{d_1+d_2}{2} + \sqrt{\left(\frac{d_2-d_1}{2}\right)^2 + 1} + \frac{2}{d_2-d_1} \tanh^{-1}\left(\frac{d_2-d_1}{\sqrt{(d_2-d_1)^2+4}}\right), \\ \lambda_{\mathbf{N},2} = \frac{d_1+d_2}{2} - \sqrt{\left(\frac{d_2-d_1}{2}\right)^2 + 1} - \frac{2}{d_2-d_1} \tanh^{-1}\left(\frac{d_2-d_1}{\sqrt{(d_2-d_1)^2+4}}\right). \end{cases} \tag{196}$$

Eigenvalues of $\mathbf{W}$ are thus contained in a line segment with left endpoint $\left(\lambda_{\mathbf{N},2}\sqrt{\frac{1}{2}SC\sigma^2\left(\mu_X^2+\sigma_X^2\right)}, 0\right)$ and right endpoint $\left(\lambda_{\mathbf{N},1}\sqrt{\frac{1}{2}SC\sigma^2\left(\mu_X^2+\sigma_X^2\right)}, 0\right)$ on the real axis.

Numerical simulations show that the rightmost eigenvalue of $\mathbf{H}$ is in good agreement with the rightmost eigenvalue of $\mathbf{W}$ (Supplemental Fig. S21), which suggests that it is appropriate to use $\lambda_{\mathbf{W},1}$ to approximate $\lambda_{\mathbf{W},1}$. Thus, the approximated expression of reactivity for a random community can be obtained as

$$\mathcal{R} = \frac{\sqrt{2}}{2}\sigma\sqrt{SC\left(\mu_X^2+\sigma_X^2\right)}\left(\frac{d_1+d_2}{2} + \sqrt{\left(\frac{d_2-d_1}{2}\right)^2+1} + \frac{2}{d_2-d_1}\tanh^{-1}\left(\frac{d_2-d_1}{\sqrt{(d_2-d_1)^2+4}}\right)\right), \tag{197}$$

where

$$\begin{cases} d_1 = \left(-\sqrt{2}s\mu_X - \sqrt{6}s\sigma_X\right)/\sigma\sqrt{SC\left(\mu_X^2+\sigma_X^2\right)}, \\ d_2 = \left(-\sqrt{2}s\mu_X + \sqrt{6}s\sigma_X\right)/\sigma\sqrt{SC\left(\mu_X^2+\sigma_X^2\right)}. \end{cases} \tag{198}$$

Further numerical simulations also prove the effectiveness of our approximation method (Supplemental Fig. S22).

Clearly, $d_2 - d_1 = 2\sqrt{6}s\sigma_X/\sigma\sqrt{SC\left(\mu_X^2+\sigma_X^2\right)}$, we can rewrite expression above as

$$\begin{aligned} \mathcal{R} = & -s\mu_X + \sqrt{12s^2\sigma_X^2 + \frac{1}{2}\sigma^2 SC\left(\mu_X^2+\sigma_X^2\right)} \\ & + \frac{\sigma^2 SC\left(\mu_X^2+\sigma_X^2\right)}{2\sqrt{3}s\sigma_X}\tanh^{-1}\left(\frac{\sqrt{6}s\sigma_X}{\sqrt{6s^2\sigma_X^2+\sigma^2 SC\left(\mu_X^2+\sigma_X^2\right)}}\right). \end{aligned} \tag{199}$$

Please note that what we provide is an approximation theory. Therefore, under some conditions (e.g., the variance of species abundances is relatively high), our theory may be unable to give an accurate prediction of the reactivity.

8.2.3 Community with mixed types of interactions

As random case, we still use the rightmost eigenvalue of matrix $\mathbf{W} = \left(\mathbf{R}+\mathbf{R}^{\mathrm{T}}\right)/2$ to approximate the rightmost eigenvalue of matrix $\mathbf{H} = \left(\mathbf{M}+\mathbf{M}^{\mathrm{T}}\right)/2$. We still focus on the case where species

abundances are sampled from a uniform distribution $U\left[\mu_X - \sqrt{3}\sigma_X, \mu_X + \sqrt{3}\sigma_X\right]$.

Decompose $\mathbf{R}$ as $\mathbf{R} = \mathbf{R}_{\text{intra}} + \mathbf{R}_{\text{inter}}$, we have

$$\begin{cases} \mathbb{E}\left(R_{\text{inter},ij}\right) = C\mathbb{E}\left(|Z|\right)\left(P_{+/+} - P_{-/-}\right)\mu_X = E\mu_X = E_R, \\ \text{Var}\left(R_{\text{inter},ij}\right) = \left(C\sigma^2 - E^2\right)\sigma_X^2 + \left(C\sigma^2 - E^2\right)\mu_X^2 + \sigma_X^2 E^2 \mu_X^2 = V_R, \\ \mathbb{E}\left(R_{\text{inter},ij}R_{\text{inter},ji}\right)_{i\neq j} = C\mathbb{E}^2\left(|Z|\right)\left(P_{+/+} + P_{-/-} - P_{+/-}\right)\mu_X^2 = \rho\mu_X^2 = \rho_R, \end{cases} \tag{200}$$

and diagonal elements of $\mathbf{R}_{\text{intra}}$ are now sampled from a uniform distribution

$$U\left[-d_{\text{mean}} - \sqrt{3}\sigma_d, -d_{\text{mean}} + \sqrt{3}\sigma_d\right], \tag{201}$$

where $d_{\text{mean}} = s\mu_X,\ \sigma_d = s\sigma_X$.

Since $\mathbf{W} = \left(\mathbf{R} + \mathbf{R}^{\text{T}}\right)/2$, we can then have

$$\begin{cases} \mathbb{E}\left(W_{\text{inter},ij}\right) = E_R, \\ \text{Var}\left(W_{\text{inter},ij}\right) = \frac{1}{2}\left(V_R + \rho_R - E_R^2\right), \\ \mathbb{E}\left(W_{\text{inter},ij}W_{\text{inter},ji}\right)_{i\neq j} = \frac{1}{2}\left(V_R + \rho_R + E_R^2\right). \end{cases} \tag{202}$$

and diagonal elements of $\mathbf{R}_{\text{intra}}$ are sampled from a uniform distribution

$$U\left[-d_{\text{mean}} - \sqrt{3}\sigma_d, -d_{\text{mean}} + \sqrt{3}\sigma_d\right], \tag{203}$$

where $d_{\text{mean}} = s\mu_X,\ \sigma_d = s\sigma_X$.

To locate the rightmost eigenvalue of $\mathbf{W}$, we rewrite $\mathbf{W}$ as

$$\begin{aligned} \mathbf{W} &= \left(\mathbf{W} - E_R \cdot \mathbf{1} \cdot \mathbf{1}^{\text{T}} + E_R\mathbf{I}\right) + E_R \cdot \mathbf{1} \cdot \mathbf{1}^{\text{T}} - E_R\mathbf{I} \\ &= \mathbf{N} + E_R \cdot \mathbf{1} \cdot \mathbf{1}^{\text{T}} - E_R\mathbf{I}, \end{aligned} \tag{204}$$

where $\mathbf{1} \cdot \mathbf{1}^{\text{T}}$ is a $S \times S$ matrix with each element equaling 1. $\mathbf{N}$ can also be decomposed as

$$\mathbf{N} = \mathbf{N}_{\text{intra}} + \mathbf{N}_{\text{inter}}, \tag{205}$$

where $\mathbf{N}_{\text{inter}} = \mathbf{W}_{\text{inter}} - E_R \cdot \mathbf{1} \cdot \mathbf{1}^{\text{T}} + E_R\mathbf{I}$, $\mathbf{N}_{\text{intra}} = \mathbf{W}_{\text{intra}}$. Thus, the statistics of matrix $\mathbf{N}_{\text{inter}}$ can

be drawn as

$$\begin{cases} \mathbb{E}\left(N_{\text{inter},ij}\right)=0, \\ \text{Var}\left(N_{\text{inter},ij}\right)=\frac{1}{2}\left(V_R+\rho_R-E_R^2\right), \\ \mathbb{E}\left(N_{\text{inter},ij}N_{\text{inter},ji}\right)_{i\neq j}=\frac{1}{2}\left(V_R+\rho_R-E_R^2\right). \end{cases} \tag{206}$$

We then construct matrix

$$\mathbf{N}^*=\frac{\mathbf{N}}{\sqrt{S\cdot\text{Var}\left(N_{\text{inter},ij}\right)}}. \tag{207}$$

For simplicity, we also denote

$$\begin{cases} d_1=\left(-d_{\text{mean}}-\sqrt{3}\sigma_d\right)/\sqrt{S\cdot\text{Var}\left(N_{\text{inter},ij}\right)}, \\ d_2=\left(-d_{\text{mean}}+\sqrt{3}\sigma_d\right)/\sqrt{S\cdot\text{Var}\left(N_{\text{inter},ij}\right)}. \end{cases} \tag{208}$$

Analyses in Supplementary Note 6 and the work by Barabás *et al.* [3] show that eigenvalues of $\mathbf{N}^*$ are distributed in a line segment with left endpoint $(\lambda_{\mathbf{N}^*,2},0)$ and right endpoint $(\lambda_{\mathbf{N}^*,1},0)$. Here

$$\begin{cases} \lambda_{\mathbf{N}^*,1}=\frac{d_1+d_2}{2}+\sqrt{\left(\frac{d_2-d_1}{2}\right)^2+1}+\frac{2}{d_2-d_1}\tanh^{-1}\left(\frac{d_2-d_1}{\sqrt{(d_2-d_1)^2+4}}\right), \\ \lambda_{\mathbf{N}^*,2}=\frac{d_1+d_2}{2}-\sqrt{\left(\frac{d_2-d_1}{2}\right)^2+1}-\frac{2}{d_2-d_1}\tanh^{-1}\left(\frac{d_2-d_1}{\sqrt{(d_2-d_1)^2+4}}\right). \end{cases} \tag{209}$$

This further leads to the rightmost eigenvalue of $\mathbf{W}$:
When $|E_R|\leq\sqrt{\left(V_R+\rho_R-E_R^2\right)/(2S)+\left(s^2\sigma_X^2\right)/S^2}$:

$$\lambda_{\mathbf{W},1}=\lambda_{\mathbf{N}^*,1}\sqrt{\frac{1}{2}S\left(V_R+\rho_R-E_R^2\right)}-E_R. \tag{210}$$

When $|E_R|>\sqrt{\left(V_R+\rho_R-E_R^2\right)/(2S)+\left(s^2\sigma_X^2\right)/S^2}$:

$$\lambda_{\mathbf{W},1}= \\ \max\left(\lambda_{\mathbf{N}^*,1}\sqrt{\frac{1}{2}S\left(V_R+\rho_R-E_R^2\right)}-E_R,-s\mu_X+(S-1)E_R+\frac{1}{2E_R}\left(V_R+\rho_R-E_R^2\right)+\frac{1}{SE}s^2\sigma_X^2\right). \tag{211}$$

Numerical simulations show that the rightmost eigenvalue of $\mathbf{H}$ is in good agreement with the rightmost eigenvalue of $\mathbf{W}$ (Supplemental Fig. S21), which suggests that it is appropriate to use $\lambda_{\mathbf{W},1}$ to approximate $\lambda_{\mathbf{W},1}$. Thus, the approximated expression of reactivity for community with mixed

types of interactions is either

$$\mathcal{R} = \lambda_{\mathbf{N}^*,1}\sqrt{\frac{1}{2}S\left(V_R+\rho_R-E_R^2\right)}-E_R$$
$$= \sqrt{\frac{1}{2}S\left(V_R+\rho_R-E_R^2\right)}\left(\frac{d_1+d_2}{2}+\sqrt{\left(\frac{d_2-d_1}{2}\right)^2+1}+\frac{2}{d_2-d_1}\tanh^{-1}\left(\frac{d_2-d_1}{\sqrt{\left(d_2-d_1\right)^2+4}}\right)\right)-E_R, \tag{212}$$

or

$$\mathcal{R} = -s\mu_X+\left(S-1\right)E_R+\frac{1}{2E_R}\left(V_R+\rho_R-E_R^2\right)+\frac{1}{SE}s^2\sigma_X^2. \tag{213}$$

Further numerical simulations also prove the effectiveness of our approximation method (Supplemental Fig. S22).

Please note that what we provide is an approximation theory. Therefore, under some conditions (e.g., the variance of species abundances is relatively high), our theory may be unable to give an accurate prediction of the reactivity.

8.3 The relationship between the reactivity of A and the reactivity of M

8.3.1 Random community

In this section, we discuss the relationship between the reactivity of $\mathbf{M}$ and the reactivity of $\mathbf{A}$ for random communities. To do so, we first discuss the simplest case where all species have the same equilibrium abundance. In this case, the approximated reactivity expression is an accurate expression and it is

$$\mathcal{R} = \mu_X\left(-s+\sigma\sqrt{2SC}\right). \tag{214}$$

Clearly, in such case, change of species abundances does not influence system reactivity. If $\mathbf{A}$ is reactive (non-reactive), then $\mathbf{M}$ is reactive (non-reactive).

We now extend the discussion to more general cases when species abundances are heterogeneous. We first consider the case where $\mathbf{A}$ is reactive. That is, $\lambda_{\frac{\mathbf{A}+\mathbf{A}^T}{2},1}>0$. Based on our previous results, this is equivalent to $s<\sigma\sqrt{2SC}$. According to the approximated expression of reactivity for random communities with species abundances sampled from a uniform distribution, we can see that whether

the community is reactive or not is determined by the sign of following function

$$
\begin{aligned}
f\left(d_1, d_2\right) &= \frac{d_1+d_2}{2}+\sqrt{\left(\frac{d_2-d_1}{2}\right)^2+1}+\frac{2}{d_2-d_1} \tanh^{-1}\left(\frac{d_2-d_1}{\sqrt{\left(d_2-d_1\right)^2+4}}\right) \\
&= \frac{-\sqrt{2} s \mu_X}{\sqrt{\sigma^2 S C\left(\mu_X^2+\sigma_X^2\right)}}+\sqrt{\left(\frac{d_2-d_1}{2}\right)^2+1}+\frac{2}{d_2-d_1} \tanh^{-1}\left(\frac{d_2-d_1}{\sqrt{\left(d_2-d_1\right)^2+4}}\right) \\
&= \frac{-\sqrt{2} s \mu_X}{\sqrt{\sigma^2 S C\left(\mu_X^2+\sigma_X^2\right)}}+g\left(d_2-d_1\right).
\end{aligned}
\tag{215}
$$

If $f\left(d_1, d_2\right)<0$, i.e.,

$$
s>g\left(d_2-d_1\right) \sqrt{\frac{1}{2} \sigma^2 S C\left(1+\frac{\sigma_X^2}{\mu_X^2}\right)}, \tag{216}
$$

then the community is (approximately) non-reactive. Analysis in Supplementary Note 6 shows that as long as $d_2-d_1>0$ (i.e., species abundances are heterogeneous), $g(d_2-d_1)>g(0)=2$. Thus, to ensure non-reactivity, following inequality must hold

$$
s>\sqrt{2 \sigma^2 S C\left(1+\frac{\sigma_X^2}{\mu_X^2}\right)}. \tag{217}
$$

However, this contradicts our premise that $\mathbf{A}$ is reactive. Therefore, when $\mathbf{A}$ is reactive, $\mathbf{M}$ is (approximately) reactive.

When $\mathbf{A}$ is non-reactive, we have $s>\sigma \sqrt{2 S C}$. Since

$$
g\left(d_2-d_1\right) \sqrt{\frac{1}{2} \sigma^2 S C\left(1+\frac{\sigma_X^2}{\mu_X^2}\right)}>\sqrt{2 \sigma^2 S C\left(1+\frac{\sigma_X^2}{\mu_X^2}\right)}>\sigma \sqrt{2 S C}, \tag{218}
$$

with different s, $\mathbf{M}$ can be reactive or non-reactive. Specifically, $\sigma \sqrt{2 S C}<s<g\left(d_2-d_1\right) \cdot \sqrt{\frac{1}{2} \sigma^2 S C\left(1+\frac{\sigma_X^2}{\mu_X^2}\right)}$ can lead to a reactive $\mathbf{M}$, while $s>g\left(d_2-d_1\right) \sqrt{\frac{1}{2} \sigma^2 S C\left(1+\frac{\sigma_X^2}{\mu_X^2}\right)}$ can lead to a non-reactive $\mathbf{M}$.

8.3.2 Community with mixed types of interactions

Like random case, we first consider the case with homogeneous equilibrium abundance. According to our previous results, the accurate expression of reactivity is either

$$
\mathcal{R}=\mu_X\left(-s-E+\sqrt{2 S\left(V+\rho-E^2\right)}\right), \tag{219}
$$

or

$$\mathcal{R} = \mu_X \left(-s + (S-1)\, E\right). \tag{220}$$

Clearly, change of species abundances in such case does not influence system reactivity. If **A** is reactive (non-reactive), **M** is reactive (non-reactive).

We then discuss the case where species abundances are sampled from a uniform distribution. We first consider the case when the reactivity of **A** and **M** are both determined by the outlier. In this case, when **A** is reactive, we have

$$s < (S-1)\, E. \tag{221}$$

If the corresponding **M** is non-reactive, following inequality should hold

$$s\mu_X > (S-1)\, E_R + \frac{1}{2E_R}\left(V_R + \rho_R - E_R^2\right) + \frac{1}{SE} s^2 \sigma_X^2. \tag{222}$$

This inequality contradicts $s < (S-1)\, E$. Therefore, when system reactivity is determined by the outlier, reactivity of **A** implies the reactivity of **M**. When **A** is non-reactive (i.e., $s > (S-1)\, E$), depending on the choose of s, **M** can be reactive ($(S-1)\, E\mu_X < s\mu_X < (S-1)\, E_R + \frac{1}{2E_R}\left(V_R + \rho_R - E_R^2\right) + \frac{1}{SE} s^2 \sigma_X^2$) or non-reactive ($s\mu_X > (S-1)\, E_R + \frac{1}{2E_R}\left(V_R + \rho_R - E_R^2\right) + \frac{1}{SE} s^2 \sigma_X^2$).

We then turn to the case where the reactivity of **A** and **M** are both determined by the right endpoint of bulk eigenvalues. When **A** is reactive, we have

$$s < \sqrt{2S\left(V + \rho - E^2\right)} - E. \tag{223}$$

To ensure the non-reactivity of corresponding **M**, following inequality must hold

$$\sqrt{\frac{1}{2} S\left(V_R + \rho_R - E_R^2\right)} \left(\frac{d_1 + d_2}{2} + \sqrt{\left(\frac{d_2 - d_1}{2}\right)^2 + 1} + \frac{2}{d_2 - d_1} \tanh^{-1}\left(\frac{d_2 - d_1}{\sqrt{\left(d_2 - d_1\right)^2 + 4}} \right) \right) - E_R < 0. \tag{224}$$

This inequality is equivalent to

$$s > \sqrt{2S\left(V\left(1 + \frac{\sigma_X^2}{\mu_X^2}\right) + \rho - E^2 + \sigma_X^2 E^2\right)} - E, \tag{225}$$

which contradicts $s < \sqrt{2S\left(V + \rho - E^2\right)} - E$. Therefore, the reactivity of **A** implies the reactivity of **M**. Similar to analysis before, we can also show that the non-reactivity of **A** does not imply the non-reactivity of **M**.

Based on discussions above, the case when the reactivity of $\mathbf{A}$ and $\mathbf{M}$ are determined by different eigenvalues (i.e., one is determined by the outlier, and the other is determined by the rightmost eigenvalue of bulk eigenvalues) can also be studied and lead to the same results above. Therefore, we can then arrive the conclusion that for communities with species abundances sampled from a uniform distribution, reactivity of $\mathbf{A}$ implies the reactivity of $\mathbf{M}$. Our analysis method may also be extended to cases where species abundances are sampled from other distributions. We then perform numerical simulations, and simulation results show the same pattern as predicted by theory (Supplemental Fig. S23). Since our numerical simulations are not limited to sample species abundances from uniform distributions, the conclusion drawn above may be a universal conclusion. However, please note that here we are only providing an approximation theory, more accurate theory is still in need.

Details about simulation strategy are provided as follows. In the simulations, community sizes (i.e., S) are sampled from a uniform distribution $U[50, 300]$ (note that community sizes should be integers), connectances (i.e., C) are sampled from a uniform distribution $U[0, 1]$, standard deviation of interspecies interactions (i.e., σ) are sampled from a uniform distribution $U[0, 0.5]$, and per-capita self-regulation strengths (i.e., s) are sampled from a uniform distribution $U[0, 5]$ (note that all species have the same s). For communities with species abundances sampled from a uniform distribution, means of species abundances (i.e., μ_X) are sampled from a uniform distribution $U[0.5, 5]$, standard deviations of species abundances (i.e., σ_X) are sampled from a uniform distribution $U\left[0, \mu_X/\sqrt{3}\right]$. For communities with species abundances sampled from a gamma distribution, means of species abundances (i.e., μ_X) are sampled from a uniform distribution $U[0.5, 5]$, standard deviations of species abundances (i.e., σ_X) are sampled from a uniform distribution $U[0.5, 5]$. For communities with species abundances sampled from a log-normal distribution, means of species abundances (i.e., μ_X) are sampled from a uniform distribution $U[0.5, 5]$, standard deviations of species abundances (i.e., σ_X) are sampled from a uniform distribution $U[0.5, 5]$. For communities with species abundances sampled from a half-normal distribution (more precisely, species abundances take the value of $|Z|$, Z is a random variable sampled from a normal distribution with mean 0 and variance σ_z^2), means of species abundances (i.e., μ_X) are sampled from a uniform distribution $U[0.5, 5]$.

Supplementary Figures

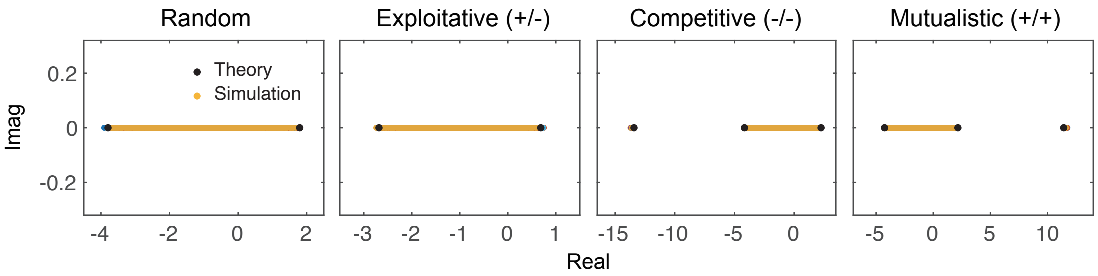

Supplemental Figure S1: Eigenvalue distributions of four classic types of communities. Colored dots are eigenvalues of $\mathbf{H}$ of 10 randomly generated communities. Black dots are theoretical predications of the endpoints of the bulk of eigenvalues and the outlier. In this figure, $S = 250,\ C = 0.25,\ \sigma = 0.25$ and $d = 1$.

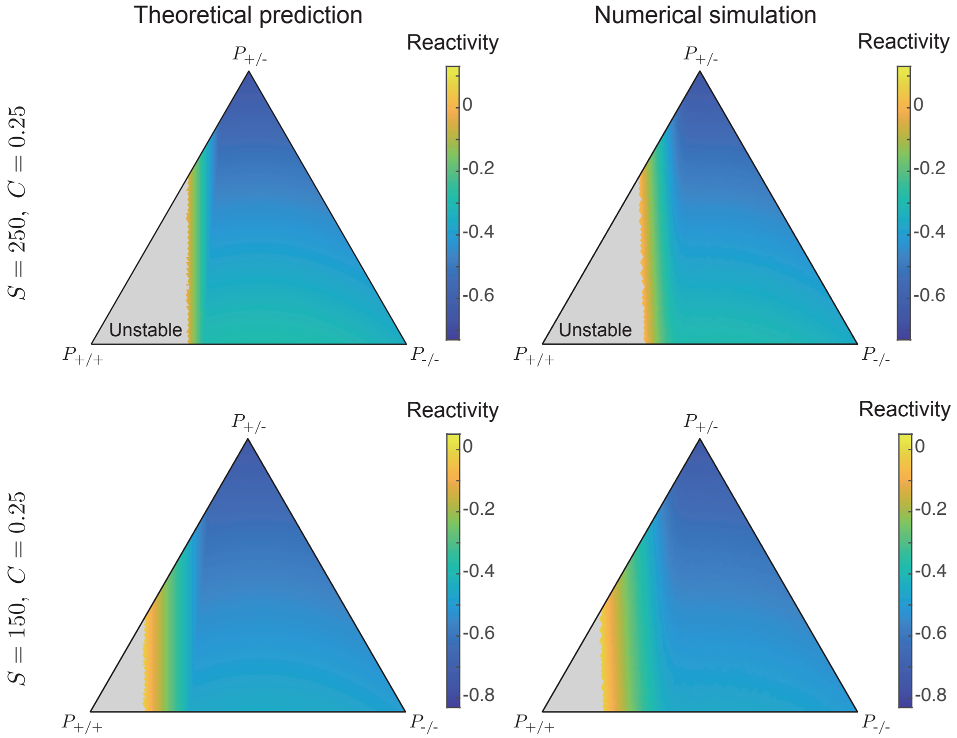

Supplemental Figure S2: Reactivity of communities with mixed interactions of exploitation (+/-), mutualism (+/+) and competition (-/-). Left row shows the results from theoretical prediction, and right row presents the results of numerical simulation. Each data point in the right row is an average of 50 randomly generated communities with the same set of parameters. Colors closer to yellow refer to higher reactivity, and colors closer to blue represent lower reactivity. Other parameters in this figure are $d = 1, \sigma = 0.05$.

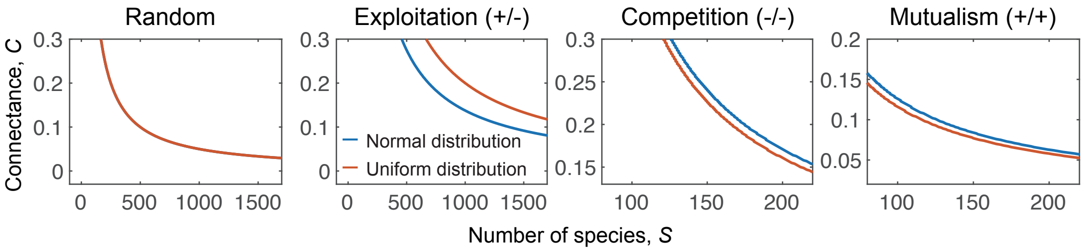

Supplemental Figure S3: Reactivity criteria under different interaction strength distributions. Blue lines are reactivity criteria when interaction strengths are sampled from a normal distribution (i.e., $Z \sim N(0, \sigma^2)$). Red lines are reactivity criteria when interaction strengths are sampled from a uniform distribution (i.e., $Z \sim U\left[-\sqrt{3}\sigma, \sqrt{3}\sigma\right]$). In this figure, we have $\sigma = 0.1,\ d = 1$.

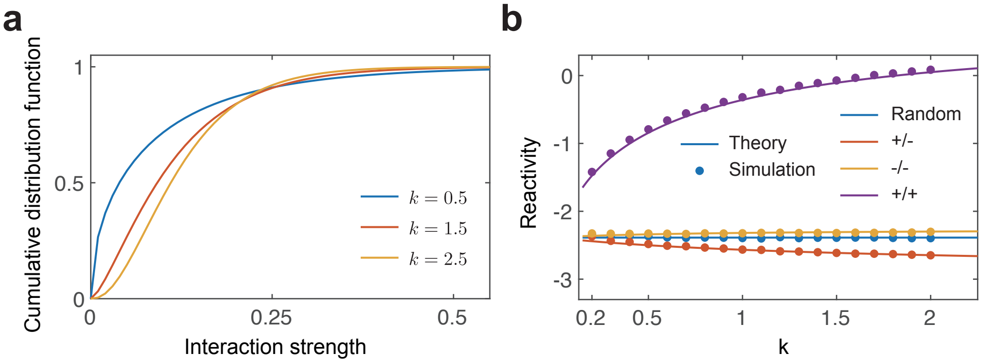

Supplemental Figure S4: Reactivity of communities under Gamma distribution. In this figure, positive interaction strengths are sampled from $Z \sim \Gamma(k, \sigma\sqrt{1/\left(k^2+k\right)})$, and negative interaction strengths are sampled from $Z \sim -\Gamma(k, \sigma\sqrt{1/\left(k^2+k\right)})$. Panel **a** shows the cumulative distribution function for different k. It is clear that as k increases, the proportion of weak interactions decreases. Panel **b** shows the reactivity of different types of communities when k changes. In this figure, $S = 250,\ C = 0.3,\ \sigma = 0.05$ and $d = 3$.

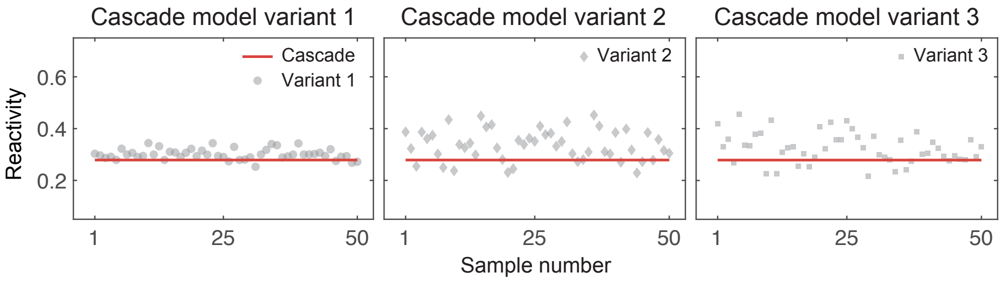

Supplemental Figure S5: Reactivity of different variants of cascade model. Variant 1 is interval cascade food web, variant 2 is cascade food web with broad degree distribution, and variant 3 is interval cascade food web with broad degree distribution. Red lines represent the reactivity of the original cascade model, grey symbols represent the reactivity values of different variants of cascade model. For each type of variant, we randomly generate 50 communities with the same community parameters combination and plot their reactivity. In this figure, we have $S = 500,\ C = 0.2,\ \sigma = 0.15$ and $d = 1$.

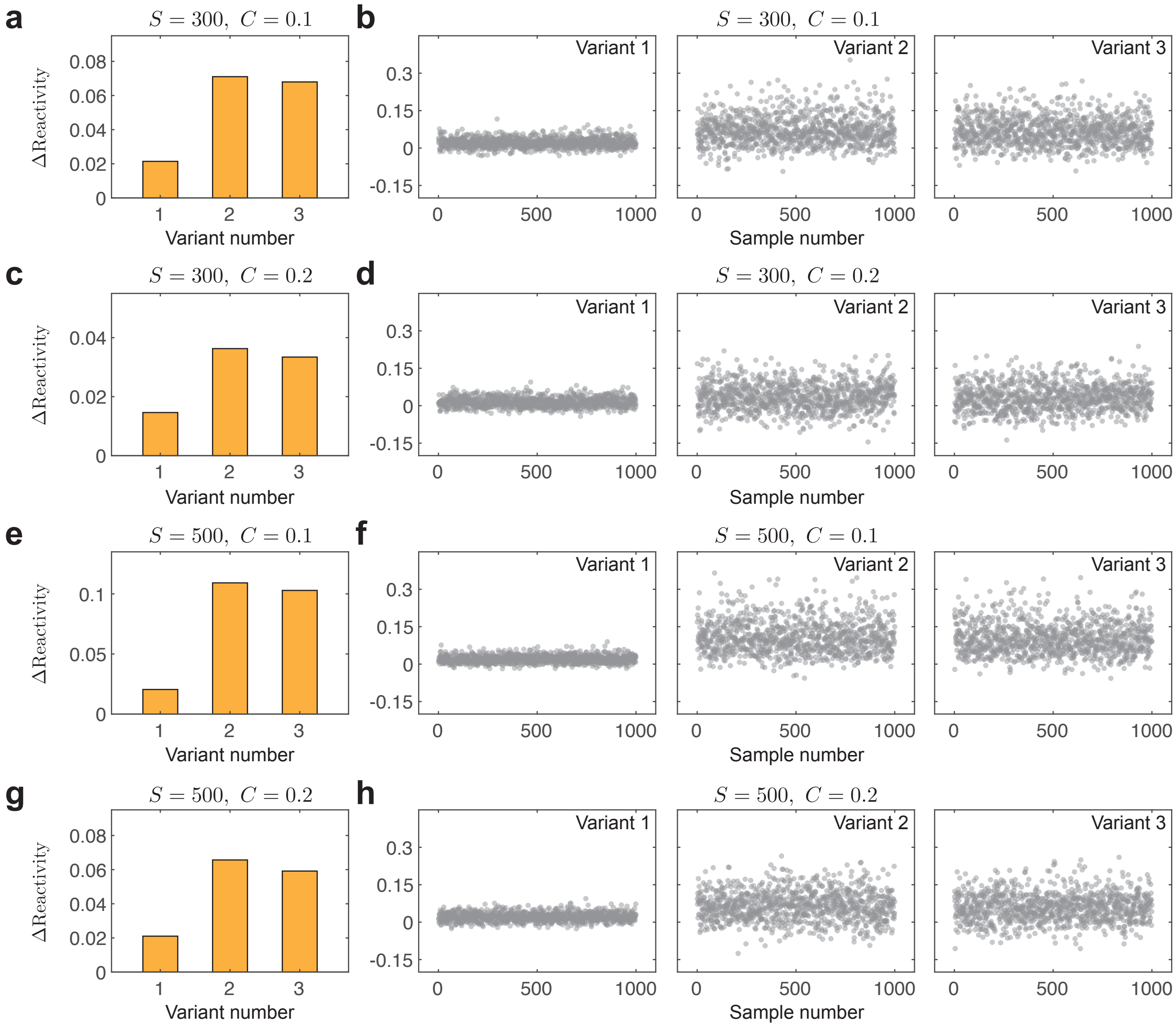

Supplemental Figure S6: Reactivity change of variants of cascade model. Variant 1 is interval cascade food web, variant 2 is cascade food web with broad degree distribution, and variant 3 is interval cascade food web with broad degree distribution. The change in reactivity, denoted as ΔReactivity, is calculated as $\Delta\text{Reactivity} = \mathcal{R}_{\text{variant}} - \mathcal{R}_{\text{cascade}}$. Each data in panels **a**, **c**, **e** and **g** is the average of 1000 randomly generated communities with the same set of parameters. Corresponding data of these 1000 communities are displayed in panels **b**, **d**, **f** and **h**, respectively. In this figure, we set $\sigma = 0.15$ and $d = 1$.

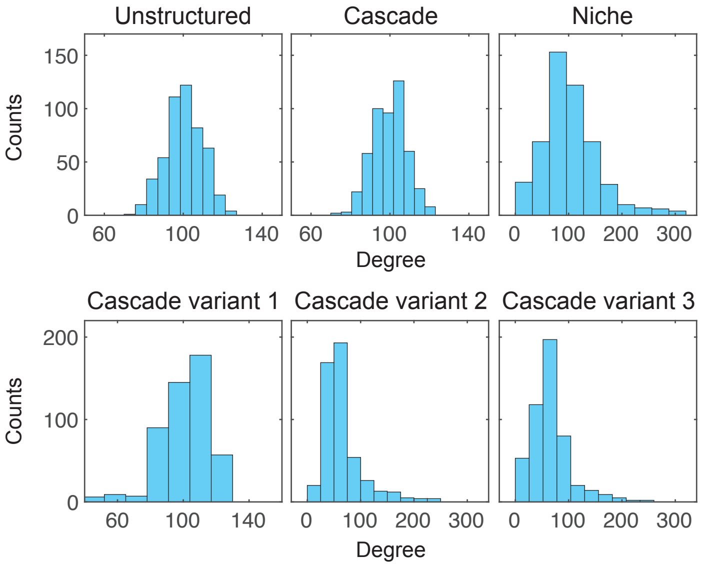

Supplemental Figure S7: Degree distributions of different food web models. The parameters in this figure are the same as Supplemental Fig. S5.

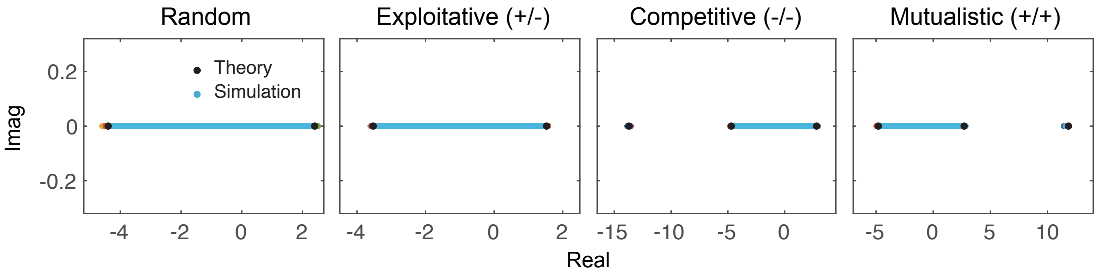

Supplemental Figure S8: Eigenvalue distributions of four classic types of communities with heterogeneous self-regulation strengths. Colored dots are eigenvalues of $\mathbf{H}$ of 20 randomly generated communities. Black dots are theoretical predications of the endpoints of the bulk of eigenvalues and the outlier. In this figure, self-regulation strengths are sampled from a uniform distribution. Community parameters are $S = 250,\ C = 0.25,\ \sigma = 0.25,\ d_{\text{mean}} = 1$ and $\sigma_d = 1$.

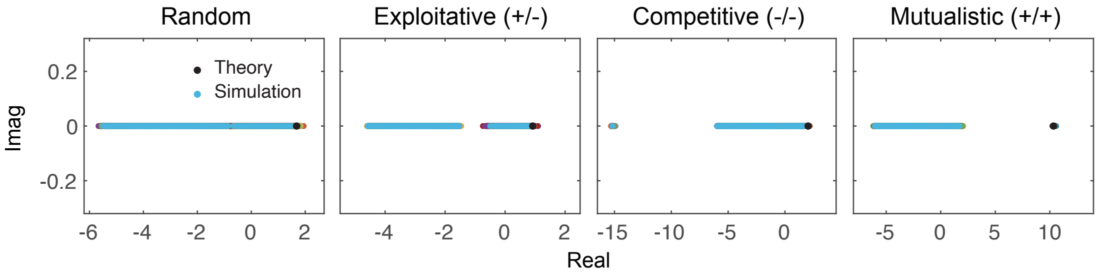

Supplemental Figure S9: Eigenvalue distributions of four classic types of communities where some species do not self-regulate. Colored dots are eigenvalues of $\mathbf{H}$ of 20 randomly generated communities. Black dots are theoretical predications of the rightmost eigenvalue of matrix $\mathbf{H}$. In this figure, $S = 250,\ C = 0.25,\ \sigma = 0.25,\ d = 3$ and 20% species do not self-regulate.

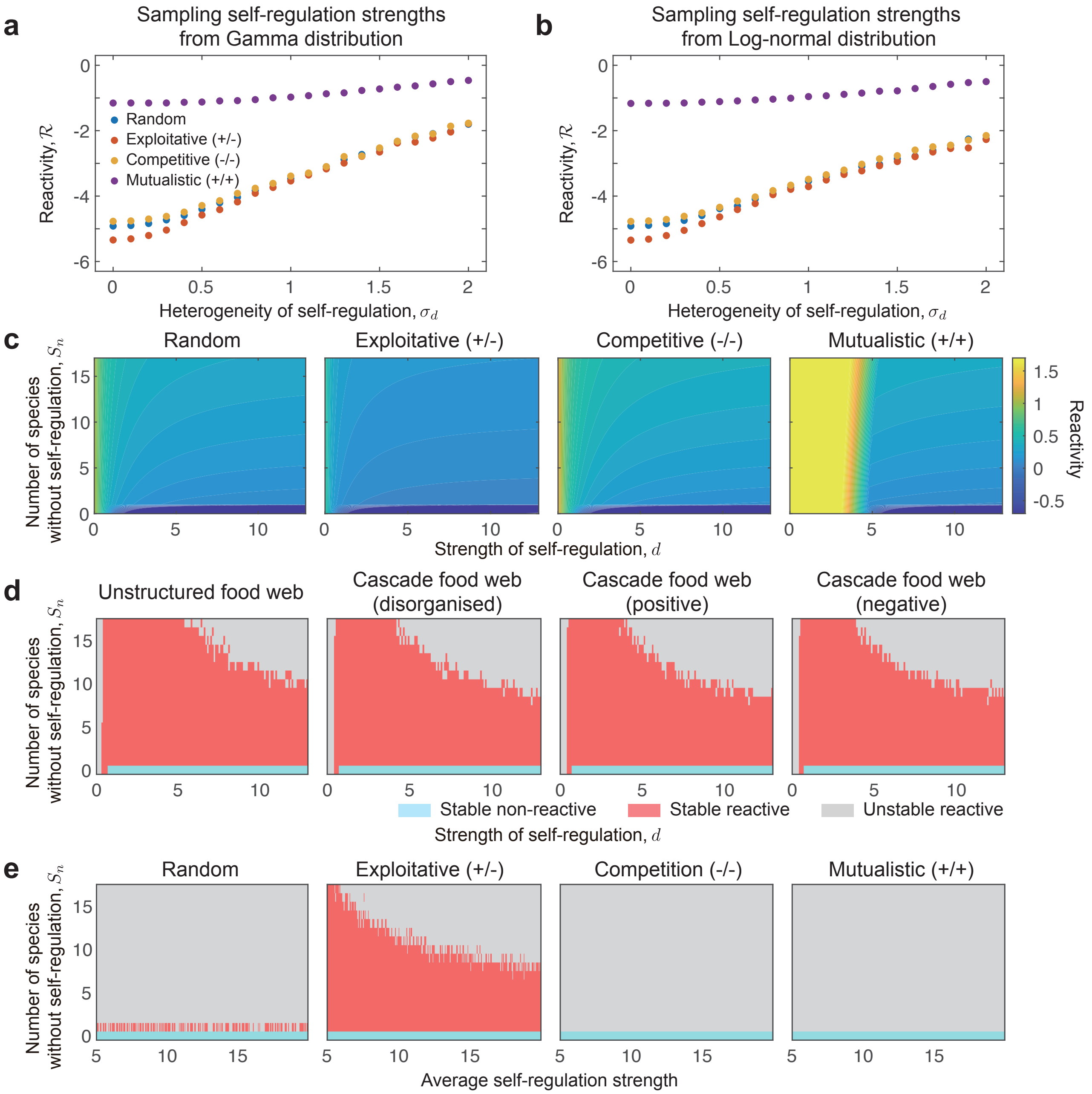

Supplemental Figure S10: **a** & **b**, Simulation results on the influence of heterogeneous self-regulation strengths on system reactivity. **c**, Theoretical results on the influence of non-self-regulating species on system reactivity. Note that here self-regulating species have homogeneous self-regulation strengths. **d**, Simulation results on the influence of non-self-regulating species on the reactivity of cascade food webs. The term 'disorganised' refers to the case where self-regulation strengths and trophic levels are not related, 'positive' refers to the case where species at higher trophic levels self-regulate, and 'negative' refers to the case where species at lower trophic levels self-regulate. **e**, Simulation results on the influence of non-self-regulating species on system reactivity. Note that here self-regulation strengths of self-regulating species are sampled from a uniform distribution with standard deviation 0.3. In (d) and (e), Communities in the blue region are on average stable and non-reactive, communities in the red region are on average stable and reactive, while communities in the grey region are on average unstable and reactive. Each data point in the simulation results is an average of 50 randomly constructed communities for the specific combination of community parameters. Community parameters are the same as those in Fig. 5.

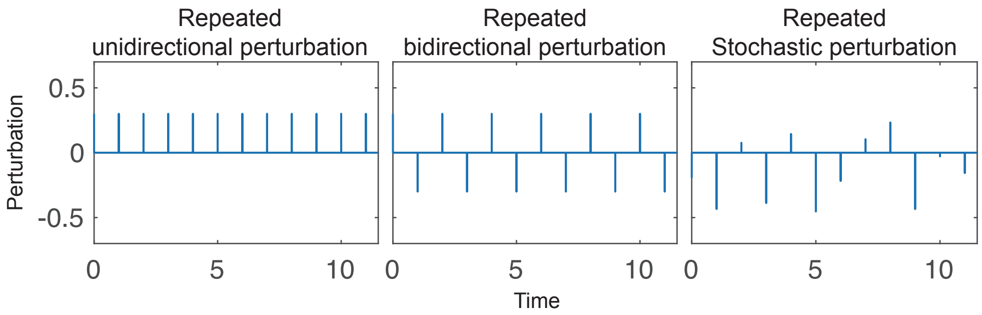

Supplemental Figure S11: Different types of frequent perturbation evaluated in our paper. In the case of repeated unidirectional perturbations, species abundances of perturbed species are increased (or decreased) by the same amplitude with a fixed frequency. In the case of repeated bidirectional perturbations, species abundances of perturbed species are increased and decreased alternatively by the same amplitude with a fixed frequency. In the case of repeated stochastic perturbation, species abundances of perturbed species are increased or decreased with a fixed frequency, and the perturbation at each time point is sampled from a random distribution (in this figure, normal distribution).

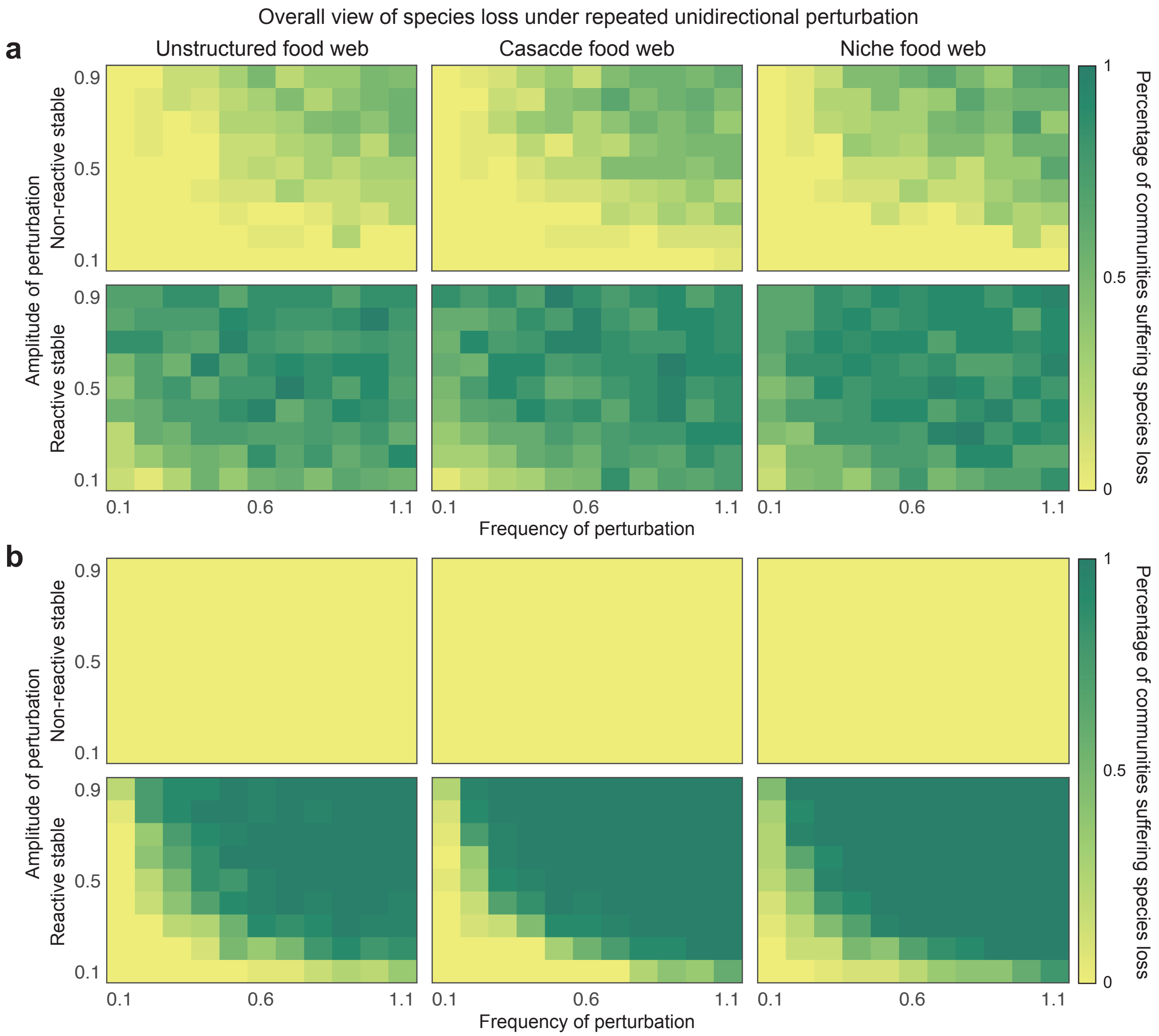

Supplemental Figure S12: Overall view of species loss in typical food web models under repeated unidirectional perturbations. Colours represent the percentage of communities suffering species loss from numerical simulations. To obtain the corresponding percentage, we randomly construct 20 communities and count how many communities can persist under frequent perturbations. Colour close to green suggests a higher percentage, while colour close to yellow suggests a lower percentage. In this figure, community dynamics come from the generalised Lotka-Volterra model. In **a**, community size $S = 10$, per capita self-regulation strength $s = 0.1$ for non-reactive stable case, and $s = 0.01$ for reactive stable case. In **b**, community size $S = 50$, per capita self-regulation strength $s = 1$ for non-reactive stable case, and $s = 0.1$ for reactive stable case. Other community parameters are the same as those in Fig. 6b. Repeated unidirectional perturbations are imposed by increasing the abundances of randomly picked 60% species with a fixed frequency. Simulation time is 500 unit time. In the simulations, we regard a species as extinct if its abundance is less than 1% of its equilibrium abundance.

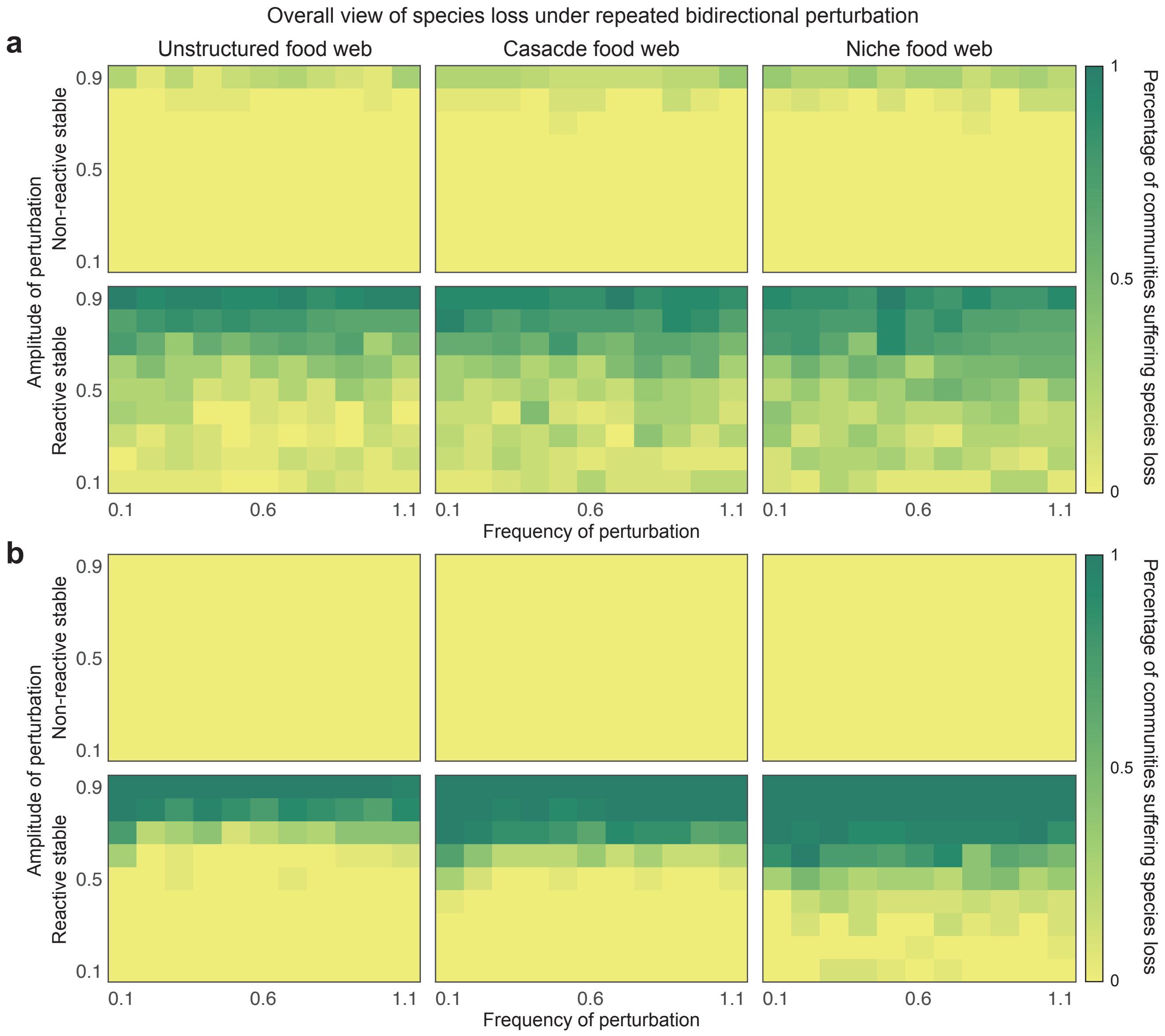

Supplemental Figure S13: Overall view of species loss in typical food web models under repeated bidirectional perturbations. Colours represent the percentage of communities suffering species loss from numerical simulations. To obtain the corresponding percentage, we randomly construct 20 communities and count how many communities can persist under frequent perturbations. Colour close to green suggests a higher percentage, while colour close to yellow suggests a lower percentage. In this figure, community dynamics come from the generalised Lotka-Volterra model. In **a**, community size $S = 10$, per capita self-regulation strength $s = 0.1$ for non-reactive stable case, and $s = 0.01$ for reactive stable case. In **b**, community size $S = 50$, per capita self-regulation strength $s = 1$ for non-reactive stable case, and $s = 0.1$ for reactive stable case. Other community parameters are the same as those in Fig. 6b. Repeated bidirectional perturbations are imposed by increasing and decreasing the abundances of randomly picked 60% species alternatively with a fixed frequency. Simulation time is 500 unit time. In the simulations, we regard a species as extinct if its abundance is less than 1% of its equilibrium abundance.

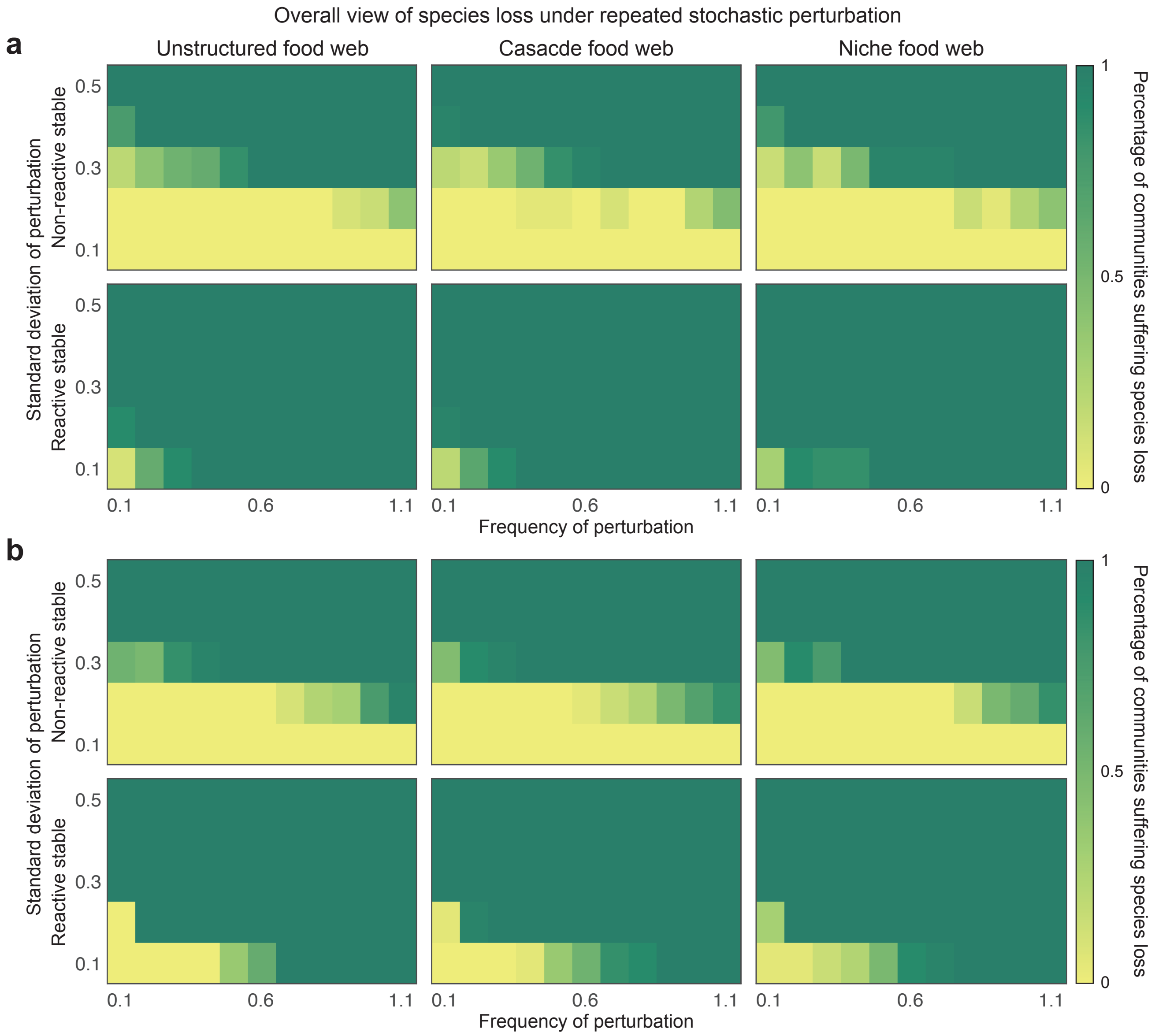

Supplemental Figure S14: Overall view of species loss in typical food web models under repeated stochastic perturbations. Colours represent the percentage of communities suffering species loss from numerical simulations. To obtain the corresponding percentage, we randomly construct 20 communities and count how many communities can persist under frequent perturbations. Colour close to green suggests a higher percentage, while colour close to yellow suggests a lower percentage. In this figure, community dynamics come from the generalised Lotka-Volterra model. In **a**, community size $S = 10$, per capita self-regulation strength $s = 0.1$ for non-reactive stable case, and $s = 0.01$ for reactive stable case. In **b**, community size $S = 50$, per capita self-regulation strength $s = 1$ for non-reactive stable case, and $s = 0.1$ for reactive stable case. Other community parameters are the same as those in Fig. 6b. In this figure, abundances of perturbed species are increased or decreased with a fixed frequency, and perturbation at each time point is generated by a normal distribution with mean 0. Randomly picked 60% species are perturbed. Simulation time is 500 unit time. In the simulations, we regard a species as extinct if its abundance is less than 1% of its equilibrium abundance.

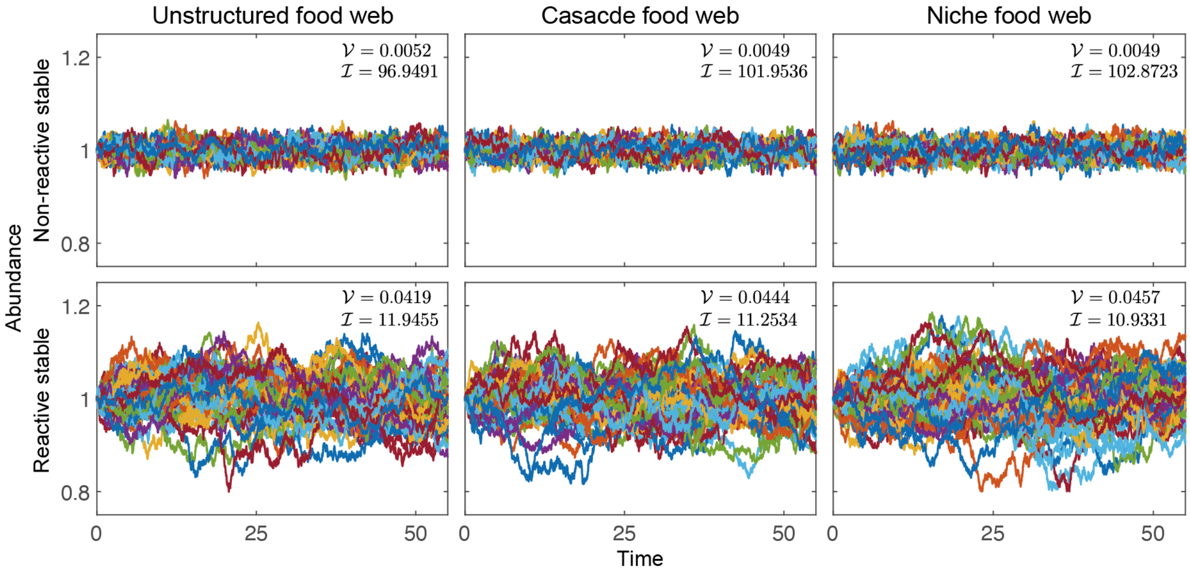

Supplemental Figure S15: Responses of different perturbed linearised food web models under immigration type perturbations ($\alpha = 0$). Each line in this figure captures the change in abundance of each species. $\mathcal{V}$ quantifies the degree of variability, while $\mathcal{I}$ quantifies the degree of invariability. Compared with non-reactive stable communities (upper row), reactive stable communities have relatively high variability (correspondingly, relatively low invariability), indicating these communities are more prone to experience species loss or system collapse. Community parameters are: $S = 50,\ C = 0.2,\ \sigma = 0.05$. For non-reactive communities, we have $d = 1$. For reactive communities, we have $d = 0.1$. σ_{p} of each species is sampled from a uniform distribution $U\,[0.15, 0.25]$.

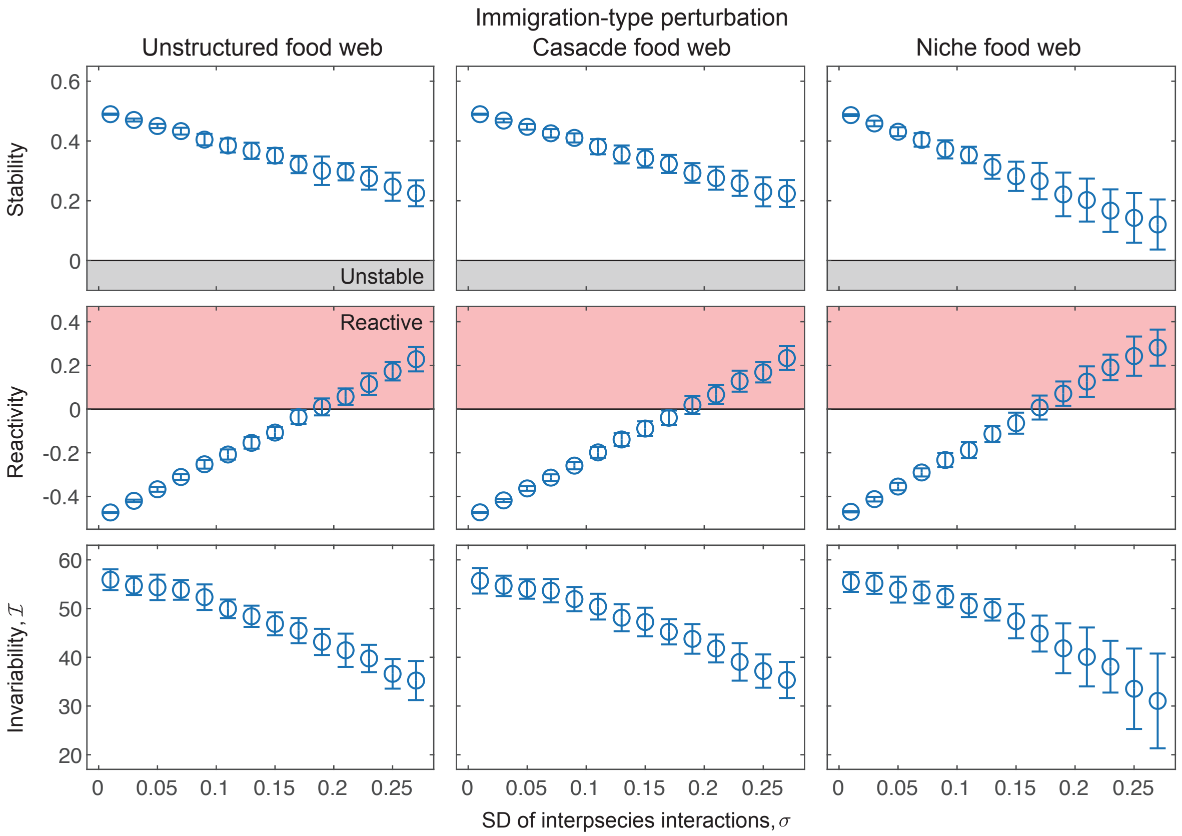

Supplemental Figure S16: Correlation between stability, reactivity and variability under immigration-type perturbations ($\alpha = 0$). Each dot in this figure is an average of 50 randomly constructed communities for the specific parameter combination. Each error bar denotes the standard deviation of these 50 communities. Community parameters are: $S = 50$, $C = 0.2$, $d = 0.5$. σ_p of each species is sampled from a uniform distribution $U\,[0.15, 0.25]$.

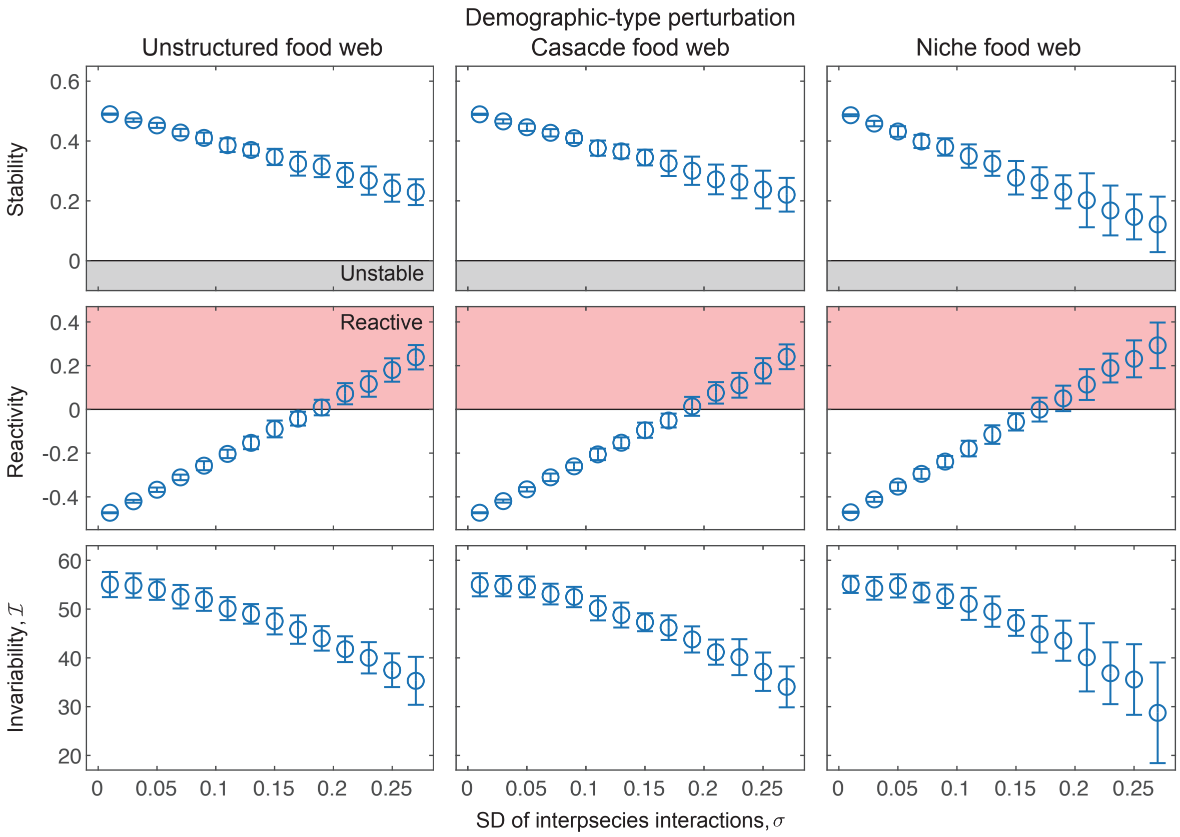

Supplemental Figure S17: Correlation between stability, reactivity and variability under demographic-type perturbations ($\alpha = 1$). Each dot in this figure is an average of 50 randomly constructed communities for the specific parameter combination. Each error bar denotes the standard deviation of these 50 communities. Community parameters are: $S = 50,\ C = 0.2,\ d = 0.5$. σ_{p} of each species is sampled from a uniform distribution $U\,[0.15, 0.25]$.

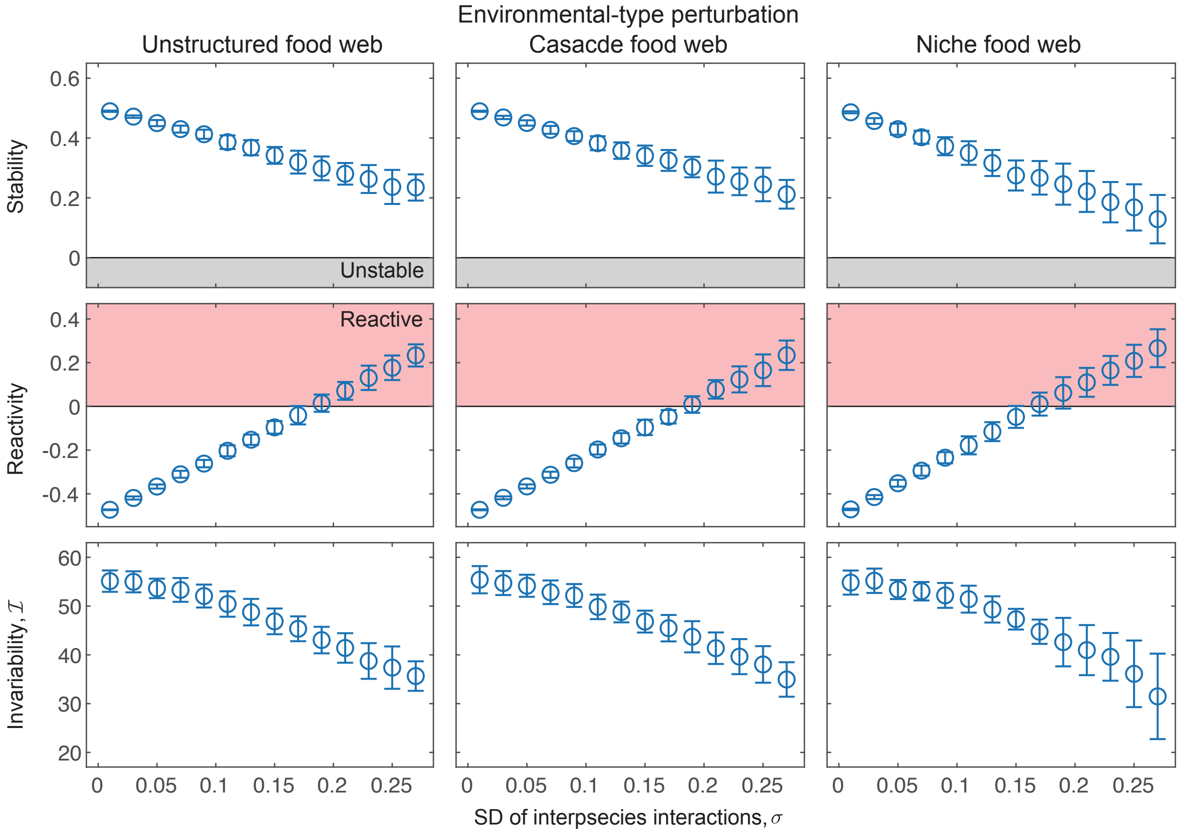

Supplemental Figure S18: Correlation between stability, reactivity and variability under environmental-type perturbations ($\alpha = 2$). Each dot in this figure is an average of 50 randomly constructed communities for the specific parameter combination. Each error bar denotes the standard deviation of these 50 communities. Community parameters are: $S = 50,\ C = 0.2,\ d = 0.5$. σ_{p} of each species is sampled from a uniform distribution $U\,[0.15, 0.25]$.

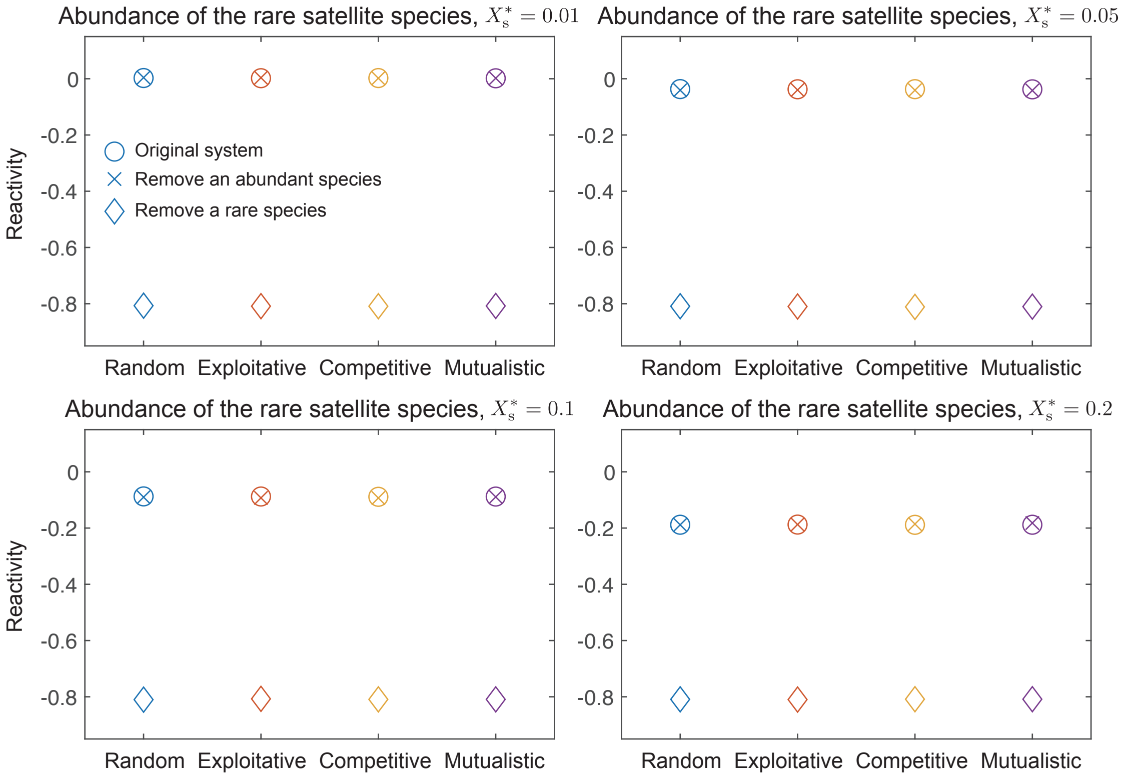

Supplemental Figure S19: Rare species influence system reactivity measures. Circles show the reactivity of original system with $S-1$ abundant species and 1 rare satellite species. Crosses show the system reactivity after randomly removing an abundant species. Diamonds show the system reactivity after removing the rare satellite species. Each symbol (circle, cross or diamond) is an average of 50 randomly generated communities with the same community parameters combination. The original system contains $S-1$ abundant species with unit abundance and 1 rare satellite species. The abundance of the satellite species is shown in the title of each panel. Community parameters are: $S = 100,\ C = 0.2,\ d = 1,\ \sigma = 0.05$.

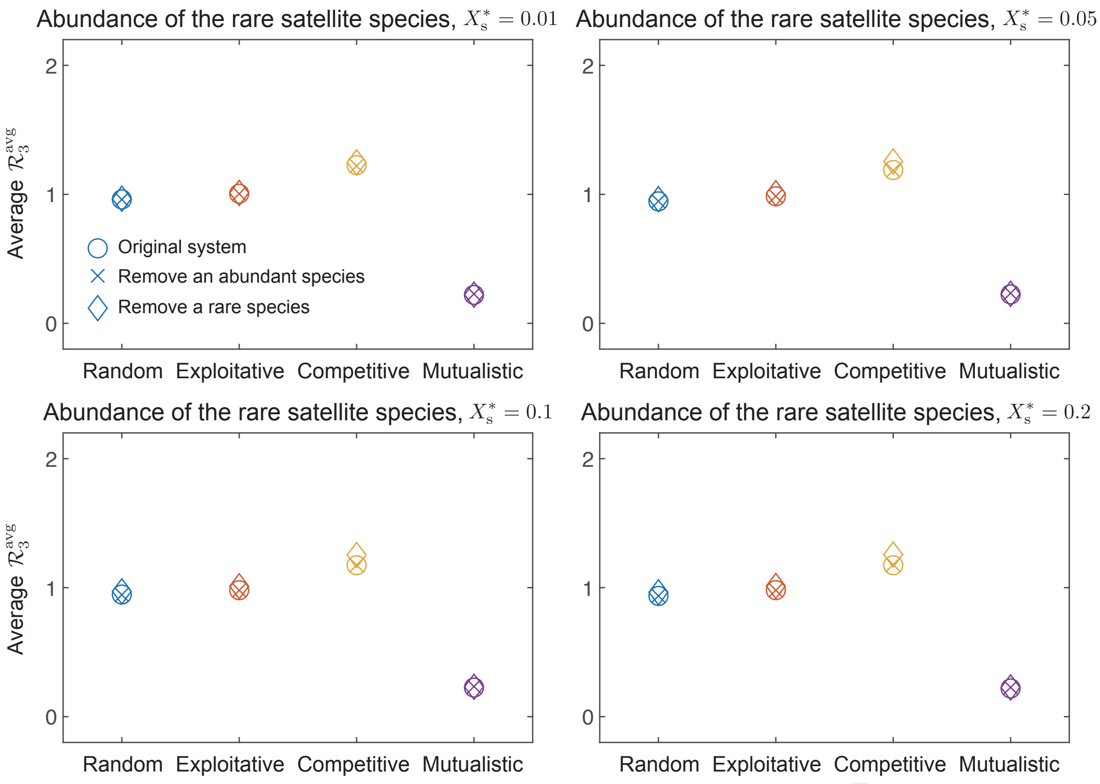

Supplemental Figure S20: Rare species have little influence on the observable short-term dynamics. Here we use average return rate over the interval $[0, 3]$ as a representative of observable short-term dynamics. Note that the system is perturbed at time 0. Circles show the average $\mathcal{R}_3^{avg}$ for the original system with S-1 abundant species and 1 rare satellite species. Crosses show the average $\mathcal{R}_3^{avg}$ after randomly removing an abundant species. Diamonds show the average $\mathcal{R}_3^{avg}$ after removing the rare satellite species. Each symbol (circle, cross or diamond) is an average of 50 randomly generated communities with the same community parameters combination. The perturbation strength is 1 and is randomly distributed on each species. Community parameters are the same as those in Supplemental Fig. S15.

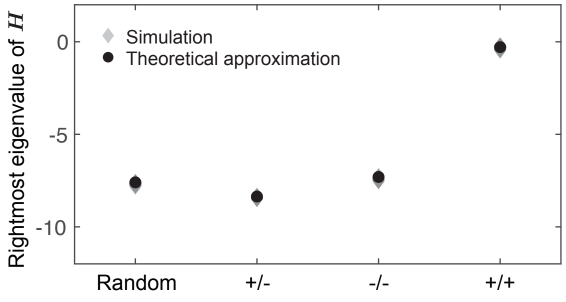

Supplemental Figure S21: Rightmost eigenvalues of $\mathbf{H}$ of four classic types of communities where species abundances are sampled from a uniform distribution. Grey dots are rightmost eigenvalues of $\mathbf{H}$ of 100 randomly generated communities. Black dots represent theoretical approximations of the rightmost eigenvalue of matrix $\mathbf{H}$. In this figure, $S = 200$, $C = 0.3$, $\sigma = 0.1$, $s = 5$. Species abundances are sampled from a uniform distribution with $\mu_X = 2$, $\sigma_X = 0.1$.

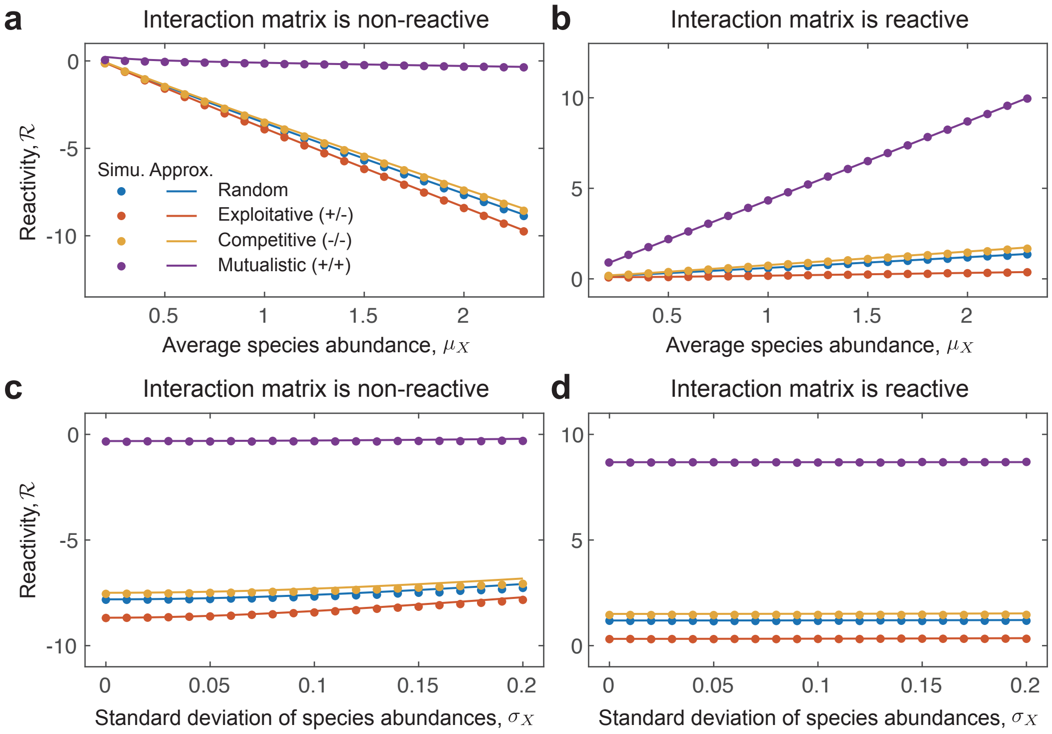

Supplemental Figure S22: Approximation of the reactivity of communities where species abundances are sampled from a uniform distribution. Dots in these panels are results from numerical simulations (Simu.) and each dot is an average of 50 randomly constructed communities with the same set of community parameters. Lines are results from theoretical approximation (Approx.). In **a** & **c**, $s = 5$. In **b** & **d**, $s = 0.5$. Other community parameters in (a) and (b) are $S = 200,\ C = 0.3,\ \sigma = 0.1,\ \sigma_X = 0.1$. Other community parameters in (c) and (d) are $S = 200,\ C = 0.3,\ \sigma = 0.1,\ \mu_X = 2$.

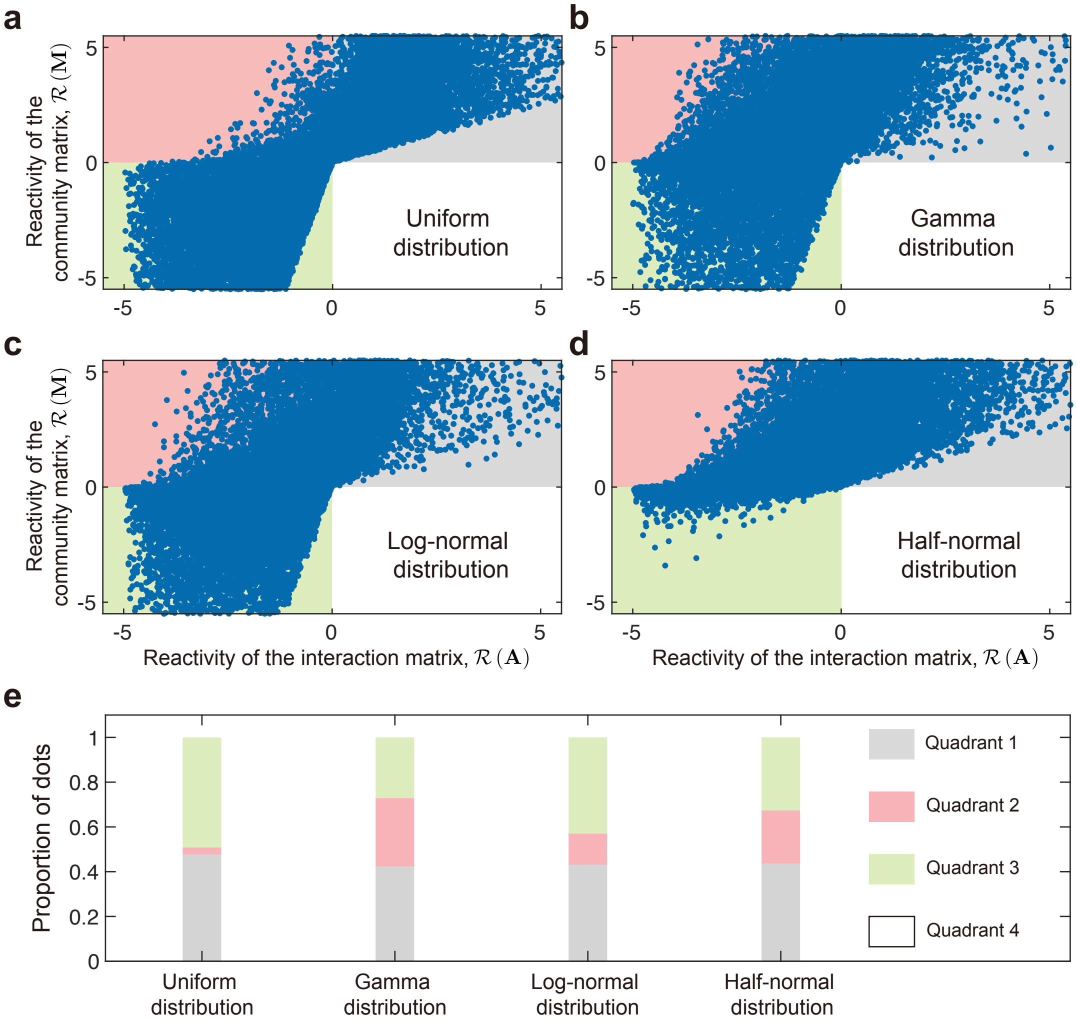

Supplemental Figure S23: Simulation results on the relationship between the reactivity of $\mathbf{A}$ and the reactivity of $\mathbf{M}$. **a-d**, Randomly generated communities with species abundances sampled from different random distributions. Blue Dots in the grey region (i.e., Quadrant 1) indicate that both $\mathbf{A}$ and $\mathbf{M}$ are reactive. Dots in the red region (i.e., Quadrant 2) indicate that $\mathbf{A}$ is non-reactive while $\mathbf{M}$ is reactive. Dots in the green region (i.e., Quadrant 3) indicate that both $\mathbf{A}$ and $\mathbf{M}$ are non-reactive. Dots in the white region (i.e., Quadrant 4) indicate that $\mathbf{A}$ is reactive while $\mathbf{M}$ is non-reactive. **e**, Proportions of dots in different quadrants of (a)-(d). Details about the community generation strategy are provided in Supplementary Note 8. These simulation results suggest that when $\mathbf{A}$ is reactive, then the corresponding $\mathbf{M}$ is reactive. However, when $\mathbf{A}$ is non-reactive, the corresponding $\mathbf{M}$ can be reactive or non-reactive.

Supplementary References

[1] Allesina, S. & Tang, S. Stability criteria for complex ecosystems. *Nature* **483**, 205–208 (2012).

[2] Allesina, S. & Tang, S. The stability–complexity relationship at age 40: a random matrix perspective. *Popul. Ecol.* **57**, 63–75 (2015).

[3] Barabás, G., Michalska-Smith, M. J. & Allesina, S. Self-regulation and the stability of large ecological networks. *Nat. Ecol. Evol.* **1**, 1870–1875 (2017).

[4] Nguyen, H. H. & O'Rourke, S. The elliptic law. *Int. Math. Res.* **2015**, 7620–7689 (2015).

[5] O'Rourke, S. & Renfrew, D. Low rank perturbations of large elliptic random matrices. *Electron. J. Probab.* **19**, 1–65 (2014).

[6] May, R. M. Will a large complex system be stable? *Nature* **238**, 413–414 (1972).

[7] Stein, R. R., Bucci, V., Toussaint, N. C., Buffie, C. G., Rätsch, G., Pamer, E. G., Sander, C. & Xavier, J. B. Ecological modeling from time-series inference: insight into dynamics and stability of intestinal microbiota. *PLoS Comput. Biol.* **9**, e1003388 (2013).

[8] Friedman, J., Higgins, L. M. & Gore, J. Community structure follows simple assembly rules in microbial microcosms. *Nat. Ecol. Evol.* **1**, 0109 (2017).

[9] Hu, J., Amor, D. R., Barbier, M., Bunin, G. & Gore, J. Emergent phases of ecological diversity and dynamics mapped in microcosms. *Science* **378**, 85–89 (2022).

[10] Allesina, S., Grilli, J., Barabás, G., Tang, S., Aljadeff, J. & Maritan, A. Predicting the stability of large structured food webs. *Nat. Commun.* **6**, 1–6 (2015).

[11] Grilli, J., Rogers, T. & Allesina, S. Modularity and stability in ecological communities. *Nat. Commun.* **7**, 1–10 (2016).

[12] Stouffer, D. B., Camacho, J. & Amaral, L. A. N. A robust measure of food web intervality. *Proc. Natl. Acad. Sci. U.S.A.* **103**, 19015–19020 (2006).

[13] Dunne, J. A., Williams, R. J. & Martinez, N. D. Food-web structure and network theory: the role of connectance and size. *Proc. Natl. Acad. Sci. U.S.A.* **99**, 12917–12922 (2002).

[14] Williams, R. J. & Martinez, N. D. Simple rules yield complex food webs. *Nature* **404**, 180–183 (2000).

[15] Allesina, S., Alonso, D. & Pascual, M. A general model for food web structure. *Science* **320**, 658–661 (2008).

[16] Cohen, J. E., Briand, F. & Newman, C. M. *Community Food Webs: Data and Theory* (Springer Science & Business Media, 2012).

[17] Chen, X. & Cohen, J. E. Transient dynamics and food-web complexity in the lotka-volterra cascade model. *Proc. R. Soc. Lond. B* **268**, 869–877 (2001).

[18] May, R. M. & McLean, A. R. *Theoretical Ecology: Principles and Applications* (Oxford University Press, 2007).

[19] Pimm, S. & Lawton, J. Number of trophic levels in ecological communities. *Nature* **268**, 329–331 (1977).

[20] Yodzis, P. The stability of real ecosystems. *Nature* **289**, 674–676 (1981).

[21] Rogers, T. Universal sum and product rules for random matrices. *J. Math. Phy.* **51**, 093304 (2010).

[22] Voight, J. *Quaternion Algebras* (Springer Nature, 2021).

[23] Arnoldi, J.-F., Loreau, M. & Haegeman, B. The inherent multidimensionality of temporal variability: how common and rare species shape stability patterns. *Ecol. Lett.* **22**, 1557–1567 (2019).

[24] Arnoldi, J.-F., Bideault, A., Loreau, M. & Haegeman, B. How ecosystems recover from pulse perturbations: A theory of short-to long-term responses. *J. Theor. Biol.* **436**, 79–92 (2018).
