# Supplementary material for: Reactivity of complex communities can be more important than stability
Source: Nat Commun. 2023 Nov 8;14:7204. doi: 10.1038/s41467-023-42580-0 (PMC10632443; doi:10.1038/s41467-023-42580-0)
Supplement: Supplementary file 3 — Reporting Summary [file 41467_2023_42580_MOESM3_ESM.pdf]

nature portfolio

Corresponding author(s): Kevin R. Foster, Aming Li

Last updated by author(s): Oct 12, 2023

Reporting Summary

Nature Portfolio wishes to improve the reproducibility of the work that we publish. This form provides structure for consistency and transparency in reporting. For further information on Nature Portfolio policies, see our Editorial Policies and the Editorial Policy Checklist.

Statistics

For all statistical analyses, confirm that the following items are present in the figure legend, table legend, main text, or Methods section.

n/a	Confirmed	
☐	☒	The exact sample size (*n*) for each experimental group/condition, given as a discrete number and unit of measurement
☐	☒	A statement on whether measurements were taken from distinct samples or whether the same sample was measured repeatedly
☒	☐	The statistical test(s) used AND whether they are one- or two-sided<br>*Only common tests should be described solely by name; describe more complex techniques in the Methods section.*
☒	☐	A description of all covariates tested
☒	☐	A description of any assumptions or corrections, such as tests of normality and adjustment for multiple comparisons
☐	☒	A full description of the statistical parameters including central tendency (e.g. means) or other basic estimates (e.g. regression coefficient) AND variation (e.g. standard deviation) or associated estimates of uncertainty (e.g. confidence intervals)
☒	☐	For null hypothesis testing, the test statistic (e.g. *F*, *t*, *r*) with confidence intervals, effect sizes, degrees of freedom and *P* value noted<br>*Give P values as exact values whenever suitable.*
☒	☐	For Bayesian analysis, information on the choice of priors and Markov chain Monte Carlo settings
☒	☐	For hierarchical and complex designs, identification of the appropriate level for tests and full reporting of outcomes
☒	☐	Estimates of effect sizes (e.g. Cohen's *d*, Pearson's *r*), indicating how they were calculated

Our web collection on statistics for biologists contains articles on many of the points above.

Software and code

Policy information about availability of computer code

Data collection	The data analysed in our study (except those in Fig. 3d) are simulation data and can be generated by the codes provided. The codes are written with MathWorks Matlab R-2020b and are now available at https://github.com/Pawn053/Reactivity-of-complex-ecosystems. Data analysed in Fig. 3d are publicly available form corresponding references. These references are provided in the manuscript.
Data analysis	Data were analysed using MathWorks Matlab R-2020b.

For manuscripts utilizing custom algorithms or software that are central to the research but not yet described in published literature, software must be made available to editors and reviewers. We strongly encourage code deposition in a community repository (e.g. GitHub). See the Nature Portfolio guidelines for submitting code & software for further information.

Data

Policy information about availability of data

All manuscripts must include a data availability statement. This statement should provide the following information, where applicable:

- Accession codes, unique identifiers, or web links for publicly available datasets
- A description of any restrictions on data availability
- For clinical datasets or third party data, please ensure that the statement adheres to our policy

The data analysed in our study (except those in Fig. 3d) are simulation data and can be generated by the codes provided. The codes are written with MathWorks Matlab R-2020b and are now available at https://github.com/Pawn053/Reactivity-of-complex-ecosystems. Data analysed in Fig. 3d are publicly available form corresponding references. These references are provided in the manuscript.

Human research participants

Policy information about studies involving human research participants and Sex and Gender in Research.

Reporting on sex and gender	N/A
Population characteristics	N/A
Recruitment	N/A
Ethics oversight	N/A

Note that full information on the approval of the study protocol must also be provided in the manuscript.

Field-specific reporting

Please select the one below that is the best fit for your research. If you are not sure, read the appropriate sections before making your selection.

☐ Life sciences ☐ Behavioural & social sciences ☒ Ecological, evolutionary & environmental sciences

For a reference copy of the document with all sections, see nature.com/documents/nr-reporting-summary-flat.pdf

Ecological, evolutionary & environmental sciences study design

All studies must disclose on these points even when the disclosure is negative.

Study description	Our study provides a theoretical framework to analyse the reactivity of complex ecosystems. We study the influence of interspecies interaction types, interspecies interaction strength on system reactivity, evaluate the importance of reactivity for communities with different interaction types, and show that reactivity can be a better extinction indicator than stability when facing frequent perturbations.
Research sample	This is a theoretical study with simulated ecological communities.
Sampling strategy	We used theoretical models to simulate ecological communities.
Data collection	Empirical data are publicly available, other data were collected from numerical simulations or theoretical analyses, as described in the manuscript.
Timing and spatial scale	N/A
Data exclusions	No data was excluded.
Reproducibility	All data can be reproduced using the code provided.
Randomization	N/A
Blinding	N/A

Did the study involve field work? ☐ Yes ☒ No

Reporting for specific materials, systems and methods

We require information from authors about some types of materials, experimental systems and methods used in many studies. Here, indicate whether each material, system or method listed is relevant to your study. If you are not sure if a list item applies to your research, read the appropriate section before selecting a response.

Materials & experimental systems

n/a	Involved in the study
☒	☐ Antibodies
☒	☐ Eukaryotic cell lines
☒	☐ Palaeontology and archaeology
☒	☐ Animals and other organisms
☒	☐ Clinical data
☒	☐ Dual use research of concern

Methods

n/a	Involved in the study
☒	☐ ChIP-seq
☒	☐ Flow cytometry
☒	☐ MRI-based neuroimaging
